# Cardiovascular disease burden and risk factor management in cancer survivors: insights into a multiethnic, socioeconomically deprived urban population

**DOI:** 10.1136/heartjnl-2024-325309

**Published:** 2025-03-13

**Authors:** Liliana Szabo, Jackie Cooper, Dorina-Gabriela Condurache, Isabel Dostal, Gracia Andriamiadana, Rohini Mathur, Fiona M Walter, Mamas A Mamas, Charlotte H Manisty, Nicholas C Harvey, Stefan Neubauer, Steffen E Petersen, John Robson, Zahra Raisi-Estabragh

**Affiliations:** 1 William Harvey Research Institute, NIHR Barts Biomedical Research Centre, Queen Mary University of London, London, UK; 2 Barts Heart Centre, Saint Bartholomew’s Hospital Barts Heart Centre, London, UK; 3 Semmelweis University Heart and Vascular Centre, Budapest, Hungary; 4 Wolfson Institute of Population Health, Queen Mary University of London, London, UK; 5 Department of Public Health and Primary Care, University of Cambridge, Cambridge, UK; 6 Keele Cardiovascular Research Group, Keele University, Newcastle-under-Lyme, UK; 7 Institute of Population Health, University of Manchester, Manchester, UK; 8 Institute of Cardiovascular Science, University College London, London, UK; 9 MRC Lifecourse Epidemiology Unit, University of Southampton, Southampton, UK; 10 NIHR Southampton Biomedical Research Centre, University of Southampton, Southampton, UK; 11 Division of Cardiovascular Medicine, Radcliffe Department of Medicine, University of Oxford, National Institute for Health Research Oxford Biomedical Research Centre, Oxford University Hospitals NHS Foundation Trust, Oxford, UK; 12 British Heart Foundation Data Science Centre, Health Data Research UK, London, UK

**Keywords:** Epidemiology, Cardiovascular Diseases, Cohort Studies, Risk Factors, Outcome Assessment, Health Care

## Abstract

**Background:**

Cardiovascular disease (CVD) burden and risk factor management among cancer survivors, especially in socioeconomically deprived, multiethnic populations, remain understudied. This study examines CVD burden and risk factor control in survivors of 20 cancer types within a diverse urban population.

**Methods:**

This matched cohort study used electronic health records from 127 urban primary care practices. Cancer survivors were matched to non-cancer comparators at a 1:4 ratio. Cancer and CVD diagnoses were defined using standard clinical code sets. Sociodemographic variables, lifestyle behaviours, blood pressure, cholesterol levels and statin prescriptions were analysed. Multivariable regression evaluated associations between cancer history, CVD prevalence and risk factor control.

**Results:**

The cohort included 18 839 cancer survivors (43% men, average age 64±15 years), with high ethnic diversity (48% White, 24% Black, 22% Asian) and high deprivation levels. Cancer survivors had elevated odds of all CVDs considered, independent of shared risk factors. Heart failure was more common in haematological (OR 2.12; 95% CI 1.44 to 3.09) and breast cancer survivors (OR 1.38; 95% CI 1.16 to 1.64). Patients with bladder (OR 1.50; 95% CI 1.20 to 1.87) and lung cancer (OR 1.44; 95% CI 1.09 to 1.87) had higher odds of ischaemic heart disease. Venous thromboembolism risk was highest in ovarian cancer (OR 5.72; 95% CI 3.54 to 9.32). Blood pressure control was slightly better in cancer survivors (OR 0.92; 95% CI 0.87 to 0.97), yet one in three patients did not meet guideline-directed targets. Statin use and cholesterol management were similar between survivors and controls, but disparities were observed within certain ethnic groups.

**Conclusion:**

Cancer survivors have an elevated risk of CVD, with variations by cancer type and ethnicity. Despite comparable or slightly better control of major risk factors, a significant proportion of cancer survivors do not achieve guideline-recommended targets, highlighting the need for optimised management strategies, particularly in high-risk subgroups.

**WHAT IS ALREADY KNOWN ON THIS TOPIC**

- Cancer survivors have a heightened risk of cardiovascular disease (CVD), attributed to shared risk factors and cancer therapies. However, data on CVD burden and risk factor control in diverse, multiethnic populations of cancer survivors are limited.

**WHAT THIS STUDY ADDS**

- This study highlights the elevated cardiovascular risk faced by cancer survivors compared with non-cancer controls, with specific cancer types (eg, haematological, breast, bladder and lung cancers) showing particularly heightened risks.
- Despite comparable or slightly better control of major risk factors, one in three cancer survivors do not achieve recommended blood pressure targets, indicating a significant treatment gap.
- Ethnic disparities were observed, with Asian survivors at higher risk of venous thromboembolism and Black survivors showing elevated low-density lipoprotein cholesterol levels, underscoring the need for tailored management strategies.

**HOW THIS STUDY MIGHT AFFECT RESEARCH, PRACTICE OR POLICY**

- These findings underscore the importance of increased cardiovascular vigilance in cancer survivors, particularly in high-risk subgroups and ethnic minority populations.

## Introduction

 Cancer survival has improved and doubled in the UK over the last 50 years.^1^ People with past cancer have a heightened risk of cardiovascular diseases (CVDs),^2^ attributed to shared risk factors,^3^ cardiotoxicity of cancer therapies^4^ and biological processes related to the cancer itself.^5^

Metrics of better cardiovascular health translate to similar reductions in cardiovascular mortality in patients with cancer as in the general population.^6^ Preventive strategies are particularly important for patients with cancer who are also more likely to have indications for risk reduction measures, such as statin therapy.^7^

Ethnicity and economic deprivation are key social determinants of health.^8^ Few studies report on the cardiovascular healthcare needs of cancer survivors from multiethnic populations and those with high levels of deprivation. Such analyses are key for informing healthcare planning, improving the cardiovascular health of cancer survivors and reducing health inequalities.

We analysed primary care data from an ethnically and socioeconomically diverse cohort of over 1.2 million patients in London, England. This population is among the most socially deprived in England and comprises an ethnically diverse population, with over half the residents from ethnicities other than White.^9^ Despite the socioeconomic disadvantages of the population, primary care services in these regions have above-national average performance in cardiovascular risk management.^10^

The study aimed to (1) describe the distribution and burden of key cardiovascular risk factors and diseases among survivors of 20 cancers and (2) assess gaps in hypertension and cholesterol management among cancer survivors with hypertension and ischaemic CVDs, respectively. We evaluated differences in risk factor management between cancer survivors and non-cancer controls while modelling the influence of ethnicity and deprivation.

## Methods

### Setting and study population

We performed a matched cohort study using data extracted from general practitioner electronic healthcare records. The dataset included over 1.2 million adults from all 127 urban general practices using the same electronic record system (Egton Medical Information Systems) in four east London boroughs: Tower Hamlets, Newham, Hackney and Waltham Forest. The deidentified data included coded patient demographics, clinical diagnoses, measurements and prescription data. The study cohort included adults aged ≥18 years, continuously registered for at least 12 months prior to the data extraction to minimise bias related to missing health records. Standard national codesets were available for all clinical variables from NHS Digital.^11^ The four study localities are ranked among the top performing areas in the England National Quality and Outcome Framework for hypertension and lipid management.^12^

### Patient and public involvement

This research was shaped by consultations with the Barts Cardio-Oncology Research Engagement group, whose input guided the study’s focus and design. We have shared preliminary findings with the group, and we plan to disseminate the final results through presentations, publications and other accessible formats.

### Cancer status

Cancer status was defined using standardised SNOMED CT (Systematized Nomenclature of Medicine—Clinical Terms) codes,^11^ detailed in online supplemental table 1. Patients were grouped into a composite ‘any cancer’ category and into subcategories of 20 cancer sites, covering the most common adult cancers.^2^ The first recorded cancer site was taken as the primary site. The following cancer sites were included: oral cavity, oesophageal, stomach, colorectal, liver, pancreas, lung, malignant melanoma, breast, cervix, uterus, ovarian, prostate, kidney, bladder, brain/CNS, thyroid, haematological cancers including non-Hodgkin's lymphoma, multiple myeloma, leukaemia.

### Matching of cancer survivors and non-cancer comparators

Patients without any record of cancer were considered as potential comparators. Each cancer survivor was matched on age and sex to four non-cancer comparators. Exact matching was used for sex, and nearest neighbour matching for age.

### Cardiovascular morbidities

We defined prevalent cardiovascular risk factors and diseases at time of extract using SNOMED CT codes (online supplemental table 2). The following conditions were included: ischaemic heart disease, peripheral artery disease, ischaemic stroke/transient ischaemic attack, heart failure, atrial fibrillation (AF) and venous thromboembolism (VTE), diabetes, hypertension and chronic kidney disease (CKD, stage 3 or 4).

### Demographics and lifestyle characteristics

Demographic data included age, sex and self-reported ethnicity, which were categorised according to UK census categories^13^: White, Mixed, Asian, Black and ‘Other’ ethnic groups. Deprivation was defined using the Index of Multiple Deprivation (IMD).^14^ The latest smoking status was used to categorised patients into current, past and never smokers. For alcohol use, the latest recorded weekly alcohol consumption (in units) was extracted from the electronic health records, with alcohol misuse flagged if noted in clinical documentation. The latest recorded body mass index (BMI) was extracted.

### Hypertension management

We evaluated the management of patients with a clinical diagnosis of hypertension, using three control indicators: (1) antihypertensive prescriptions, (2) clinic blood pressure readings and (3) adherence to guideline-directed blood pressure targets. Antihypertensive drugs prescribed in the preceding 12 months were grouped into: angiotensin-converting enzyme inhibitors/angiotensin receptor blockers, calcium channel blockers, thiazide diuretics and others (including beta blockers, spironolactone/potassium-sparing diuretics, alpha-blockers and loop diuretics). The average clinic blood pressure was calculated from the latest three readings recorded prior to the extract date and within the preceding 3 years. Optimal blood pressure control was defined based on National Institute for Health and Care Excellence (NICE)^15^ guidelines, as <140/90 mm Hg for people under 80 years and <150/90 mm Hg for those 80 and older.

### Cholesterol management

For patients with diagnosed ischaemic CVDs (myocardial infarction, angina, peripheral artery disease, stroke, transient ischaemic attacks), cholesterol management was evaluated by: (1) any statin prescription, (2) optimal intensity statin prescribing and (3) serum lipids. In accordance with NICE guidelines,^16^ we defined optimal statin treatment as atorvastatin 80 mg in patients under 75 years old and without CKD, and atorvastatin 20 mg in patients over 75 years old or with CKD. Patients not on statin or lower intensity therapy, based on prescriptions issued in the preceding 12 months, were classed as having suboptimal control. Serum lipids were based on the latest blood sample results available, considering total cholesterol, high-density lipoprotein cholesterol (HDL-C) and low-density lipoprotein cholesterol (LDL-C).

### Statistical analysis

All analyses were performed using R (V.4.3.0). Four controls were matched to each cancer case on age and sex, using exact matching for sex and nearest neighbour matching for age. Continuous variables are presented as mean and SD for continuous variables and categorical variables as numbers with percentages. Firth logistic regression assessed associations between cancer status (exposure) and cardiovascular outcomes, adjusting for age, sex, ethnicity, deprivation, smoking, BMI and alcohol. Firth’s bias-adjusted estimates of ORs were obtained to reduce any bias in the estimated coefficients due to low event rates or overfitting. Missing covariate data (20%) were imputed using Single Centre Imputation from Multiple Chained Equations and sensitivity analysis was performed using complete case analysis. For hypertension and cholesterol management, logistic regression was used for binary outcomes and linear regression for continuous outcomes. Suboptimal blood pressure and statin therapy analyses were limited to cancer sites with sufficient power (>670 and >550 cases, respectively). As cholesterol endpoints were not normally distributed and did not become normal on log transformation, an additional sensitivity analysis was run using quantile regression to calculate the difference in medians between those with and without cancer. Effect modification by ethnicity and deprivation was tested using interaction term analysis and time from cancer diagnosis was assessed as a main effect. In cases where significant interaction effects were observed, the nature of this was investigated using stratified analyses.

All results are reported as OR or beta coefficients alongside 95% CIs and p values, with multiple testing correction using false discovery rate (FDR) set at 5%.

## Results

### Baseline characteristics

#### Whole sample

**Figure 1:**
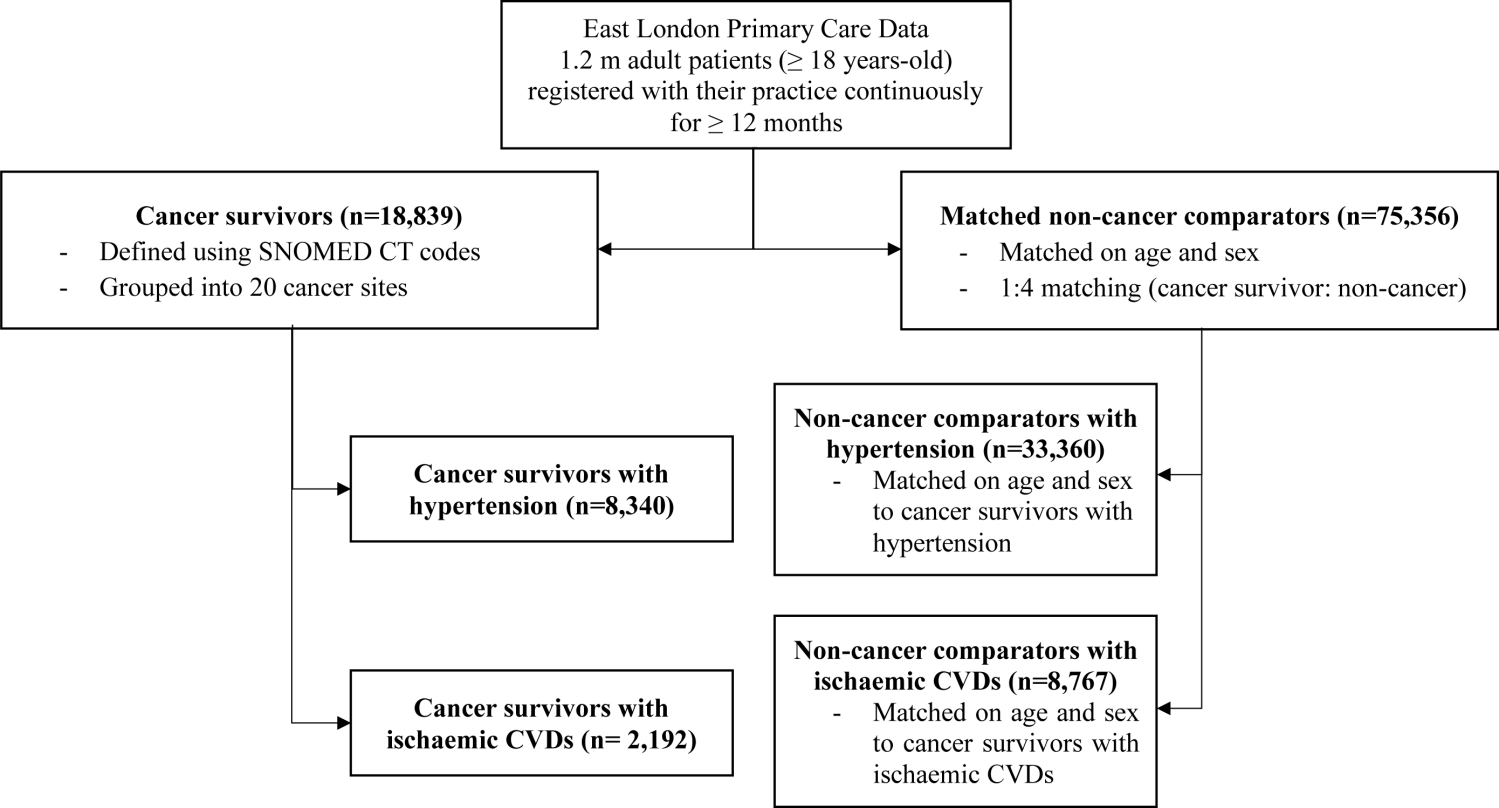
Flowchart of study participants. Flowchart illustrating the study design and cohort selection from the East London Primary Care Database, which includes 1.2 million patients continuously registered with their GP. The study population consists of 18 839 cancer survivors, defined using SNOMED CT codes and grouped into 20 cancer sites. Each cancer survivor is matched on age and sex to four non-cancer comparators (n=75 356). Cancer survivors with clinically diagnosed hypertension (n=8340) and ischaemic CVDs (n=2192) were separately matched on age and sex to four non-cancer comparators with hypertension and ischaemic CVDs; hypertension and cholesterol management was evaluated in these subgroups. Ischaemic CVDs refer to history of myocardial infarction, angina, peripheral artery disease, stroke, transient ischaemic attacks. CVD, cardiovascular disease; GP, general practitioner; SNOMED CT, Systematized Nomenclature of Medicine—Clinical Terms.

We analysed 18 839 cancer survivors and 75 356 matched controls (figure 1), with an average age of 64 years and 43% men (table 1). The sample was ethnically diverse, with the largest groups being White (48% cancer survivors, 42% in controls), Black (24% vs 22%) and Asian (21.7% vs 29.2%). Nearly 80% of participants were in the two most deprived IMD quintiles. The average time from cancer diagnosis was 8.2 (7.1) years. Cancer survivors had higher rates of hypertension (44% vs 41%) and CKD (18% vs 14%), while diabetes prevalence was similar (23% in both groups). CVDs were more prevalent in cancer survivors, particularly VTE (6% vs 3%), AF (7% vs 5%) and heart failure (4% vs 3%).

**Table 1:** Baseline characteristics of the cancer cases and age and sex matched controls

	Controls	Cancer cases
	N=75 356	N=18 839
Age, years	64.4 (15.0)	64.5 (15.1)
Sex, % male	32 104 (42.6%)	8026 (42.6%)
Average time from cancer diagnosis, years	*NA*	8.2 (7.1)
Ethnicity		
White	26 555 (41.9%)	7552 (48.3%)
Mixed	1792 (2.8%)	455 (2.9%)
Asian	18 528 (29.2%)	3389 (21.7%)
Black	14 016 (22.1%)	3713 (23.7%)
Other	2510 (4.0%)	538 (3.4%)
Index of multiple deprivation		
1st quintile	32 325 (42.9%)	7781 (41.3%)
2nd quintile	27 287 (36.2%)	6984 (37.1%)
3rd quintile	10 403 (13.8%)	2711 (14.4%)
4th quintile	4073 (5.4%)	1045 (5.5%)
5th quintile	1231 (1.6%)	310 (1.6%)
Non-smoker	45 825 (62.8%)	10 957 (58.9%)
Ex-smoker	17 115 (23.4%)	5335 (28.7%)
Current smoker	10 075 (13.8%)	2323 (12.5%)
Alcohol units/week	2.5 (7.0)	2.8 (7.2)
Alcohol misuse, % yes	1199 (1.6%)	317 (1.7%)
BMI, kg/m^2^	27.7 (6.0)	27.5 (6.1)
SBP, mm Hg	130.6 (13.5)	129.7 (13.4)
DBP, mm Hg	77.0 (8.5)	77.0 (8.5)
Diabetes	17 112 (22.7%)	4249 (22.6%)
Hypertension	31 084 (41.2%)	8340 (44.3%)
Chronic kidney disease	10 880 (14.4%)	3357 (17.8%)
Ischaemic heart disease	6626 (8.8%)	1747 (9.3%)
Stroke/TIA	2115 (2.8%)	598 (3.2%)
Heart failure	2566 (3.4%)	833 (4.4%)
Atrial fibrillation	3737 (5.0%)	1216 (6.5%)
Venous thromboembolism	2121 (2.8%)	1064 (5.6%)

Continuous variables are shown as mean (SD) or median (IQR), and categorical variables are as n (%).

BMI, body mass index; DBP, diastolic blood pressure; SBP, systolic blood pressure; TIA, transient ischaemic attack.

#### Cancer subgroups

**Figure 2:**
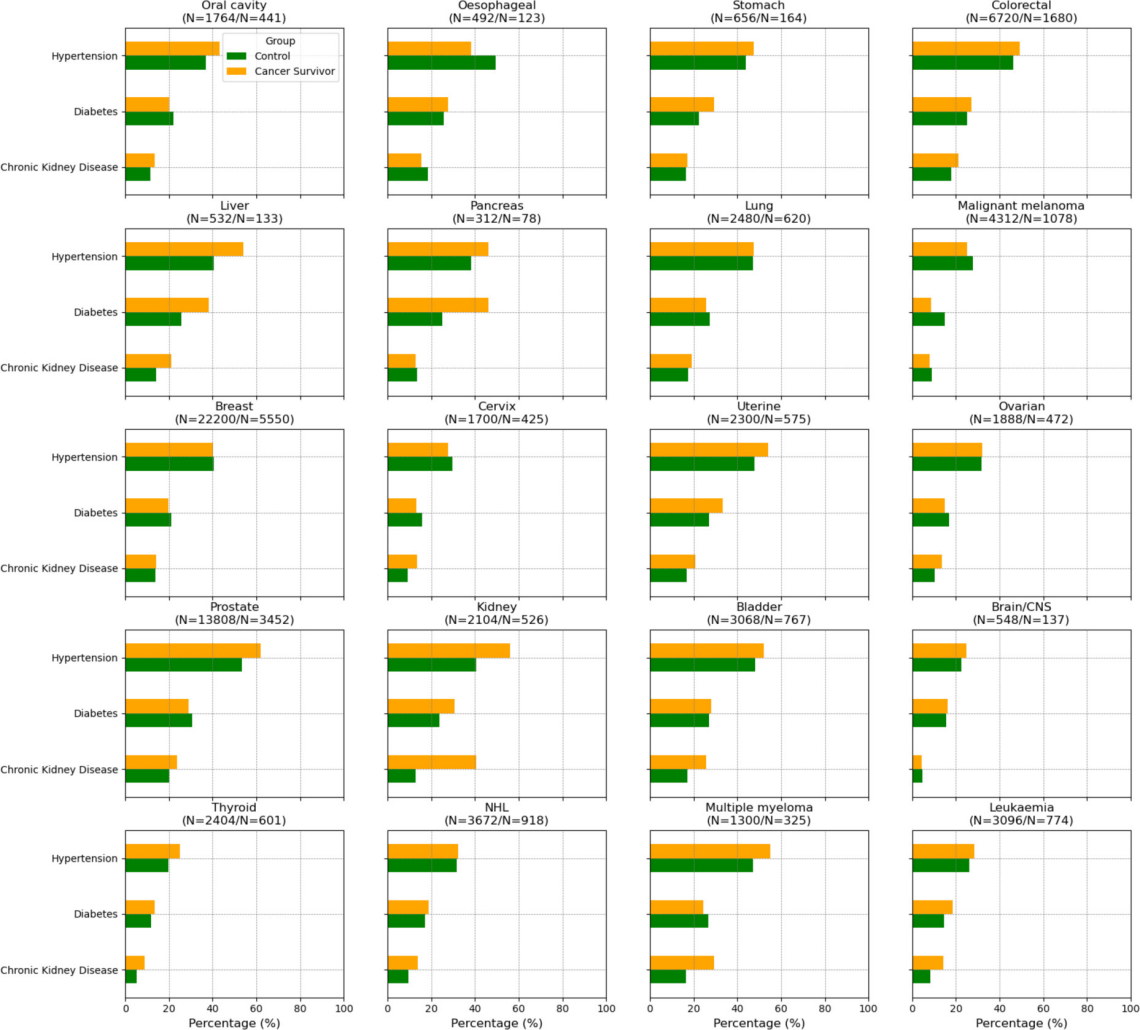
Prevalence of vascular risk factors in cancer survivors and age and sex matched controls. The figure represents the percentage of cancer survivors and matched controls with key vascular risk factors across different types of cancer. The brackets above each subplot title indicate the number of controls and the number of cancer cases on each plot (N=control/N=cancer survivors). The bars denote the percentage of vascular risk factors in cancer survivors (yellow bars) versus matched controls (green bars). NHL, non-Hodgkin's lymphoma.

The most common cancers were breast (29%), prostate (18%) and colorectal (9%) with haematological cancers accounting for 11%. Ethnic distribution varied across cancer types with White ethnicities dominant in malignant melanoma (90%), while Asian and Black groups were more common in liver (40%) and prostate (48%) cancers, respectively (online supplemental table 3A–E). Smoking was highest in lung (77%) and bladder (64%) cancers. CKD was most common in kidney (41%) and bladder (26%) cancers, and in multiple myeloma (30%). Hypertension was most common in those with prostate (62%), multiple myeloma (55%) and bladder (52%) cancers. Diabetes was highest in pancreatic (46%), liver (38%) and uterine (33%) cancers (figure 2).

### Associations with prevalent cardiovascular conditions

#### Vascular risk factors

**Figure 3:**
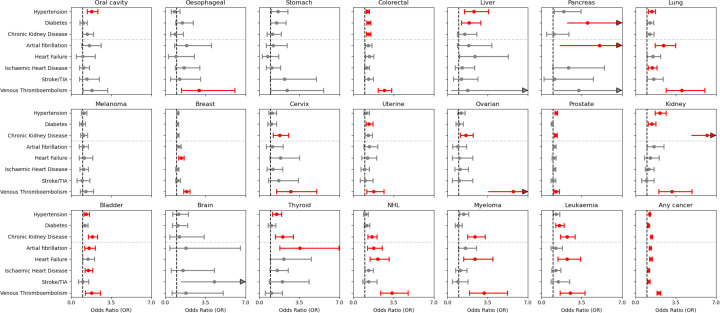
Association of past cancer exposure and vascular risk factors and cardiovascular diseases. Associations are reported from Firth regression models adjusted for age, sex, ethnicity, deprivation, smoking, body mass index, and alcohol intake. The red bars indicate statistically significant associations after multiple testing correction. Significant associations are shown in red colour. A grey horizontal dashed line separates vascular risk factors (hypertension, diabetes, chronic kidney disease) from cardiovascular diseases (atrial fibrillation, heart failure, ischaemic heart disease, stroke). NHL, non-Hodgkin's lymphoma; TIA, transient ischaemic attack.

Survivors of any cancer had significantly higher odds of hypertension (1.23 (1.18, 1.28)), diabetes (1.12 (1.07, 1.17)) and CKD (1.37 (1.31, 1.44)), independent of age, sex, ethnicity, deprivation, smoking, BMI and alcohol intake (figure 3, online supplemental table 4). The highest odds of CKD were observed in those with kidney cancer (6.20 (4.83, 7.99)), multiple myeloma (2.40 (1.76, 3.28)) and leukaemia (2.21 (1.66, 2.92)). Hypertension was most marked in those with kidney (2.10 (1.68, 2.64)), liver (2.28 (1.47, 3.57)) and oral cavity (1.23 (1.18, 1.28)) cancers. The odds of diabetes were highest in pancreatic cancer (4.01 (2.21, 7.39)), liver (1.87 (1.20, 2.90)) and leukaemia (1.55 (1.22, 1.96) (online supplemental table 4).

#### Cardiovascular diseases

After adjusting for standard risk factors, patients with a record of any cancer had significantly heightened odds of all prevalent CVDs compared with matched controls (figure 3, online supplemental table 4, online supplemental central illustration). The highest odds were for VTE (2.01 (1.86, 2.17)), with the strongest associations in ovarian (5.72 (3.54, 9.32)), lung (4.00 (2.66, 6.03)) and colorectal (2.68 (2.15, 3.32)) cancers. Haematological cancer also showed significantly heightened VTE odds: leukaemia: (2.49 (1.62, 3.79)), myeloma (3.17 (1.91, 5.23)) and non-Hodgkin's lymphoma (3.36 (2.36, 4.77)). Heightened odds of AF were observed in survivors of thyroid (3.54 (1.79, 6.93)), pancreas (5.05 (1.57, 17.27)) and lung (2.44 (1.71, 3.48)) cancers, with smaller effect in those with non-Hodgkin's lymphoma (1.77 (1.23, 2.51)) and bladder cancer (1.56 (1.17, 2.08)). Patients with breast cancer (1.38 (1.16, 1.64)), non-Hodgkin's lymphoma (2.12 (1.44, 3.09)) and leukaemia (2.22 (1.43, 3.40)) had significantly elevated odds of heart failure. All relationships remained robust in complete case analyses (online supplemental table 4).

#### Interaction analysis

Ethnicity and deprivation impact were tested using interaction term analyses in fully adjusted models. A significant interaction effect was found between ethnicity and cancer (FDR corrected p=0.018) for VTE, with the highest effect size in Asian ethnic groups (2.82 (2.31–3.43)) (online supplemental table 5). A significant association between hypertension and time since cancer diagnosis (FDR-corrected p=0.002) was also observed. We demonstrate a decline in odds of hypertension for each year after cancer diagnosis with the estimated probability of hypertension decreasing from 43% in those 1-year postdiagnosis to 40% after 10 years. No further significant interaction effects were found for other outcomes or cancer types.

### Hypertension management

The analysis included 8340 cancer survivors and 33 360 non-cancer controls with clinically diagnosed hypertension matched on age and sex. The average age was 72.1±11.3 years and 49% were men. Ethnic distributions and antihypertensive prescribing practices were similar between survivors and controls (table 2). Average blood pressure in survivors of any cancer was 135/78 mm Hg, compared with 136/78 mm Hg for controls, with suboptimal blood pressure control in 32% of survivors and 34% of controls. Cancer history was associated with significantly lower systolic and diastolic blood pressure (table 2). Cancer survivors were 8% more likely to meet age-specific NICE targets for blood pressure control with no significant subgroup differences (table 2). No significant interaction effects were found for ethnicity or deprivation.

**Table 2:** Hypertension management in cancer survivors and matched controls with clinically diagnosed hypertension

	Matched controls with hypertension (N=33 360)	Cancer survivors with hypertension (N=8340)
Age, years	71.9 (11.1)	72.1 (11.3)
Sex, % male	16 216 (48.6%)	4054 (48.6%)
Ethnicity		
White	10 057 (34.7%)	2942 (41.4%)
Mixed	801 (2.8%)	187 (2.6%)
Asian	9115 (31.5%)	1572 (22.1%)
Black	8067 (27.9%)	2215 (31.1%)
Other	911 (3.1%)	197 (2.8%)
SBP, mm Hg	136.1 (16.4)	135.1 (16.2)
DBP, mm Hg	77.9 (10.9)	77.6 (10.7)
ACEi/ARB	20 317 (60.9%)	4963 (59.5%)
CCB	19 304 (57.9%)	4781 (57.3%)
Thiazide	6443 (19.3%)	1522 (18.2%)
Other*	4556 (13.7%)	1212 (14.5%)
> 3 anti-hypertensives	833 (2.5%)	210 (2.5%)
Suboptimal BP control	10 958 (34.3%)	2604 (32.1%)
Association of cancer history with hypertension control indicators†		
Suboptimal BP control (OR (95% CI), p value)	0.92 (0.87, 0.97); p=0.001	
SBP (beta (95% CI), p value)	−0.89 (–1.28, –0.52); p=1.7×10^-5^	
DBP (beta (95% CI), p value)	−0.40 (–0.64, –0.16); p=0.00	

The table shows the characteristics of cancer survivors and controls with clinically diagnosed hypertension.

* Other includes beta blockers, spironolactone/potassium-sparing diuretics, alpha blockers and loop diuretics.

† Analysis is in a subset of patients with clinical hypertension, and results are an association of cancer history with (1) suboptimal blood pressure control, and clinic (2) systolic and (3) diastolic blood pressure measurements, compared with matched non-cancer controls adjusted for age, sex, ethnicity, deprivation, smoking, body mass index and alcohol intake.

ACEi, angiotensin-converting enzyme inhibitor; ARB, angiotensin II receptor blocker; BP, blood pressure; CCB, calcium channel blocker; DBP, diastolic blood pressure; NICE, National Institute for Health and Care Excellence; SBP, systolic blood pressure.

### Cholesterol management

The analysis included 2192 cancer survivors and 8768 non-cancer controls with clinically diagnosed ischaemic CVDs matched on age and sex (table 3). The average age was 75 years, with 61% men. Ethnic distributions were White (50% cancer survivors, 40% controls), Black (20% vs 16%) and Asian (25% vs 39%). Statin prescription rates were similar between cancer survivors (86.9%) and controls (88.4%). Of those with any cancer, 68.2% were prescribed suboptimal intensity statins compared with 67.2% in the control group. No significant differences were found between survivors and controls in any statin prescribing, statin intensity or serum cholesterol levels. There was a significant interaction effect between oral cancer history and ethnicity for serum HDL-C (FDR-adjusted p=1.3×10^−5^), after full covariate adjustment and multiple testing correction, with higher HDL-C levels in oral cancer survivors from White and “Other” ethnic backgrounds (online supplemental table 6). A significant interaction was also found between bladder cancer and ethnicity (FDR-adjusted p=0.005) for LDL-C, with survivors of bladder cancer from Black ethnicities having significantly higher mean serum LDL-C than controls (3.25 (1.10) vs 2.07 (0.90); p=0.005) (online supplemental table 7). No significant interaction effect was found for deprivation or time from cancer diagnosis in cholesterol control indicators across cancer groups.

**Table 3:** Cholesterol management in cancer survivors and controls with ischaemic cardiovascular diseases

	Matched controls with ischaemic CVDs (N=8768)	Cancer survivors with ischaemic CVDs (N=2192)
Age, years	75.0 (10.2)	75.3 (10.4)
Sex, % male	5336 (60.9%)	1334 (60.9%)
Ethnicity		
White	3005 (39.7%)	889 (49.5%)
Mixed	149 (2.0%)	44 (2.4%)
Asian	2943 (38.9%)	456 (25.4%)
Black	1171 (15.5%)	350 (19.5%)
Other	292 (3.9%)	57 (3.2%)
Total cholesterol, mmol/L	4.0 (1.1)	4.0 (1.1)
HDL, mmol/L	1.3 (0.3)	1.4 (0.4)
LDL, mmol/L	2.1 (0.9)	2.1 (0.9)
Any statin use	7754 (88.4%)	1904 (86.9%)
Suboptimal statin use	5889 (67.2%)	1496 (68.2%)
Association of cancer history with cholesterol control indicators*		
Any statin use (OR (95% CI), p value)	0.94 (0.81–1.08) p=0.368	
Suboptimal statin use (OR (95% CI), p value)	1.05 (0.95, 1.16), p=0.533	
Total cholesterol (beta (95% CI), p value)	0.02 (–0.03, 0.07), p=0.533	
HDL (beta (95% CI), p value)	0.00 (–0.01, 0.02), p=0.666	
LDL (beta (95% CI), p value)	0.02 (–0.02, 0.06), p=0.5333	

The table shows the characteristics of cancer survivors and controls with ischaemic cardiovascular diseases. Total cholesterol, HDL and LDL values are mean concentrations reported in mmol/L. Cholesterol control status (optimal vs suboptimal) is defined according to NICE guidelines with counts (N) and percentages of patient population in each category.

* Analysis is in a subset of patients with ischaemic cardiovascular diseases, and results are an association of cancer history with (1) any statin prescribing, (2) optimal statin prescribing and (3) serum lipids, compared with matched non-cancer controls adjusted for age, sex, ethnicity, deprivation, smoking, body mass index and alcohol intake.

CVD, cardiovascular disease; HDL, high-density lipoprotein; LDL, low-density lipoprotein; NICE, National Institute for Health and Care Excellence.

## Discussion

This study, conducted within a large, multiethnic and socioeconomically deprived population in East London, provides unique insights into the cardiovascular risk and management of adult cancer survivors. We identified significant associations between cancer survivorship and increased vascular risk factors, including CKD, diabetes and hypertension. Specifically, cancer survivors had 23% higher odds of hypertension, 12% higher odds of diabetes and 37% higher odds of CKD, compared with controls, independent of demographics and lifestyle factors. These risks were most pronounced in specific cancer subtypes, with hypertension being more prevalent in survivors of kidney and liver cancers, CKD in survivors of kidney cancer, multiple myeloma and leukaemia and diabetes most commonly observed in pancreatic cancer. These findings align with the existing literature, which underscores the well-established link between cancer survivorship and increased cardiovascular risk.^17 18^ We additionally demonstrate the disproportionate burden of cardiovascular risk within a diverse population living in disadvantaged communities, who are often under-represented in research.

Furthermore, our study showed a significantly higher risk of CVDs among cancer survivors, particularly VTE, AF and heart failure. VTE presented the strongest association, with cancer survivors exhibiting more than two times the risk of non-cancer controls. Several factors may contribute to venous thrombosis, including procoagulant factors produced by the tumour cells and chronic inflammation.^19^ However, the risk of VTE varied by primary cancer type, with markedly elevated VTE risk associated with ovarian, lung, colorectal and haematological cancers, suggesting the involvement of additional cancer-specific mechanisms. For instance, the six-fold increased risk in ovarian cancer survivors may be driven by factors such as tumour burden and ascites accumulation, which contribute to venous stasis and a prothrombotic milieu.^20^ Similarly, the fourfold increased VTE risk observed among lung survivors may be attributed, in part, to direct mechanical tumour effects on surrounding vasculature and smoking.^21^ Ethnic disparities were also notable, with Asian survivors showing a near threefold increased risk of VTE. This observation underscores the importance of considering both ethnicity and cancer subtype in clinical decision-making.

One of the key strengths of this study is the evaluation of adherence to cardiovascular risk management guidelines within a high-risk population. Cancer survivors face a higher risk of hypertension, which in turn increases the risk of subsequent CVD over the trajectory of survivorship.^22^ Despite the significant impact on cardiovascular health and mortality, hypertension is often underdiagnosed or treated inadequately in cancer survivors.^23 24^ While hypertension control in the study region was well managed in comparison to national averages,^12^ around 32% of cancer survivors did not achieve age-specific blood pressure targets, indicating significant room for improvement. Continuing blood pressure monitoring remains essential for cancer survivors to ensure timely detection and management of hypertension.

Overall, statin prescription rates and lipid management were similar between cancer survivors and non-cancer controls. While no significant interaction effects were found for deprivation or time since cancer diagnosis, stratified subgroup analyses showed distinct ethnic disparities in lipid profiles. For instance, survivors of oral cancer from White and Other ethnic backgrounds had significantly higher mean HDL cholesterol levels, whereas Black bladder cancer survivors exhibited higher levels of mean LDL-C compared with matched controls. Existing literature supports this observation, with previous studies highlighting that Black individuals are less likely to receive lipid measurement or diagnosis and treatment of dyslipidaemia.^25 26^ These findings highlight the need for heightened awareness of increased cardiovascular risk among diverse ethnic groups and emphasise that ethnicity should be a consideration in assessing cardiovascular risks and management strategies.

### Limitations

Several limitations should be considered when interpreting our findings. The absence of detailed data on cancer stages, disease burden and cancer treatments limits our understanding of how these factors influence long-term cardiovascular outcomes. Medication was defined through prescription records, which may not reflect actual usage. Furthermore, interval changes in serum lipids could not be captured. Data were missing in 20% of covariates, which was addressed using multiple imputations alongside complete case analysis, however, some residual bias may remain. Furthermore, while statistical adjustments were applied, factors such as deprivation, smoking and BMI are proximal risk factors that may influence both cancer and cardiovascular outcomes. Additionally, this study focuses on retrospective data and does not account for survival bias or allow for conclusions regarding temporal relationships between cancer and cardiovascular outcomes.

## Conclusion

Our study provides novel insights into the increased cardiovascular risks faced by cancer survivors, highlighting significant variations by cancer type and ethnicity. Despite the relatively comparable or slightly better control of major cardiovascular risk factors such as blood pressure and lipids, one in three cancer survivors did not achieve guideline-recommended blood pressure targets, underscoring a major treatment gap. Moreover, disparities were identified within ethnic groups, with Asian survivors at higher risk of VTE and Black survivors showing elevated LDL cholesterol levels, suggesting further inequalities in risk and management. Greater awareness of these risks and optimised management strategies are crucial to improving outcomes for cancer survivors, particularly within ethnic minority groups. Future research incorporating detailed cancer treatment data, including the use of cardiotoxic therapies, is essential to better understand the mechanisms underlying the heightened cardiovascular risk observed in cancer survivors.

## Supplementary material

10.1136/heartjnl-2024-325309 online supplemental file 1

10.1136/heartjnl-2024-325309 online supplemental file 2

## Data Availability

All data relevant to the study are included in the article or uploaded as supplementary information.
