# Supplementary material for: Cardiovascular disease burden and risk factor management in cancer survivors: insights into a multiethnic, socioeconomically deprived urban population
Source: Heart. 2025 Mar 13;112(1):e325309. doi: 10.1136/heartjnl-2024-325309 (PMC12703254; doi:10.1136/heartjnl-2024-325309)
Supplement: online supplemental file 1 [file heartjnl-112-1-s001.docx]

**Cardiovascular Disease Burden and Risk Factor Control among Cancer Survivors in an Urban Primary Care Setting**

Supplemental Material

[Supplementary Table 1: SNOMED CT code list for cancer status 2](#_Toc174370342)

[Supplementary Table 2: SNOMED CT codes defining prevalent cardiovascular diseases 25](#_Toc174370343)

[and major vascular risk factors 25](#_Toc174370344)

[Supplementary Table 3A. Granular baseline characteristics 48](#_Toc174370345)

[Supplementary Table 3B. Granular baseline characteristics 49](#_Toc174370346)

[Supplementary Table 3C. Granular baseline characteristics 50](#_Toc174370347)

[Supplementary Table 3D. Granular baseline characteristics 51](#_Toc174370348)

[Supplementary Table 3E. Granular baseline characteristics 52](#_Toc174370349)

[Supplementary Table 4. Association between cancer survivorship and cardiovascular disease burden 53](#_Toc174370350)

[Supplementary Table 5. Association of cancer with prevalent venous thromboembolism stratified by ethnicity 57](#_Toc174370351)

[Supplementary Table 6. Mean serum serum HDL-C levels in survivors of oral cancer history compared to matched controls stratified by ethnicity 58](#_Toc174370352)

[Supplementary Table 7. Mean serum serum LDL-C levels in survivors of bladder cancer history compared to matched controls stratified by ethnicity 59](#_Toc174370353)

# **Supplementary Table 1: SNOMED CT code list for cancer status**

| **Description** | **Cancer type** | **SCTID** |
| --- | --- | --- |
| Multiple myeloma (disorder) | myeloma | 109989006 |
| Immunoglobulin A myeloma (disorder) | myeloma | 285420006 |
| Immunoglobulin G myeloma (disorder) | myeloma | 285421005 |
| Plasma cell myeloma (morphologic abnormality) | myeloma | 1162576007 |
| Myeloma cell (cell) | myeloma | 64446007 |
| Myeloma kidney (disorder) | myeloma | 32278006 |
| Plasmacytoma (morphologic abnormality) | myeloma | 1187107005 |
| Indolent multiple myeloma (disorder) | myeloma | 441313008 |
| Light chain myeloma (disorder) | myeloma | 277579002 |
| Asymptomatic multiple myeloma (disorder) | myeloma | 440422002 |
| Non-secretory myeloma (disorder) | myeloma | 277580004 |
| Osteosclerotic myeloma (disorder) | myeloma | 425657001 |
| Immunoglobulin D myeloma (disorder) | myeloma | 285422003 |
| Kappa light chain myeloma (disorder) | myeloma | 414553000 |
| Bone marrow: myeloma cells (finding) | myeloma | 167934009 |
| Lambda light chain myeloma (disorder) | myeloma | 313427003 |
| Smoldering myeloma (disorder) | myeloma | 413587002 |
| History of multiple myeloma (situation) | myeloma | 404684003 |
| Multiple myeloma in remission (disorder) | myeloma | 94704006 |
| Myeloma-associated amyloidosis (disorder) | myeloma | 209962000 |
| Plasma cell myeloma/plasmacytoma (disorder) | myeloma | 415110002 |
| Plasma cell myeloma/plasmacytoma (morphologic abnormality) | myeloma | 399456004 |
| X-ray skeletal survey for multiple myeloma (procedure) | myeloma | 429646003 |
| Neuropathy due to multiple myeloma (disorder) | myeloma | 230586003 |
| Osteoporosis co-occurrent and due to multiple myeloma (disorder) | myeloma | 739301006 |
| Amyloid light chain amyloidosis due to multiple myeloma (disorder) | myeloma | 402453006 |
| Light chain nephropathy due to multiple myeloma (disorder) | myeloma | 127911000119105 |
| Immunoglobulin G deficiency due to multiple myeloma (disorder) | myeloma | 16894711000119103 |
| Hypogammaglobulinemia due to multiple myeloma (disorder) | myeloma | 16318001000119107 |
| Leukemia (morphologic abnormality) | leukaemia | 1162768007 |
| Leukemia, disease (disorder) | leukaemia | 93143009 |
| Compound leukemias (disorder) | leukaemia | 190030009 |
| Acute leukemia (morphologic abnormality) | leukaemia | 24072005 |
| Chronic leukemia, disease (disorder) | leukaemia | 92812005 |
| Myeloid leukemia (disorder) | leukaemia | 188732008 |
| Chronic leukemia (morphologic abnormality) | leukaemia | 2557004 |
| Subacute leukemia (disorder) | leukaemia | 302855005 |
| Lymphoid leukemia (morphologic abnormality) | leukaemia | 1172709000 |
| Lymphoid leukemia (disorder) | leukaemia | 188725004 |
| Suspected leukemia (situation) | leukaemia | 315262009 |
| Mast cell leukemia (disorder) | leukaemia | 110002002 |
| Monocytic leukemia (morphologic abnormality) | leukaemia | 52168008 |
| Mast cell leukemia (morphologic abnormality) | leukaemia | 128924002 |
| B-cell acute lymphoblastic leukemia (disorder) | leukaemia | 277571004 |
| Monocytic leukemia (disorder) | leukaemia | 188744006 |
| Meningeal leukemia (disorder) | leukaemia | 722795004 |
| Hairy cell leukemia (morphologic abnormality) | leukaemia | 54087003 |
| Basophilic leukemia (disorder) | leukaemia | 307592006 |
| Acute basophilic leukemia (morphologic abnormality) | leukaemia | 69077002 |
| Aleukemic leukemia (disorder) | leukaemia | 302856006 |
| History of leukemia (situation) | leukaemia | 161436008 |
| Myeloid leukemia (morphologic abnormality | leukaemia | 1163439000 |
| Plasma cell leukemia, morphology (morphologic abnormality) | leukaemia | 128922003 |
| Myeloid leukemia (morphologic abnormality) | leukaemia | 1163439000 |
| Subacute myeloid leukemia (disorder) | leukaemia | 188736006 |
| Plasma cell leukemia, disease (disorder) | leukaemia | 95210003 |
| Acute monocytic leukemia, morphology (morphologic abnormality) | leukaemia | 22331004 |
| Adult T-cell leukemia/lymphoma (morphologic abnormality) | leukaemia | 77430005 |
| Burkitt cell leukemia (morphologic abnormality) | leukaemia | 22197008 |
| Eosinophilic leukemia (morphologic abnormality) | leukaemia | 190055003 |
| Megakaryocytic leukemia (disorder) | leukaemia | 188754005 |
| Adult T-cell leukemia/lymphoma (disorder) | leukaemia | 110007008 |
| Neutrophilic leukemia (disorder) | leukaemia | 307617006 |
| Acute myeloid leukemia, disease (disorder) | leukaemia | 91861009 |
| Acute myeloid leukemia (morphologic abnormality) | leukaemia | 1162928000 |
| Precursor cell lymphoblastic leukemia (morphologic abnormality) | leukaemia | 128822004 |
| Prolymphocytic leukemia (morphologic abnormality) | leukaemia | 128923008 |
| Acute megakaryoblastic leukemia, morphology (morphologic abnormality) | leukaemia | 52220008 |
| Prolymphocytic leukemia (disorder) | leukaemia | 110006004 |
| Acute biphenotypic leukemia (morphologic abnormality) | leukaemia | 128818009 |
| Myelomonocytic leukemia (morphologic abnormality) | leukaemia | 1163590002 |
| Acute lymphoid leukemia, disease (disorder) | leukaemia | 91857003 |
| Myelomonocytic leukemia (disorder) | leukaemia | 188768003 |
| Acute monocytic leukemia, FAB M5b (disorder) | leukaemia | 413441006 |
| Chronic myeloid leukemia, disease (disorder) | leukaemia | 92818009 |
| Chronic myeloid leukemia (morphologic abnormality) | leukaemia | 1162588009 |
| Acute monocytic leukemia, morphology (morphologic abnormality) | leukaemia | 22331004 |
| Acute myeloid leukemia, M6 type (morphologic abnormality) | leukaemia | 14317002 |
| History of acute leukemia (situation) | leukaemia | 400347211000119102 |
| B-cell chronic lymphocytic leukemia/small lymphocytic lymphoma (morphologic abnormality) | leukaemia | 51092000 |
| Hairy cell leukemia (disorder) | leukaemia | 118613001 |
| Acute monoblastic leukemia (disorder) | leukaemia | 277601005 |
| Subacute lymphoid leukemia (disorder) | leukaemia | 188726003 |
| Subacute lymphoid leukemia (morphologic abnormality) | leukaemia | 33560006 |
| Chronic monocytic leukemia (morphologic abnormality) | leukaemia | 92816008 |
| Gingivitis due to leukemia (disorder) | leukaemia | 705127009 |
| Chronic monocytic leukemia (disorder) | leukaemia | 188745007 |
| Aggressive natural killer-cell leukemia (morphologic abnormality) | leukaemia | 128833001 |
| Subacute monocytic leukemia (morphologic abnormality) | leukaemia | 12834001 |
| Acute myeloid leukemia, minimal differentiation (morphologic abnormality) | leukaemia | 103689001 |
| Hairy cell leukemia variant (morphologic abnormality) | leukaemia | 1187193000 |
| Acute eosinophilic leukemia (morphologic abnormality) | leukaemia | 1162993004 |
| History of myeloid leukemia (situation) | leukaemia | 152861000119104 |
| Acute leukemia in remission (disorder) | leukaemia | 91854005 |
| Aleukemic myeloid leukemia (disorder) | leukaemia | 188741003 |
| Acute eosinophilic leukemia (disorder) | leukaemia | 277604002 |
| Hairy cell leukemia variant (disorder) | leukaemia | 277568007 |
| Acute biphenotypic leukemia (disorder) | leukaemia | 278453007 |
| Subacute monocytic leukemia (disorder) | leukaemia | 188746008 |
| Acute myeloid leukemia, 11q23 abnormalities (morphologic abnormality) | leukaemia | 128829008 |
| Mixed phenotype acute leukemia (morphologic abnormality) | leukaemia | 450913003 |
| Aleukemic lymphoid leukemia (morphologic abnormality) | leukaemia | 12219003 |
| Atypical hairy cell leukemia (disorder) | leukaemia | 307341004 |
| Aleukemic lymphoid leukemia (disorder) | leukaemia | 188728002 |
| History of lymphoid leukemia (situation) | leukaemia | 473058009 |
| Acute promyelocytic leukemia, t(15;17)(q22;q11-12) (morphologic abnormality) | leukaemia | 28950004 |
| Hairy cell leukemia of spleen (disorder) | leukaemia | 93151007 |
| Aleukemic monocytic leukemia (disorder) | leukaemia | 188748009 |
| Chronic neutrophilic leukemia (morphologic abnormality) | leukaemia | 128834007 |
| Aleukemic mast cell leukemia (disorder) | leukaemia | 770402000 |
| Chronic leukemia in remission (disorder) | leukaemia | 92811003 |
| History of monocytic leukemia (situation) | leukaemia | 427920005 |
| Chronic eosinophilic leukemia (disorder) | leukaemia | 188733003 |
| Chronic neutrophilic leukemia (disorder) | leukaemia | 188734009 |
| Chronic eosinophilic leukemia (morphologic abnormality) | leukaemia | 413836008 |
| Myeloid leukemia in remission (disorder) | leukaemia | 94716000 |
| Aleukemic monocytic leukemia (morphologic abnormality) | leukaemia | 22695001 |
| Acute myelomonocytic leukemia (morphologic abnormality) | leukaemia | 30962008 |
| Prolymphocytic leukemia, T-cell type (morphologic abnormality) | leukaemia | 128821006 |
| Hairy cell leukemia (disorder) | leukaemia | 118613001 |
| Mixed phenotype acute leukemia (disorder) | leukaemia | 1187123005 |
| Lymphoid leukemia in remission (disorder) | leukaemia | 93169003 |
| Relapsing acute myeloid leukemia (disorder) | leukaemia | 12281000132104 |
| T-cell prolymphocytic leukemia (disorder) | leukaemia | 277567002 |
| B-cell prolymphocytic leukemia (disorder) | leukaemia | 277619001 |
| Acute megakaryoblastic leukemia (disorder) | leukaemia | 277602003 |
| Chronic myelomonocytic leukemia (disorder) | leukaemia | 127225006 |
| Acute lymphoid leukemia relapse (disorder) | leukaemia | 12301000132103 |
| Chronic myelomonocytic leukemia (morphologic abnormality) | leukaemia | 128831004 |
| Subacute myelomonocytic leukemia (disorder) | leukaemia | 188770007 |
| Aleukemic leukemia in remission (disorder) | leukaemia | 426217000 |
| Inherited acute myeloid leukemia (disorder) | leukaemia | 764940002 |
| Philadelphia chromosome-positive acute lymphoblastic leukemia (disorder) | leukaemia | 425688002 |
| Juvenile myelomonocytic leukemia (disorder) | leukaemia | 445227008 |
| Philadelphia chromosome-positive acute lymphoblastic leukemia (morphologic abnormality) | leukaemia | 426955004 |
| T lymphoblastic leukemia/lymphoma (morphologic abnormality) | leukaemia | 703821009 |
| Acute promyelocytic leukemia, FAB M3 (disorder) | leukaemia | 110004001 |
| Refractory acute myeloid leukemia (disorder) | leukaemia | 12291000132102 |
| Juvenile chronic myeloid leukemia (disorder) | leukaemia | 277587001 |
| Atypical chronic myeloid leukemia (disorder) | leukaemia | 277589003 |
| History of acute myeloid leukemia (situation) | leukaemia | 79081000119100 |
| Mast cell leukemia affecting skin (disorder) | leukaemia | 404172001 |
| Plasma cell leukemia in remission (disorder) | leukaemia | 95209008 |
| B lymphoblastic leukemia lymphoma (morphologic abnormality) | leukaemia | 1162660006 |
| History of acute lymphoid leukemia (situation) | leukaemia | 676112841000119101 |
| Relapsing chronic myeloid leukemia (disorder) | leukaemia | 415287001 |
| Chronic lymphoid leukemia, disease (disorder) | leukaemia | 92814006 |
| Natural killer-lymphoblastic leukemia/lymphoma (disorder) | leukaemia | 1258932003 |
| Myeloid leukemia co-occurrent with Down syndrome (disorder) | leukaemia | 724644005 |
| Acute myelomonocytic leukemia, FAB M4 (disorder) | leukaemia | 110005000 |
| Natural killer-lymphoblastic leukemia/lymphoma (morphologic abnormality) | leukaemia | 783414002 |
| Refractory acute lymphoid leukemia (disorder) | leukaemia | 12311000132101 |
| Large granular lymphocytic leukemia (disorder) | leukaemia | 277569004 |
| T-cell chronic lymphocytic leukemia (disorder) | leukaemia | 277545003 |
| Common acute lymphoblastic leukemia (disorder) | leukaemia | 277573001 |
| Acute leukemia of ambiguous lineage (morphologic abnormality) | leukaemia | 397345009 |
| Acute leukemia of ambiguous lineage (disorder) | leukaemia | 721308005 |
| T-cell acute lymphoblastic leukemia (disorder) | leukaemia | 277575008 |
| Acute myeloid leukemia in remission (disorder) | leukaemia | 91860005 |
| Megakaryocytic leukemia in remission (disorder) | leukaemia | 94148006 |
| Prolymphocytic leukemia, B-cell type (morphologic abnormality) | leukaemia | 128820007 |
| Acute monocytic/monoblastic leukemia (disorder) | leukaemia | 413442004 |
| Acute myeloid leukemia with BCR-ABL1 (disorder) | leukaemia | 1237368006 |
| Acute lymphoid leukemia in remission (disorder) | leukaemia | 91856007 |
| Hypergranular promyelocytic leukemia (disorder) | leukaemia | 278189009 |
| Acute myeloid leukemia with BCR-ABL1 (morphologic abnormality) | leukaemia | 783017000 |
| Chronic myeloid leukemia in remission (disorder) | leukaemia | 92817004 |
| Acute monocytic leukemia in remission (disorder) | leukaemia | 91858008 |
| Adult T-cell leukemia/lymphoma of skin (disorder) | leukaemia | 721762007 |
| Chronic lymphoid leukemia in remission (disorder) | leukaemia | 92813000 |
| Acute myeloid leukemia with abnormal marrow eosinophils and inv(16)(p13q22) or t(16;16)(p13;q22) (morphologic abnormality) | leukaemia | 103688009 |
| Null cell acute lymphoblastic leukemia (disorder) | leukaemia | 277574007 |
| Periodontitis co-occurrent with leukemia (disorder) | leukaemia | 709471005 |
| Acute myeloid leukemia with maturation (morphologic abnormality) | leukaemia | 103691009 |
| Blastic phase chronic myeloid leukemia (disorder) | leukaemia | 413656006 |
| Subacute myeloid leukemia in remission (disorder) | leukaemia | 425749006 |
| Chronic phase chronic myeloid leukemia (disorder) | leukaemia | 413847001 |
| Chronic myelogenous leukemia, BCR/ABL positive (morphologic abnormality) | leukaemia | 128825002 |
| Subacute lymphoid leukemia in remission (disorder) | leukaemia | 426370008 |
| Acute promyelocytic leukemia, FAB M3 (disorder | leukaemia | 110004001 |
| History of chronic lymphocytic leukemia (situation) | leukaemia | 63581000119104 |
| Precursor B-cell lymphoblastic leukemia (morphologic abnormality) | leukaemia | 128823009 |
| Precursor B-cell acute lymphoblastic leukemia (disorder) | leukaemia | 277572006 |
| Precursor T-cell lymphoblastic leukemia (morphologic abnormality) | leukaemia | 128824003 |
| Acute monoblastic leukemia in remission (disorder) | leukaemia | 698646006 |
| Acute myeloid leukemia with mutated NPM1 (morphologic abnormality) | leukaemia | 703820005 |
| Smoldering chronic lymphocytic leukemia (disorder) | leukaemia | 430338009 |
| Juvenile myelomonocytic leukemia (morphologic abnormality) | leukaemia | 128832006 |
| Acute myeloid leukemia in complete remission (disorder) | leukaemia | 1162531007 |
| Mixed phenotype acute leukemia with myeloid and B-cell lymphoid phenotypes (morphologic abnormality) | leukaemia | 450916006 |
| Mixed phenotype acute leukemia with myeloid and T-cell lymphoid phenotypes (morphologic abnormality) | leukaemia | 450917002 |
| Acute monoblastic and monocytic leukemia (morphologic abnormality) | leukaemia | 703818007 |
| Acute myeloid leukemia with FMS-like tyrosine kinase-3 mutation (disorder) | leukaemia | 734522002 |
| Acute myeloid leukemia with mutated RUNX1 (morphologic abnormality) | leukaemia | 783263001 |
| Aleukemic lymphoid leukemia in remission (disorder) | leukaemia | 426248008 |
| Acute myeloid leukemia, t(8;21) (q22;q22) (morphologic abnormality) | leukaemia | 128828000 |
| Acute myeloid leukemia without maturation (morphologic abnormality) | leukaemia | 103690005 |
| Acute myeloid leukemia with FMS-like tyrosine kinase-3 mutation (morphologic abnormality) | leukaemia | 734524001 |
| T-cell large granular lymphocytic leukemia (disorder) | leukaemia | 699818003 |
| Aggressive natural killer-cell leukemia involving skin (disorder) | leukaemia | 404136008 |
| T-cell large granular lymphocytic leukemia (morphologic abnormality) | leukaemia | 128819001 |
| Accelerated phase chronic myeloid leukemia (disorder) | leukaemia | 413389003 |
| Core binding factor acute myeloid leukemia (disorder) | leukaemia | 702446006 |
| Common acute lymphoblastic leukemia (disorder) | leukaemia | 277573001 |
| Adult T-cell leukemia/lymphoma in remission (disorder) | leukaemia | 898197001 |
| B-cell chronic lymphocytic leukemia variant (disorder) | leukaemia | 277474005 |
| T-cell prolymphocytic leukemia in remission (disorder) | leukaemia | 898198006 |
| B-cell prolymphocytic leukemia in remission (disorder) | leukaemia | 788874003 |
| Acute myeloid leukemia with normal karyotype (disorder) | leukaemia | 703387000 |
| Juvenile myelomonocytic leukemia in remission (disorder) | leukaemia | 788972003 |
| Acute myeloid leukemia without maturation, FAB M1 (disorder) | leukaemia | 359640008 |
| B lymphoblastic leukemia lymphoma with intrachromosomal amplification of chromosome 21 (morphologic abnormality) | leukaemia | 785825000 |
| Clinical stage B chronic lymphocytic leukaemia (disorder) | leukaemia | 863761000000109 |
| Clinical stage A chronic lymphocytic leukaemia (disorder) | leukaemia | 863741000000108 |
| Acute myeloid leukemia with 11q23 abnormality (disorder) | leukaemia | 1157157006 |
| Clinical stage C chronic lymphocytic leukaemia (disorder) | leukaemia | 863781000000100 |
| Acute myeloid leukemia with maturation, FAB M2 (disorder) | leukaemia | 359648001 |
| Myeloid leukemia associated with Down Syndrome (morphologic abnormality) | leukaemia | 450935006 |
| B-lymphoblastic leukemia lymphoma BCR-ABL1-like (morphologic abnormality) | leukaemia | 783744003 |
| Chronic myeloid leukemia in myeloid blast crisis (disorder) | leukaemia | 413843002 |
| T-cell acute lymphoblastic leukemia in remission (disorder) | leukaemia | 427642009 |
| Acute myeloid leukemia with nucleophosmin 1 somatic mutation (disorder) | leukaemia | 763309005 |
| Chronic myeloid leukemia in lymphoid blast crisis (disorder) | leukaemia | 413842007 |
| Leukemic infiltration of skin in myeloid leukemia (disorder) | leukaemia | 404151004 |
| Acute myeloid leukemia with multilineage dysplasia (morphologic abnormality) | leukaemia | 128827005 |
| Acute promyelocytic leukemia, FAB M3, in remission (disorder) | leukaemia | 425869007 |
| Acute myeloid leukemia with myelodysplasia-related changes (disorder) | leukaemia | 445448008 |
| Atypical chronic myeloid leukemia, BCR/ABL negative (morphologic abnormality) | leukaemia | 128826001 |
| Acute promyelocytic leukemia - hypogranular variant (disorder) | leukaemia | 285769009 |
| Acute myelomonocytic leukemia, FAB M4, in remission (disorder) | leukaemia | 427658007 |
| Acute myeloid leukemia, minimal differentiation, FAB M0 (disorder) | leukaemia | 359631009 |
| B lymphoblastic leukemia lymphoma with hypodiploidy (disorder) | leukaemia | 838344002 |
| Acute myeloid leukemia with CCAAT/enhancer binding protein alpha somatic mutation (disorder) | leukaemia | 764855007 |
| Acute myelomonocytic leukemia - eosinophilic variant (disorder) | leukaemia | 285839005 |
| B lymphoblastic leukemia lymphoma with hyperdiploidy (morphologic abnormality) | leukaemia | 450953000 |
| Leukemic infiltration of skin in monocytic leukemia (disorder) | leukaemia | 404154007 |
| Chronic lymphocytic prolymphocytic leukemia syndrome (disorder) | leukaemia | 277549009 |
| B lymphoblastic leukemia lymphoma with hyperdiploidy (disorder) | leukaemia | 838346000 |
| Precursor B-cell acute lymphoblastic leukemia in remission (disorder) | leukaemia | 425941003 |
| Chronic lymphocytic leukemia genetic mutation variant (disorder) | leukaemia | 725437002 |
| Leukemic infiltration of skin in hairy-cell leukemia (disorder) | leukaemia | 404139001 |
| Acute lymphoblastic leukemia, transitional pre-B-cell (disorder) | leukaemia | 371012000 |
| B-cell leukemia lymphoma 2 gene rearrangement analysis (procedure) | leukaemia | 432824002 |
| Acute myeloid leukemia with t(6;9)(p23;q34); DEK-NUP214 (morphologic abnormality) | leukaemia | 450928003 |
| Acute myeloid leukemia with t(9:11)(p22;q23); MLLT3-MLL (disorder) | leukaemia | 444911000 |
| Acute myeloid leukemia with t(9;11)(p22;q23); MLLT3-MLL (morphologic abnormality) | leukaemia | 1157158001 |
| Acute myeloid leukemia with t(6;9)(p23;q34) translocation (disorder) | leukaemia | 733598001 |
| Acute myeloid leukemia with recurrent genetic abnormality (morphologic abnormality) | leukaemia | 397340004 |
| Acute myeloid leukemia with t(8;16)(p11;p13) translocation (disorder) | leukaemia | 725390002 |
| Acute myeloid leukemia with t(8;21)(q22;q22) RUNX1-RUNX1T1 (disorder) | leukaemia | 1148906001 |
| Acute myeloid leukemia with t(8;16)(p11;p13) translocation (morphologic abnormality) | leukaemia | 725391003 |
| Acute myeloid leukemia due to recurrent genetic abnormality (disorder) | leukaemia | 721305008 |
| Acute myeloid leukemia with maturation, FAB M2, in remission (disorder) | leukaemia | 426124006 |
| Philadelphia chromosome positive chronic myelogenous leukemia (disorder) | leukaemia | 449108003 |
| Philadelphia chromosome negative chronic myelogenous leukemia (disorder) | leukaemia | 449386007 |
| Megakaryoblastic acute myeloid leukemia with t(1;22)(p13;q13) (disorder) | leukaemia | 763796007 |
| Mixed phenotype acute leukemia with t(v;11q23); MLL rearranged (morphologic abnormality) | leukaemia | 450915005 |
| Mixed phenotype acute leukemia with T-cell and myeloid lineage (disorder) | leukaemia | 1153379006 |
| Leukemic infiltration of skin (T-cell lymphoblastic leukemia) (disorder) | leukaemia | 404124002 |
| B-cell chronic lymphocytic leukemia/small lymphocytic lymphoma (morphologic abnormality | leukaemia | 51092000 |
| Leukemic infiltration of skin (T-cell prolymphocytic leukemia) (disorder) | leukaemia | 404123008 |
| B lymphoblastic leukemia lymphoma with t(5;14)(q31;q32); IL3-IGH (morphologic abnormality) | leukaemia | 450955007 |
| Mixed phenotype acute leukemia with t(9;22)(q34;q11.2); BCR-ABL1 (morphologic abnormality) | leukaemia | 450914009 |
| B lymphoblastic leukemia lymphoma with t(5;14)(q31;q32); IL3-IGH (disorder) | leukaemia | 838340006 |
| Non-chronic lymphocytic leukemia monoclonal B-cell lymphocytosis (disorder) | leukaemia | 783211008 |
| Therapy-related acute myeloid leukemia and myelodysplastic syndrome (morphologic abnormality) | leukaemia | 128830003 |
| Monoclonal B-cell lymphocytosis chronic lymphocytic leukemia-type (morphologic abnormality) | leukaemia | 789739001 |
| B lymphoblastic leukemia lymphoma with t(v;11q23); MLL rearranged (disorder) | leukaemia | 838341005 |
| Monoclonal B-cell lymphocytosis chronic lymphocytic leukemia-type (disorder) | leukaemia | 786855003 |
| B lymphoblastic leukemia lymphoma with t(v;11q23); MLL rearranged (morphologic abnormality) | leukaemia | 450951003 |
| Mixed phenotype acute leukemia with t(9;22) (q34;q11.2); BCR-ABL1 (disorder) | leukaemia | 1153377008 |
| Noonan syndrome-like disorder with juvenile myelomonocytic leukemia (disorder) | leukaemia | 783143001 |
| Therapy related acute myeloid leukemia and myelodysplastic syndrome (disorder) | leukaemia | 721306009 |
| B lymphoblastic leukemia lymphoma with t(9;22)(q34;q11.2); BCR-ABL1 (morphologic abnormality) | leukaemia | 450950002 |
| B lymphoblastic leukemia lymphoma with hypodiploidy (Hypodiploid ALL) (morphologic abnormality) | leukaemia | 450954006 |
| Monoclonal B-cell lymphocytosis non-chronic lymphocytic leukemia type (morphologic abnormality) | leukaemia | 789743002 |
| B lymphoblastic leukemia lymphoma with t(9:22) (q34;q11.2); BCR-ABL 1 (disorder) | leukaemia | 723889003 |
| Acute myeloid leukemia and myelodysplastic syndrome related to radiation (disorder) | leukaemia | 766048008 |
| Mixed phenotype acute leukemia with myeloid and B-cell lymphoid phenotypes (disorder) | leukaemia | 1153381008 |
| Acute myeloid leukemia (megakaryoblastic) with t(1;22)(p13;q13); RBM15-MKL1 (morphologic abnormality) | leukaemia | 450937003 |
| Acute myeloid leukemia with inv(3)(q21q26.2) or t(3;3)(q21;q26.2); RPN1-EVI1 (disorder) | leukaemia | 780844005 |
| Acute myeloid leukemia with inv(3)(q21q26.2) or t(3;3)(q21;q26.2); RPN1-EVI1 (morphologic abnormality) | leukaemia | 450929006 |
| Philadelphia chromosome-negative precursor B-cell acute lymphoblastic leukemia (disorder) | leukaemia | 714251006 |
| B lymphoblastic leukemia lymphoma with t(12;21)(p13;q22); TEL-AML1 (ETV6-RUNX1) (morphologic abnormality) | leukaemia | 450952005 |
| B lymphoblastic leukemia lymphoma with t(1;19)(q23;p13.3); E2A-PBX1 (TCF3-PBX1) (morphologic abnormality) | leukaemia | 450956008 |
| Acute myeloid leukemia with inv(16)(p13.1q22) or t(16;16)(p13.1;q22) CBFB-MYH11 (disorder) | leukaemia | 838355002 |
| Acute myeloid leukemia and myelodysplastic syndrome related to alkylating agent (disorder) | leukaemia | 766045006 |
| B lymphoblastic leukemia lymphoma with t(12;21) (p13;q22); TEL/AML1 (ETV6-RUNX1) (disorder) | leukaemia | 838342003 |
| Acute myeloid leukemia with mutation of CCAAT enhancer binding protein alpha gene (morphologic abnormality) | leukaemia | 703819004 |
| Disorder of central nervous system co-occurrent and due to acute lymphoid leukemia (disorder) | leukaemia | 61301000119102 |
| Acute myeloid leukemia with biallelic mutation of CCAAT enhancer binding protein alpha gene (morphologic abnormality) | leukaemia | 788740009 |
| Acute myeloid leukemia and myelodysplastic syndrome related to topoisomerase type 2 inhibitor (disorder) | leukaemia | 766046007 |
| Acute myeloid leukemia with multilineage dysplasia without antecedent myelodysplastic syndrome (morphologic abnormality) | leukaemia | 397342007 |
| Therapy-related acute myeloid leukemia and myelodysplastic syndrome, alkylating agent-related type (morphologic abnormality) | leukaemia | 397343002 |
| Therapy related acute myeloid leukemia due to and following administration of antineoplastic agent (disorder) | leukaemia | 762315004 |
| Differentiation syndrome due to and following chemotherapy co-occurrent with acute promyelocytic leukemia (disorder) | leukaemia | 773537001 |
| Therapy-related acute myeloid leukemia and myelodysplastic syndrome, topoisomerase type II inhibitor-related type (morphologic abnormality) | leukaemia | 397344008 |
| Acute myeloid leukemia with multilineage dysplasia following a myelodysplastic syndrome or myelodysplastic syndrome/myeloproliferative disorder (morphologic abnormality) | leukaemia | 397341000 |
| Non-Hodgkin lymphoma (morphologic abnormality) | non-hodgkin's lymphoma | 1172592001 |
| Non-Hodgkin's lymphoma (disorder) | non-hodgkin's lymphoma | 118601006 |
| Diffuse non-Hodgkin's lymphoma (disorder) | non-hodgkin's lymphoma | 109962001 |
| Non-Hodgkin's lymphoma of lung (disorder) | non-hodgkin's lymphoma | 448372003 |
| Non-Hodgkin's lymphoma of bone (disorder) | non-hodgkin's lymphoma | 448220006 |
| Non-Hodgkin's lymphoma of nose (disorder) | non-hodgkin's lymphoma | 448384001 |
| Non-Hodgkin's lymphoma of skin (disorder) | non-hodgkin's lymphoma | 448447004 |
| Non-Hodgkin's lymphoma of ovary (disorder) | non-hodgkin's lymphoma | 448376000 |
| B-cell non-Hodgkin's lymphoma (disorder) | non-hodgkin's lymphoma | 1091921000000103 |
| Non-Hodgkin's lymphoma of testis (disorder) | non-hodgkin's lymphoma | 448387008 |
| Non-Hodgkin's lymphoma of tonsil (disorder) | non-hodgkin's lymphoma | 449292003 |
| Non-Hodgkin's lymphoma of stomach (disorder) | non-hodgkin's lymphoma | 448709005 |
| Non-Hodgkin's lymphoma of intestine (disorder) | non-hodgkin's lymphoma | 448354009 |
| Follicular non-Hodgkin's lymphoma (disorder) | non-hodgkin's lymphoma | 308121000 |
| History of non-Hodgkins lymphoma (situation) | non-hodgkin's lymphoma | 428046009 |
| Non-Hodgkin's lymphoma of prostate (disorder) | non-hodgkin's lymphoma | 449318001 |
| Non-Hodgkin lymphoma in remission (disorder) | non-hodgkin's lymphoma | 143291000119104 |
| Non-Hodgkin's lymphoma of nasopharynx (disorder) | non-hodgkin's lymphoma | 448371005 |
| Non-Hodgkin's lymphoma of soft tissue (disorder) | non-hodgkin's lymphoma | 448738008 |
| Non-Hodgkin's lymphoma of oral cavity (disorder) | non-hodgkin's lymphoma | 448386004 |
| Diffuse non-Hodgkin's lymphoma of skin (disorder) | non-hodgkin's lymphoma | 449217008 |
| Diffuse non-Hodgkin's lymphoma of bone (disorder) | non-hodgkin's lymphoma | 449177007 |
| Diffuse non-Hodgkin's lymphoma of nose (disorder) | non-hodgkin's lymphoma | 449065000 |
| Diffuse non-Hodgkin's lymphoma of lung (disorder) | non-hodgkin's lymphoma | 448867004 |
| Diffuse non-Hodgkin's lymphoma of ovary (disorder) | non-hodgkin's lymphoma | 448609001 |
| Non-Hodgkin's lymphoma of uterine cervix (disorder) | non-hodgkin's lymphoma | 448774004 |
| Diffuse non-Hodgkin's lymphoma of testis (disorder) | non-hodgkin's lymphoma | 448465000 |
| Diffuse non-Hodgkin's lymphoma of stomach (disorder) | non-hodgkin's lymphoma | 448663003 |
| Follicular non-Hodgkin's lymphoma of nose (disorder) | non-hodgkin's lymphoma | 448231003 |
| Non-Hodgkin's lymphoma of extranodal site (disorder) | non-hodgkin's lymphoma | 447989004 |
| Diffuse non-Hodgkin's lymphoma of intestine (disorder) | non-hodgkin's lymphoma | 449176003 |
| Follicular non-Hodgkin's lymphoma of skin (disorder) | non-hodgkin's lymphoma | 448865007 |
| Follicular non-Hodgkin's lymphoma of lung (disorder) | non-hodgkin's lymphoma | 448672006 |
| Follicular non-Hodgkin's lymphoma of bone (disorder) | non-hodgkin's lymphoma | 448666006 |
| Follicular lymphoma grade 3a (disorder) | non-hodgkin's lymphoma | 1148851002 |
| Diffuse non-Hodgkin's lymphoma of prostate (disorder) | non-hodgkin's lymphoma | 448213004 |
| Diffuse non-Hodgkin's lymphoma, small cell (disorder) | non-hodgkin's lymphoma | 109968002 |
| Follicular non-Hodgkin's lymphoma of ovary (disorder) | non-hodgkin's lymphoma | 449307001 |
| Follicular lymphoma grade 3b (disorder) | non-hodgkin's lymphoma | 1148845007 |
| Follicular non-Hodgkin's lymphoma in situ (disorder) | non-hodgkin's lymphoma | 1148847004 |
| Follicular non-Hodgkin's lymphoma of testis (disorder) | non-hodgkin's lymphoma | 449418000 |
| Diffuse non-Hodgkin's lymphoma (disorder) | non-hodgkin's lymphoma | 109962001 |
| Composite Hodgkin and non-Hodgkin lymphoma (disorder) | non-hodgkin's lymphoma | 1156403002 |
| Follicular non-Hodgkin's lymphoma of stomach (disorder) | non-hodgkin's lymphoma | 449222008 |
| Diffuse non-Hodgkin's lymphoma, large cell (disorder) | non-hodgkin's lymphoma | 109969005 |
| Primary oculocerebral non-Hodgkin lymphoma (disorder) | non-hodgkin's lymphoma | 1237578005 |
| Intraocular non-Hodgkin malignant lymphoma (disorder) | non-hodgkin's lymphoma | 420788006 |
| Composite Hodgkin and non-Hodgkin lymphoma (morphologic abnormality) | non-hodgkin's lymphoma | 128798004 |
| Diffuse non-Hodgkin's lymphoma of oral cavity (disorder) | non-hodgkin's lymphoma | 448468003 |
| Follicular non-Hodgkin's lymphoma of tonsil (disorder) | non-hodgkin's lymphoma | 449058008 |
| Non-Hodgkin's lymphoma of tonsil (disorder) | non-hodgkin's lymphoma | 449292003 |
| Diffuse non-Hodgkin's lymphoma, immunoblastic (disorder) | non-hodgkin's lymphoma | 109966003 |
| Diffuse non-Hodgkin's lymphoma of nasopharynx (disorder) | non-hodgkin's lymphoma | 448319002 |
| Diffuse non-Hodgkin's lymphoma of soft tissue (disorder) | non-hodgkin's lymphoma | 449216004 |
| Follicular non-Hodgkin's lymphoma, large cell (disorder) | non-hodgkin's lymphoma | 109972003 |
| Follicular non-Hodgkin's lymphoma of prostate (disorder) | non-hodgkin's lymphoma | 448217003 |
| Diffuse malignant lymphoma - centroblastic (disorder) | non-hodgkin's lymphoma | 302842009 |
| Non-Hodgk+A367:B371in lymphoma in remission (disorder) | non-hodgkin's lymphoma | 143291000119104 |
| Follicular non-Hodgkin's lymphoma of intestine (disorder) | non-hodgkin's lymphoma | 449419008 |
| Diffuse non-Hodgkin's lymphoma of oral cavity (disorder) | non-hodgkin's lymphoma | 448468003 |
| Diffuse non-Hodgkin's lymphoma, undifferentiated (disorder) | non-hodgkin's lymphoma | 109964000 |
| Follicular non-Hodgkin's lymphoma of oral cavity (disorder) | non-hodgkin's lymphoma | 449063007 |
| Follicular non-Hodgkin's lymphoma of intestine (disorder) | non-hodgkin's lymphoma | 449419008 |
| Non-Hodgkin's lymphoma of central nervous system (disorder) | non-hodgkin's lymphoma | 448254007 |
| Follicular non-Hodgkin's lymphoma of nasopharynx (disorder) | non-hodgkin's lymphoma | 449219006 |
| Follicular non-Hodgkin's lymphoma of soft tissue (disorder) | non-hodgkin's lymphoma | 448317000 |
| Diffuse non-Hodgkin's lymphoma of uterine cervix (disorder) | non-hodgkin's lymphoma | 448607004 |
| Diffuse non-Hodgkin's lymphoma of extranodal site (disorder) | non-hodgkin's lymphoma | 448560008 |
| Diffuse non-Hodgkin's lymphoma, small cleaved cell (disorder) | non-hodgkin's lymphoma | 109967007 |
| Diffuse non-Hodgkin's lymphoma of uterine cervix (disorder) | non-hodgkin's lymphoma | 448607004 |
| Follicular non-Hodgkin's lymphoma of uterine cervix (disorder) | non-hodgkin's lymphoma | 449059000 |
| Follicular non-Hodgkin's lymphoma of extranodal site (disorder) | non-hodgkin's lymphoma | 448561007 |
| Follicular non-Hodgkin's lymphoma, small cleaved cell (disorder) | non-hodgkin's lymphoma | 109970006 |
| Diffuse non-Hodgkin's lymphoma of tonsil (disorder) | non-hodgkin's lymphoma | 449173006 |
| Diffuse malignant lymphoma - small non-cleaved cell (disorder) | non-hodgkin's lymphoma | 188674006 |
| Primary non-Hodgkin malignant lymphoma of uveal tract (disorder) | non-hodgkin's lymphoma | 1153346006 |
| Diffuse non-Hodgkin's lymphoma of central nervous system (disorder) | non-hodgkin's lymphoma | 449221001 |
| Non-Hodgkin's lymphoma of lymph nodes of multiple sites (disorder) | non-hodgkin's lymphoma | 352791000119108 |
| Diffuse non-Hodgkin's lymphoma undifferentiated (diffuse) (disorder) | non-hodgkin's lymphoma | 188679001 |
| Primary non-Hodgkin malignant lymphoma of vitreoretinal tract (disorder) | non-hodgkin's lymphoma | 1153347002 |
| Follicular non-Hodgkin's lymphoma of lymph nodes of multiple sites (disorder) | non-hodgkin's lymphoma | 354851000119101 |
| Follicular non-Hodgkin's mixed small cleaved and large cell lymphoma (disorder) | non-hodgkin's lymphoma | 188672005 |
| Non-Hodgkin's lymphoma of lymph nodes of multiple sites (disorder | non-hodgkin's lymphoma | 352791000119108 |
| Malignant lymphoma - mixed small and large cell (disorder) | non-hodgkin's lymphoma | 188676008 |
| Follicular non-Hodgkin's lymphoma diffuse follicle center sub-type grade 1 (disorder) | non-hodgkin's lymphoma | 702786004 |
| Follicular non-Hodgkin's lymphoma, mixed small cleaved cell and large cell (disorder) | non-hodgkin's lymphoma | 109971005 |
| Non-Hodgkin lymphoma associated with Human immunodeficiency virus infection (disorder) | non-hodgkin's lymphoma | 442537007 |
| Follicular non-Hodgkin's lymphoma diffuse follicle center cell sub-type grade 2 (disorder) | non-hodgkin's lymphoma | 702977001 |
| Non-Hodgkin lymphoma of central nervous system metastatic to lymph node of lower limb (disorder) | non-hodgkin's lymphoma | 116811000119106 |
| Non-Hodgkin lymphoma of central nervous system metastatic to lymph node of upper limb (disorder) | non-hodgkin's lymphoma | '116821000119104 |
| Diffuse non-Hodgkin immunoblastic lymphoma co-occurrent with human immunodeficiency virus infection (disorder) | non-hodgkin's lymphoma | 713718006 |
| Malignant lymphoma (disorder) | lymphoma | 118600007 |
| Malignant lymphoma (morphologic abnormality) | lymphoma | 1163043007 |
| Marginal zone B-cell lymphoma (morphologic abnormality) | lymphoma | 128803008 |
| Primary cutaneous CD8 positive aggressive epidermotropic cytotoxic T-cell lymphoma (disorder) | lymphoma | 765136002 |
| Peripheral T-cell lymphoma (morphologic abnormality) | lymphoma | 1163404000 |
| T-zone lymphoma (disorder) | lymphoma | 109975001 |
| T-cell lymphoma (disorder) | lymphoma | 109978004 |
| B-cell lymphoma (disorder) | lymphoma | 109979007 |
| Burkitt's lymphoma (disorder) | lymphoma | 118617000 |
| Orbital lymphoma (disorder) | lymphoma | 13048006 |
| Lymphoma stage I (finding) | lymphoma | 30440004 |
| Gastric lymphoma (disorder) | lymphoma | 276811008 |
| Lymphoepithelioid lymphoma (morphologic abnormality) | lymphoma | 76481008 |
| Burkitt lymphoma (morphologic abnormality) | lymphoma | 77381001 |
| Lymphoma of anus (disorder) | lymphoma | 1153356005 |
| Nodular lymphoma (disorder) | lymphoma | 269476000 |
| Primary cutaneous follicular center B-cell lymphoma (disorder) | lymphoma | 404143002 |
| Malignant lymphoma of liver (disorder) | lymphoma | 1153383006 |
| Lymphoma stage IV (finding) | lymphoma | 66445009 |
| Lymphoma stage Ie (finding) | lymphoma | 89487002 |
| Lymphoma of colon (disorder) | lymphoma | 133751000119102 |
| Lymphoma stage II (finding) | lymphoma | 76422004 |
| Low grade (lymphoma grade) (finding) | lymphoma | 369766003 |
| Lymphoma of retina (disorder) | lymphoma | 232075002 |
| Cutaneous T-cell lymphoma (morphologic abnormality) | lymphoma | 1162973007 |
| Lymphoma of kidney (disorder) | lymphoma | 236513009 |
| Lymphoepithelioid lymphoma (disorder) | lymphoma | 109976000 |
| Natural killer-/T-cell lymphoma, nasal and nasal-type (morphologic abnormality) | lymphoma | 128805001 |
| Lymphoma of pelvis (disorder) | lymphoma | 448553002 |
| Lymphoma stage III (finding) | lymphoma | 56944001 |
| Extranodal natural killer/T-cell lymphoma, nasal type (disorder) | lymphoma | 414166008 |
| History of malignant lymphoma (situation) | lymphoma | 429014004 |
| Lymphoma stage IIIs (finding) | lymphoma | 74053007 |
| Lymphoma with spill (disorder) | lymphoma | 277570003 |
| High grade (lymphoma grade) (finding) | lymphoma | 369767007 |
| Follicular lymphoma (morphologic abnormality) | lymphoma | 55150002 |
| Lymphoma of intestine (disorder) | lymphoma | 276815004 |
| Malignant lymphoma - lymphoplasmacytic (disorder) | lymphoma | 307623001 |
| Malignant lymphoma - small lymphocytic (disorder) | lymphoma | 302841002 |
| Lymphoma stage III 1 (finding) | lymphoma | 112241002 |
| Intravascular large B-cell lymphoma (morphologic abnormality) | lymphoma | 399648005 |
| Mantle cell lymphoma (disorder) | lymphoma | 443487006 |
| Malignant lymphoma - lymphocytic, intermediate differentiation (disorder) | lymphoma | 274905008 |
| Malignant lymphoma, metastatic (morphologic abnormality) | lymphoma | 110459008 |
| Malignant lymphoma, lymphoplasmacytic (morphologic abnormality) | lymphoma | 19340000 |
| Low grade (lymphoma grade) (finding) | lymphoma | 369766003 |
| Malignant lymphoma in remission (disorder) | lymphoma | 427141003 |
| Primary bone lymphoma (disorder) | lymphoma | 766935007 |
| Mucosa-associated lymphoid tissue lymphoma of orbit (disorder) | lymphoma | 414780005 |
| Adult T-cell leukemia/lymphoma (morphologic abnormality) | lymphoma | 77430005 |
| Plasmablastic lymphoma (morphologic abnormality) | lymphoma | 450909005 |
| Lymphoma stage finding (finding) | lymphoma | 385389007 |
| Plasmablastic lymphoma (disorder) | lymphoma | 724648008 |
| Splenic B-cell lymphoma (morphologic abnormality) | lymphoma | 734141009 |
| Lymphoma of small intestine (disorder) | lymphoma | 449074003 |
| Lymphoma involves liver (finding) | lymphoma | 397451000 |
| Lymphomatous meningitis (disorder) | lymphoma | 426128009 |
| Malignant lymphoma of uveal tract (disorder) | lymphoma | 1162264008 |
| African Burkitt's lymphoma (disorder) | lymphoma | 240531002 |
| Endemic Burkitt's lymphoma (morphologic abnormality) | lymphoma | 419770008 |
| Lymphoma involves spleen (finding) | lymphoma | 397450004 |
| Lymphoma of sigmoid colon (disorder) | lymphoma | 449218003 |
| Low grade T-cell lymphoma (disorder) | lymphoma | 277642008 |
| Follicular lymphoma (morphologic abnormality) | lymphoma | 55150002 |
| Atypical Burkitt's lymphoma (morphologic abnormality) | lymphoma | 419879004 |
| Primary effusion lymphoma (morphologic abnormality) | lymphoma | 128800006 |
| Sporadic Burkitt's lymphoma (morphologic abnormality) | lymphoma | 420063007 |
| Primary effusion lymphoma (disorder) | lymphoma | 713516007 |
| B-cell Hodgkin's lymphoma (disorder) | lymphoma | 1091891000000106 |
| Lymphomatous infiltration (morphologic abnormality) | lymphoma | 445925008 |
| Low grade B-cell lymphoma (disorder) | lymphoma | 277615007 |
| Histiocytic sarcoma (morphologic abnormality) | lymphoma | 128813000 |
| Primary cutaneous marginal zone B-cell lymphoma (disorder) | lymphoma | 404140004 |
| Primary cerebral lymphoma (disorder) | lymphoma | 276836002 |
| B-cell lymphoma 6 protein (substance) | lymphoma | 1228964008 |
| Histiocytic sarcoma (disorder) | lymphoma | 109988003 |
| Primary cutaneous B-cell lymphoma (disorder) | lymphoma | 402881008 |
| Primary cutaneous T-cell lymphoma (disorder) | lymphoma | 400122007 |
| Lymphoma of gastrointestinal tract (disorder) | lymphoma | 449072004 |
| Burkitt lymphoma/leukemia (morphologic abnormality) | lymphoma | 397400006 |
| Mucosa-associated lymphoma (disorder) | lymphoma | 277622004 |
| High grade T-cell lymphoma (disorder) | lymphoma | 277643003 |
| Non-Hodgkin lymphoma (morphologic abnormality) | lymphoma | 1172592001 |
| Burkitt's lymphoma of spleen (disorder) | lymphoma | 188516007 |
| History of B-cell lymphoma (situation) | lymphoma | 427846005 |
| Lymphoma finding (finding) | lymphoma | 399600009 |
| Follicular T-cell lymphoma (morphologic abnormality) | lymphoma | 784551004 |
| Primary pulmonary lymphoma (disorder) | lymphoma | 718200007 |
| Monocytoid B-cell lymphoma (disorder) | lymphoma | 277623009 |
| Primary cutaneous lymphoma (morphologic abnormality) | lymphoma | 419392005 |
| Intestinal T-cell lymphoma (morphologic abnormality) | lymphoma | 103686008 |
| Follicular low grade B-cell lymphoma (disorder) | lymphoma | 277618009 |
| High grade B-cell lymphoma (disorder) | lymphoma | 277617004 |
| Malignant lymphoma of lacrimal gland (disorder) | lymphoma | 1153382001 |
| Nodular lymphoma of spleen (disorder) | lymphoma | 95193005 |
| Classical Hodgkin lymphoma (morphologic abnormality) | lymphoma | 762691001 |
| T-cell lymphoma morphology (morphologic abnormality) | lymphoma | 314926009 |
| Classical Hodgkin lymphoma (disorder) | lymphoma | 762690000 |
| Peripheral T-cell lymphoma (disorder) | lymphoma | 109977009 |
| B-cell lymphoma morphology (morphologic abnormality) | lymphoma | 314922006 |
| Primary cutaneous lymphoma (disorder) | lymphoma | 400001003 |
| Lymphoma of lower esophagus (disorder) | lymphoma | 449053004 |
| Follicular lymphoma grade 2 (disorder) | lymphoma | 847631000000107 |
| B-cell lymphoma 2 inhibitor (substance) | lymphoma | 725567006 |
| Follicular lymphoma grade 1 (disorder) | lymphoma | 847481000000109 |
| Follicular lymphoma grade 3 (disorder) | lymphoma | 847651000000100 |
| Intravascular large B-cell lymphoma (disorder) | lymphoma | 1157162007 |
| Lymphoma of small intestine (disorder) | lymphoma | 449074003 |
| Angioimmunoblastic T-cell lymphoma (morphologic abnormality) | lymphoma | 835009 |
| Low grade lymphoma, stage 1 (finding) | lymphoma | 425037004 |
| Lymphoma of body of stomach (disorder) | lymphoma | 448555009 |
| Mediastinal large B-cell lymphoma (morphologic abnormality) | lymphoma | 128801005 |
| Follicular lymphoma, grade 3 (morphologic abnormality) | lymphoma | 1155957006 |
| Follicular lymphoma grade 3a (morphologic abnormality) | lymphoma | 1155956002 |
| Follicular lymphoma, grade 2 (morphologic abnormality) | lymphoma | 55020008 |
| Lymphoma stage IIe (finding) | lymphoma | 5701003 |
| Follicular lymphoma grade 3b (disorder) | lymphoma | 1148845007 |
| Follicular lymphoma grade 3a (disorder) | lymphoma | 1148851002 |
| Malignant lymphoma of breast (disorder) | lymphoma | 278052009 |
| Malignant lymphoma of testis (disorder) | lymphoma | 277664004 |
| Follicular lymphoma, grade 1 (morphologic abnormality) | lymphoma | 46744002 |
| Malignant lymphoma of spleen (disorder) | lymphoma | 93198004 |
| Lymphoma stage IIIe (finding) | lymphoma | 44255004 |
| Hepatosplenic T-cell lymphoma (disorder) | lymphoma | 445406001 |
| Diffuse large B-cell lymphoma (disorder) | lymphoma | 847741000000106 |
| Lymphoma involves bone marrow (finding) | lymphoma | 397452007 |
| Diffuse large B cell malignant lymphoma (morphologic abnormality) | lymphoma | 1172695008 |
| History of Hodgkin lymphoma (situation) | lymphoma | 473068004 |
| Lymphoma stage III 2 (finding) | lymphoma | 75339006 |
| Splenic marginal zone B-cell lymphoma (morphologic abnormality) | lymphoma | 128802003 |
| Sporadic Burkitt's lymphoma (morphologic abnormality) | lymphoma | 128802003 |
| Mantle cell lymphoma of spleen (disorder) | lymphoma | 441559006 |
| Suspected lymphoma (situation) | lymphoma | 315264005 |
| Lymphoma with spill (disorder) | lymphoma | 277570003 |
| Malignant lymphoma of duodenum (disorder) | lymphoma | 1153354008 |
| Lymphoma of pylorus of stomach (disorder) | lymphoma | 447656001 |
| Follicular lymphoma grade 3a (disorder) | lymphoma | 1155957006 |
| Malignant lymphoma, small lymphocytic (morphologic abnormality) | lymphoma | 64575004 |
| Lymphoma extent of involvement (observable entity) | lymphoma | 397447002 |
| Primary lymphoma of conjunctiva (disorder) | lymphoma | 763477007 |
| Lymphoma of appendix (disorder) | lymphoma | 1153357001 |
| Malignant lymphoma of esophagus (disorder) | lymphoma | 1153355009 |
| Follicular non-Hodgkin's lymphoma (disorder) | lymphoma | 308121000 |
| Hodgkin's disease in remission (disorder) | lymphoma | 426071002 |
| Non-Hodgkin's lymphoma of testis (disorder) | lymphoma | 448387008 |
| Mantle cell lymphoma (morphologic abnormality) | lymphoma | 74654000 |
| Precursor cell lymphoblastic lymphoma (disorder) | lymphoma | 1217301006 |
| Marginal zone lymphoma of spleen (disorder) | lymphoma | 116691000119101 |
| Lymphomatoid granulomatosis (disorder) | lymphoma | 239940004 |
| History of non-Hodgkins lymphoma (situation) | lymphoma | 428046009 |
| Diffuse follicle center lymphoma (disorder) | lymphoma | 449220000 |
| Lymphoma of intestine (disorder) | lymphoma | 276815004 |
| Enteropathy-associated T-cell lymphoma (disorder) | lymphoma | 277654008 |
| Malignant lymphoma - centrocytic (disorder) | lymphoma | 307625008 |
| Primary cutaneous T-cell lymphoma (disorder) | lymphoma | 400122007 |
| Non-Hodgkin's lymphoma of tonsil (disorder) | lymphoma | 449292003 |
| Non-Hodgkin's lymphoma of prostate (disorder) | lymphoma | 449318001 |
| Non-Hodgkin's lymphoma (disorder) | lymphoma | 118601006 |
| Lymphoma involves liver (finding) | lymphoma | 397451000 |
| T-cell/histiocyte rich large B-cell lymphoma (morphologic abnormality) | lymphoma | 450959001 |
| Immunoproliferative small intestinal disease (disorder) | lymphoma | 109985000 |
| Primary cutaneous T-cell lymphoma (morphologic abnormality) | lymphoma | 1187136005 |
| Lymphoma of retroperitoneal space (disorder) | lymphoma | 422853008 |
| Hodgkin lymphoma, lymphocyte-rich (disorder) | lymphoma | 118607005 |
| Hodgkin lymphoma, lymphocyte-rich (morphologic abnormality) | lymphoma | 128799007 |
| Primary cutaneous B-cell lymphoma (morphologic abnormality) | lymphoma | 1187129009 |
| Precursor cell lymphoblastic lymphoma (morphologic abnormality) | lymphoma | 128806000 |
| Lymphomatous infiltrate of kidney (disorder) | lymphoma | 836486002 |
| Marginal zone lymphoma (disorder) | lymphoma | 447100004 |
| Malignant lymphoma of lymph nodes (disorder) | lymphoma | 127220001 |
| B lymphoblastic leukemia lymphoma (morphologic abnormality) | lymphoma | 1162660006 |
| Sézary's disease of intra-abdominal lymph nodes (disorder) | lymphoma | 188632001 |
| Precursor T cell lymphoblastic leukemia/lymphoblastic lymphoma (morphologic abnormality) | lymphoma | 397348006 |
| Diffuse low grade B-cell lymphoma (disorder) | lymphoma | 277616008 |
| Intermediate grade B-cell lymphoma (disorder) | lymphoma | 285776004 |
| Nodular high grade B-cell lymphoma (disorder) | lymphoma | 277627005 |
| Anaplastic lymphoma kinase positive large B-cell lymphoma (disorder) | lymphoma | 715950008 |
| Diffuse high grade B-cell lymphoma (disorder) | lymphoma | 277626001 |
| B cell lymphoma of small intestine (disorder) | lymphoma | 1196900003 |
| T-cell lymphoma of small intestine (disorder) | lymphoma | 1197344000 |
| Anaplastic lymphoma kinase positive large B-cell lymphoma (morphologic abnormality) | lymphoma | 450910000 |
| Hodgkin's disease, mixed cellularity (disorder) | lymphoma | 118609008 |
| Malignant lymphoma, stem cell type (morphologic abnormality) | lymphoma | 189962004 |
| Follicular lymphoma, cutaneous follicle centre (disorder) | lymphoma | 1091861000000100 |
| Angioimmunoblastic T-cell lymphoma (disorder) | lymphoma | 413537009 |
| Anaplastic large cell lymphoma, T cell and Null cell type (morphologic abnormality) | lymphoma | 53237008 |
| Nodal marginal zone B-cell lymphoma (disorder) | lymphoma | 726721002 |
| Hodgkin lymphoma, mixed cellularity (morphologic abnormality) | lymphoma | 41529000 |
| Large cell anaplastic lymphoma T cell and Null cell type (disorder) | lymphoma | 702785000 |
| Adenolymphoma (disorder) | lymphoma | 422470007 |
| Malignant lymphoma of thyroid gland (disorder) | lymphoma | 278051002 |
| Nodal marginal zone B-cell lymphoma (morphologic abnormality) | lymphoma | 397349003 |
| Hodgkin lymphoma, nodular sclerosis (morphologic abnormality) | lymphoma | 52248008 |
| Lymphomatoid granulomatosis grade 1 (morphologic abnormality) | lymphoma | 789178005 |
| Lymphomatoid granulomatosis grade 2 (morphologic abnormality) | lymphoma | 789179002 |
| Spongiotic mycosis fungoides (disorder) | lymphoma | 404117003 |
| Site of direct extension of lymphoma (observable entity) | lymphoma | 399620005 |
| Low grade B-cell lymphoma morphology (morphologic abnormality) | lymphoma | 314923001 |
| Angiocentric natural killer/T-cell malignant lymphoma involving skin (disorder) | lymphoma | 404135007 |
| Lymphomatoid papulosis type B - mycosis fungoides-like (disorder) | lymphoma | 404104001 |
| Cutaneous/peripheral T-cell lymphoma (disorder) | lymphoma | 277613000 |
| Low grade T-cell lymphoma morphology (morphologic abnormality) | lymphoma | 314930007 |
| Relapsing classical Hodgkin lymphoma (disorder) | lymphoma | 830057003 |
| Malignant lymphoma of the eye region (disorder) | lymphoma | 420519005 |
| B-cell lymphoma 6 protein (substance) | lymphoma | 1228964008 |
| History of primary cutaneous lymphoma (situation) | lymphoma | 789395008 |
| Lymphoma of pyloric antrum of stomach (disorder) | lymphoma | 447766003 |
| High grade B-cell lymphoma morphology (morphologic abnormality) | lymphoma | 314925008 |
| High grade T-cell lymphoma morphology (morphologic abnormality) | lymphoma | 314925008 |
| Splenic marginal zone B-cell lymphoma (disorder) | lymphoma | 763666008 |
| Tumor stage mycosis fungoides (disorder) | lymphoma | 404113004 |
| Non-Hodgkin's lymphoma of soft tissue (disorder) | lymphoma | 448738008 |
| Non-Hodgkin's lymphoma of nasopharynx (disorder) | lymphoma | 448371005 |
| Lymphoma of cardioesophageal junction (disorder) | lymphoma | 449075002 |
| Malignant lymphomatoid granulomatosis (disorder) | lymphoma | 789689004 |
| Malignant lymphomatoid granulomatosis (morphologic abnormality) | lymphoma | 878856000 |
| Precursor B-lymphoblastic leukemia/lymphoblastic lymphoma (morphologic abnormality) | lymphoma | 397347001 |
| Follicular lymphoma of small intestine (disorder) | lymphoma | 721555001 |
| Diffuse malignant lymphoma - centroblastic (disorder) | lymphoma | 302842009 |
| Enteropathy-associated T-cell lymphoma (morphologic abnormality) | lymphoma | 1222550009 |
| Malignant lymphoma, lymphoplasmacytic (morphologic abnormality) | lymphoma | 302841002 |
| Hodgkin lymphoma, lymphocyte depletion (morphologic abnormality) | lymphoma | 112687003 |
| Lymphoma extranodal involvement status (attribute) | lymphoma | 260923007 |
| Primary cutaneous large T-cell lymphoma (morphologic abnormality) | lymphoma | 1187135009 |
| Erythrodermic mycosis fungoides (disorder) | lymphoma | 404114005 |
| Syringotropic mycosis fungoides (disorder) | lymphoma | 404118008 |
| Microglioma (disorder) | lymphoma | 307649006 |
| Primary cutaneous large T-cell lymphoma (disorder) | lymphoma | 402880009 |
| Lymphoma of lesser curvature of stomach (disorder) | lymphoma | 448269008 |
| Follicular non-Hodgkin's lymphoma of lung (disorder) | lymphoma | 448672006 |
| Follicular non-Hodgkin's lymphoma of bone (disorder) | lymphoma | 448666006 |
| Malignant lymphoma of spleen (disorder) | lymphoma | 277664004 |
| Precursor B-cell lymphoblastic lymphoma (morphologic abnormality) | lymphoma | 128807009 |
| Hypomelanotic mycosis fungoides (disorder) | lymphoma | 404110001 |
| Precursor T-cell lymphoblastic lymphoma (morphologic abnormality) | lymphoma | 128808004 |
| Hepatosplenic gamma-delta cell lymphoma (disorder) | lymphoma | 699657009 |
| Granulomatous mycosis fungoides (disorder) | lymphoma | 404112009 |
| Malignant lymphoma - small cleaved cell (disorder) | lymphoma | 188675007 |
| Hepatosplenic gamma-delta cell lymphoma (morphologic abnormality) | lymphoma | 103685007 |
| Diffuse large B-cell lymphoma of stomach (disorder) | lymphoma | 840424008 |
| Organ AND/OR tissue involved by lymphoma (observable entity) | lymphoma | 406093003 |
| CD-30 negative cutaneous T-cell lymphoma (disorder) | lymphoma | 404128004 |
| Follicular lymphoma, grade 3 (morphologic abnormality) | lymphoma | 40411000 |
| Lymphoma of greater curvature of stomach (disorder) | lymphoma | 447805007 |
| Lymphoma of fundus of stomach (disorder) | lymphoma | 447658000 |
| Lymphomatoid granulomatosis of lung (disorder) | lymphoma | 239297008 |
| Anaplastic diffuse large B-cell lymphoma (morphologic abnormality) | lymphoma | 1172702009 |
| Splenic schistosomal giant cell lymphoma (disorder) | lymphoma | 240794006 |
| Lymphoma of cardia of stomach (disorder) | lymphoma | 447806008 |
| Malignant lymphoma, convoluted cell type (disorder) | lymphoma | 303017006 |
| Subcutaneous panniculitic T-cell lymphoma (morphologic abnormality) | lymphoma | 103682005 |
| Splenic lymphoma with villous lymphocytes (disorder) | lymphoma | 277551008 |
| Burkitt-like lymphoma with 11q aberration (morphologic abnormality) | lymphoma | 783220004 |
| Malignant lymphoma of lymph nodes of head (disorder) | lymphoma | 1255805006 |
| Malignant lymphoma of lymph nodes of neck (disorder) | lymphoma | 1255806007 |
| Diffuse non-Hodgkin's lymphoma of prostate (disorder) | lymphoma | 448213004 |
| Polyglandular autoimmune syndrome, type 2 (disorder) | lymphoma | 83728000 |
| Primary cutaneous follicle center cell lymphoma (morphologic abnormality) | lymphoma | 419662008 |
| Composite Hodgkin and non-Hodgkin lymphoma (disorder) | lymphoma | 1156403002 |
| Hodgkin lymphoma (morphologic abnormality) | lymphoma | 1163005009 |
| Malignant lymphoma, follicular center cell (disorder) | lymphoma | 303055001 |
| Poikilodermatous mycosis fungoides (disorder) | lymphoma | 404108003 |
| Primary mediastinal (thymic) large B-cell lymphoma (disorder) | lymphoma | 444910004 |
| Nodular lymphoma of intrapelvic lymph nodes (disorder) | lymphoma | 95187002 |
| Burkitt's lymphoma of intrapelvic lymph nodes (disorder) | lymphoma | 188515006 |
| Hodgkin lymphoma, nodular sclerosis, grade 2 (morphologic abnormality) | lymphoma | 43985008 |
| Patch/plaque stage mycosis fungoides (disorder) | lymphoma | 404107008 |
| B-cell lymphoma of lymph nodes of multiple sites (disorder) | lymphoma | 351211000119104 |
| Malignant lymphoma, mixed small and large cell, diffuse (morphologic abnormality) | lymphoma | 50102004 |
| T-cell histiocyte rich large B-cell lymphoma (disorder) | lymphoma | 724645006 |
| Immunodeficiency-associated Burkitt's lymphoma (morphologic abnormality) | lymphoma | 419094004 |
| Diffuse low grade B-cell lymphoma morphology (morphologic abnormality) | lymphoma | 314929002 |
| Lymphoma involves a single lymph node region (finding) | lymphoma | 397448007 |
| Hodgkin lymphoma, nodular sclerosis, grade 1 (morphologic abnormality) | lymphoma | 45572000 |
| Lymphoma extent of involvement not specified (finding) | lymphoma | 405988006 |
| Lymphoma confined to mucosa AND/OR submucosa (finding) | lymphoma | 399430006 |
| Diffuse high grade B-cell lymphoma morphology (morphologic abnormality) | lymphoma | 314934003 |
| Burkitt's lymphoma of intrathoracic lymph nodes (disorder) | lymphoma | 188511002 |
| Primary cutaneous gamma-delta T-cell lymphoma (morphologic abnormality) | lymphoma | 450908002 |
| Primary cutaneous CD30 antigen positive large T-cell lymphoma (disorder) | lymphoma | 128875000 |
| Large B-cell lymphoma with interferon regulatory factor 4 rearrangement (morphologic abnormality) | lymphoma | 786960000 |
| Nodular high grade B-cell lymphoma morphology (morphologic abnormality) | lymphoma | 314931006 |
| Prethymic and thymic T-cell lymphoma/leukemia (disorder) | lymphoma | 277614006 |
| Malignant lymphoma of intrapelvic lymph nodes (disorder) | lymphoma | 93192003 |
| B lymphoblastic leukemia lymphoma with intrachromosomal amplification of chromosome 21 (morphologic abnormality) | lymphoma | 785825000 |
| Malignant lymphomatoid granulomatosis grade 3 (disorder) | lymphoma | 878857009 |
| Nodular lymphoma of intrathoracic lymph nodes (disorder) | lymphoma | 95188007 |
| Angioimmunoblastic T-cell lymphoma (disorder) | lymphoma | 413537009 |
| Malignant lymphomatoid granulomatosis of lung (disorder) | lymphoma | 789690008 |
| Primary cutaneous plasmacytoma (disorder) | lymphoma | 404142007 |
| Lymphoma involves multiple lymph node regions (finding) | lymphoma | 397449004 |
| Diffuse non-Hodgkin's lymphoma of oral cavity (disorder) | lymphoma | 448468003 |
| Malignant lymphomatoid granulomatosis grade 3 (morphologic abnormality) | lymphoma | 788566005 |
| Surgical proximal margin involved by lymphoma (finding) | lymphoma | 399673005 |
| Burkitt lymphoma of lymph nodes of lower limb (disorder) | lymphoma | 1255895001 |
| Burkitt lymphoma of lymph nodes of upper limb (disorder) | lymphoma | 1255895001 |
| Lymphomatoid papulosis with Hodgkin's disease (disorder) | lymphoma | 404106004 |
| Primary cutaneous gamma-delta-positive T-cell lymphoma (disorder) | lymphoma | 733627006 |
| Malignant lymphoma, follicular AND/OR nodular (morphologic abnormality) | lymphoma | 115245001 |
| Large cell lymphoma of intrapelvic lymph nodes (disorder) | lymphoma | 441962003 |
| B-cell lymphoma of intra-abdominal lymph nodes (disorder) | lymphoma | 350951000119101 |
| History of malignant cutaneous T-cell lymphoma (situation) | lymphoma | 122571000119106 |
| Subcutaneous panniculitic cutaneous T-cell lymphoma (disorder) | lymphoma | 404133000 |
| Splenic diffuse red pulp small B-cell lymphoma (morphologic abnormality) | lymphoma | 734067001 |
| Splenic diffuse red pulp small B-cell lymphoma (disorder) | lymphoma | 763884007 |
| Malignant lymphoma of lymph nodes of lower limb (disorder) | lymphoma | 1255907004 |
| Malignant lymphoma - mixed small and large cell (disorder) | lymphoma | 188676008 |
| Malignant lymphoma of intrathoracic lymph nodes (disorder) | lymphoma | 93193008 |
| B-lymphoblastic leukemia lymphoma BCR-ABL1-like (morphologic abnormality) | lymphoma | 783744003 |
| Diffuse malignant lymphoma - large cleaved cell (disorder) | lymphoma | 277628000 |
| Diffuse malignant lymphoma - small non-cleaved cell (disorder) | lymphoma | 188674006 |
| Primary cutaneous marginal zone B-cell lymphoma (morphologic abnormality) | lymphoma | 420028002 |
| Follicular malignant lymphoma - mixed cell type (disorder) | lymphoma | 277624003 |
| Follicular low grade B-cell lymphoma morphology (morphologic abnormality) | lymphoma | 314924007 |
| Nodular lymphoma of intra-abdominal lymph nodes (disorder) | lymphoma | 95186006 |
| Burkitt's lymphoma of intra-abdominal lymph nodes (disorder) | lymphoma | 188512009 |
| Malignant lymphoma of lymph nodes of upper limb (disorder) | lymphoma | 1255906008 |
| Diffuse large B-cell lymphoma of small intestine (disorder) | lymphoma | 840423002 |
| Primary cutaneous anaplastic large cell lymphoma (disorder) | lymphoma | 773995001 |
| Lymphoma extralymphatic organ involvement status (attribute) | lymphoma | 260924001 |
| Lymphoma involves muscular wall AND/OR subserosa (finding) | lymphoma | 399485005 |
| Small lymphocytic B-cell lymphoma involving skin (disorder) | lymphoma | 404138009 |
| Primary cutaneous anaplastic large T-cell lymphoma, CD30-positive (morphologic abnormality) | lymphoma | 397352006 |
| CD-30 positive T-immunoblastic cutaneous lymphoma (disorder) | lymphoma | 404127009 |
| Nodular lymphoma of lymph nodes of multiple sites (disorder) | lymphoma | 95192000 |
| CD-30 negative T-immunoblastic cutaneous lymphoma (disorder) | lymphoma | 404131003 |
| Malignant lymphoma of intra-abdominal lymph nodes (disorder) | lymphoma | 93191005 |
| Hodgkin lymphoma, nodular lymphocyte predominance (disorder) | lymphoma | 118605002 |
| Burkitt's lymphoma of lymph nodes of multiple sites (disorder) | lymphoma | 188517003 |
| Lymphomatous extranodal involvement status values (tumor staging) | lymphoma | 258280004 |
| Lymphoma staging symptom status B (tumor staging) | lymphoma | 421211000 |
| Hodgkin lymphoma, lymphocyte depletion, reticular (morphologic abnormality) | lymphoma | 71109004 |
| Hodgkin lymphoma, nodular lymphocyte predominance (morphologic abnormality) | lymphoma | 70600005 |
| Lymphoplasmacytic lymphoma without immunoglobulin M production (disorder) | lymphoma | 1255270006 |
| Malignant lymphoma of lymph nodes of head and neck (disorder) | lymphoma | 1255807003 |
| Malignant lymphoma, centroblastic type, follicular (disorder) | lymphoma | 307647008 |
| Precursor T-cell lymphoblastic lymphoma (disorder) | lymphoma | 421246008 |
| Follicular malignant lymphoma - small cleaved cell (disorder) | lymphoma | 277625002 |
| Hydroa vacciniforme-like cutaneous T-cell lymphoma (disorder) | lymphoma | 1153397003 |
| Malignant lymphoma, follicular center cell, cleaved (disorder) | lymphoma | 303056000 |
| Anaplastic large T-cell systemic malignant lymphoma (disorder) | lymphoma | 404134006 |
| B lymphoblastic leukemia lymphoma with hypodiploidy (disorder) | lymphoma | 838344002 |
| Diffuse malignant lymphoma - large non-cleaved cell (disorder) | lymphoma | 277629008 |
| Laparoscopic staging of Hodgkin's disease or lymphoma (procedure) | lymphoma | 31726006 |
| Lymphomatoid papulosis-associated mycosis fungoides (disorder) | lymphoma | 404111002 |
| Hodgkin lymphoma, nodular sclerosis, cellular phase (morphologic abnormality) | lymphoma | 39086001 |
| Malignant lymphoma of lymph nodes of multiple sites (disorder) | lymphoma | 93197009 |
| Mycosis fungoides with systemic infiltration (disorder) | lymphoma | 404116007 |
| B lymphoblastic leukemia lymphoma with hyperdiploidy (morphologic abnormality) | lymphoma | 450953000 |
| B lymphoblastic leukemia lymphoma with hyperdiploidy (disorder) | lymphoma | 838346000 |
| Lymphomatous tumor, benign (morphologic abnormality) | lymphoma | 189958005 |
| Primary cutaneous acral CD8 positive T-cell lymphoma (morphologic abnormality) | lymphoma | 787198005 |
| Surgical circumferential margin involved by lymphoma (finding) | lymphoma | 399457008 |
| Primary cutaneous diffuse large cell B-cell lymphoma (disorder) | lymphoma | 404144008 |
| Malignant lymphomatoid granulomatosis grade 3 of lung (disorder) | lymphoma | 878858004 |
| Specified cutaneous AND/OR peripheral T cell lymphoma (morphologic abnormality) | lymphoma | 115246000 |
| Surgical distal margin involved by lymphoma (finding) | lymphoma | 399545001 |
| B lymphoblastic leukemia lymphoma with hyperdiploidy (disorder) | lymphoma | 450953000 |
| Diffuse large B cell malignant lymphoma (morphologic abnormality) | lymphoma | 450953000 |
| B lymphoblastic leukemia lymphoma with hyperdiploidy (morphologic abnormality) | lymphoma | 1172695008 |
| Mantle cell lymphoma of lymph nodes of multiple sites (disorder) | lymphoma | 116871000119103 |
| Monomorphic epitheliotropic intestinal T-cell lymphoma (morphologic abnormality) | lymphoma | 787036009 |
| Precursor B-cell lymphoblastic lymphoma involving skin (disorder) | lymphoma | 404137004 |
| Diffuse malignant lymphoma - centroblastic-centrocytic (disorder) | lymphoma | 307624007 |
| Lymphoma extent of involvement not specified (finding) | lymphoma | 405988006 |
| Diffuse malignant lymphoma - centroblastic polymorphic (disorder) | lymphoma | 277632006 |
| Diffuse large B-cell lymphoma activated B-cell subtype (morphologic abnormality) | lymphoma | 787565006 |
| Follicular lymphoma grade 3b (morphologic abnormality) | lymphoma | 1155957006 |
| Primary cutaneous follicular center B-cell lymphoma (disorder) | lymphoma | 733627006 |
| Primary cutaneous gamma-delta-positive T-cell lymphoma (disorder) | lymphoma | 404143002 |
| Nodular lymphoma of lymph nodes of head, face and neck (disorder) | lymphoma | 188609000 |
| Malignant lymphoma, diffuse large B-cell, immunoblastic (morphologic abnormality) | lymphoma | 450958009 |
| Angioimmunoblastic T-cell lymphoma with dysproteinaemia (disorder) | lymphoma | 1090241000000100 |
| Diffuse large B-cell lymphoma of central nervous system (disorder) | lymphoma | 734066005 |
| Malignant lymphoma, follicular center cell, non-cleaved (disorder) | lymphoma | 303057009 |
| Marginal zone lymphoma of lymph nodes of multiple sites (disorder) | lymphoma | 116841000119105 |
| Follicular lymphoma, cutaneous follicle center sub-type (morphologic abnormality) | lymphoma | 397467006 |
| Lymphoma involves multiple lymph node regions (finding) | lymphoma | 397449004 |
| Burkitt lymphoma of lymph nodes of lower limb (disorder) | lymphoma | 1255796007 |
| Nodular lymphoma of lymph nodes of axilla and upper limb (disorder) | lymphoma | 188612002 |
| Breast implant-associated anaplastic large cell lymphoma (morphologic abnormality) | lymphoma | 1172730009 |
| Hodgkin lymphoma, lymphocyte depletion, diffuse fibrosis (morphologic abnormality) | lymphoma | 16893006 |
| Breast implant–associated anaplastic large-cell lymphoma (disorder) | lymphoma | 783541009 |
| Primary cutaneous diffuse large B-cell lymphoma leg type (morphologic abnormality) | lymphoma | 1187190002 |
| Follicular non-Hodgkin's lymphoma of prostate (disorder) | lymphoma | 93199007 |
| Malignant lymphoma of extranodal AND/OR solid organ site (disorder) | lymphoma | 93199007 |
| Malignant lymphoma, centroblastic-centrocytic, follicular (disorder) | lymphoma | 307637005 |
| Diffuse large B cell malignant lymphoma (morphologic abnormality) | lymphoma | 1172695008 |
| CD-30 negative anaplastic large T-cell cutaneous lymphoma (disorder) | lymphoma | 404129007 |
| Surgical margin involvement by lymphoma cannot be assessed (finding) | lymphoma | 399676002 |
| CD-30 positive pleomorphic large T-cell cutaneous lymphoma (disorder) | lymphoma | 404126000 |
| CD-30 negative pleomorphic large T-cell cutaneous lymphoma (disorder) | lymphoma | 404130002 |
| B-cell chronic lymphocytic leukemia/small lymphocytic lymphoma (morphologic abnormality) | lymphoma | 51092000 |
| Burkitt's lymphoma of intrathoracic lymph nodes (disorder) | lymphoma | 188511002 |
| Malignant lymphoma, mixed lymphocytic-histiocytic, nodular (disorder) | lymphoma | 307636001 |
| Malignant lymphoma, large cell, polymorphous, immunoblastic (disorder) | lymphoma | 371134001 |
| Spleen-positive minimal lymphomatous extranodal involvement (tumor staging) | lymphoma | 258281000 |
| Peripheral T-cell lymphoma of lymph nodes of multiple sites (disorder) | lymphoma | 117211000119105 |
| Gastrointestinal lymphoma surgical margin, involved by tumor (finding) | lymphoma | 369702004 |
| Malignant lymphoma, stem cell type (morphologic abnormality) | lymphoma | 189962004 |
| Diffuse large B-cell lymphoma germinal centre B-cell subtype (morphologic abnormality) | lymphoma | 787594004 |
| Extranodal marginal zone lymphoma of mucosa-associated lymphoid tissue of stomach (disorder) | lymphoma | 444597005 |
| Primary cutaneous T-cell lymphoma, large cell, CD30-negative (morphologic abnormality) | lymphoma | 419586003 |
| Hodgkin lymphoma, lymphocyte depletion of lymph nodes of neck (disorder) | lymphoma | 1255666008 |
| Nodular malignant lymphoma, lymphocytic - well differentiated (disorder) | lymphoma | 302845006 |
| Spleen-positive extensive lymphomatous extranodal involvement (tumor staging) | lymphoma | 258282007 |
| Primary cutaneous diffuse large cell B-cell lymphoma of lower extremity (disorder) | lymphoma | 735332000 |
| Small lymphocytic B-cell lymphoma of lymph nodes of multiple sites (disorder) | lymphoma | 352251000119109 |
| Follicular lymphoma, diffuse follicle center sub-type, grade 1 (morphologic abnormality) | lymphoma | 397468001 |
| Anaplastic lymphoma kinase fusion oncogene negative non-small cell lung cancer (disorder) | lymphoma | 830055006 |
| Anaplastic lymphoma kinase fusion oncogene positive non-small cell lung cancer (disorder) | lymphoma | 830151004 |
| Follicular non-Hodgkin's lymphoma of uterine cervix (disorder) | lymphoma | 449059000 |
| Precursor T cell lymphoblastic leukemia/lymphoblastic lymphoma (disorder) | lymphoma | 420890002 |
| Lymphomatoid papulosis-associated mycosis fungoides (disorder) | lymphoma | 404111002 |
| Malignant lymphoma of lymph nodes of multiple sites (disorder) | lymphoma | 93197009 |
| Malignant lymphoma, lymphocytic, poorly differentiated, nodular (disorder) | lymphoma | 307646004 |
| High grade T-cell lymphoma morphology (morphologic abnormality) | lymphoma | 314927000 |
| High grade B-cell lymphoma morphology (morphologic abnormality) | lymphoma | 314927000 |
| Small lymphocytic B-cell lymphoma of intra-abdominal lymph nodes (disorder) | lymphoma | 352411000119109 |
| B lymphoblastic leukemia lymphoma with t(5;14)(q31;q32); IL3-IGH (disorder) | lymphoma | 450955007 |
| Nodular lymphoma of lymph nodes of inguinal region and lower limb (disorder) | lymphoma | 188613007 |
| B lymphoblastic leukemia lymphoma with t(v;11q23); MLL rearranged (disorder) | lymphoma | 838341005 |
| B lymphoblastic leukemia lymphoma with t(5;14)(q31;q32); IL3-IGH (disorder) | lymphoma | 838340006 |
| Primary lymphoma of brain with acquired immunodeficiency syndrome (disorder) | lymphoma | 421283008 |
| Mantle cell B-cell lymphoma (nodal/systemic with skin involvement) (disorder) | lymphoma | 404150003 |
| Anaplastic lymphoma kinase positive anaplastic large cell lymphoma (disorder) | lymphoma | 1172729004 |
| Anaplastic lymphoma kinase negative anaplastic large cell lymphoma (morphologic abnormality) | lymphoma | 1172729004 |
| Diffuse large B-cell lymphoma associated with chronic inflammation (morphologic abnormality) | lymphoma | 734076008 |
| Anaplastic large cell lymphoma, T/Null cell, primary systemic type (morphologic abnormality) | lymphoma | 413527004 |
| Anaplastic lymphoma kinase positive anaplastic large cell lymphoma (morphologic abnormality) | lymphoma | 734044003 |
| Anaplastic lymphoma kinase negative anaplastic large cell lymphoma (disorder) | lymphoma | 448212009 |
| Anaplastic large cell lymphoma, T/Null cell, primary systemic type (disorder) | lymphoma | 703626001 |
| Nodal peripheral T-cell lymphoma with T follicular helper phenotype (morphologic abnormality) | lymphoma | 784296005 |
| Follicular lymphoma, diffuse follicle center cell sub-type, grade 2 (morphologic abnormality) | lymphoma | 397469009 |
| Lymphomatoid papulosis type C (anaplastic large-cell lymphoma-like) (disorder) | lymphoma | 404105000 |
| Epstein-Barr virus positive diffuse large B-cell lymphoma of elderly (morphologic abnormality) | lymphoma | 716789004 |
| B lymphoblastic leukemia lymphoma with t(9;22)(q34;q11.2); BCR-ABL1 (morphologic abnormality) | lymphoma | 450950002 |
| Epstein-Barr virus positive diffuse large B-cell lymphoma of elderly (disorder) | lymphoma | 716788007 |
| Diffuse large B-cell lymphoma (nodal/systemic with skin involvement) (disorder) | lymphoma | 404148006 |
| B lymphoblastic leukemia lymphoma with t(9:22) (q34;q11.2); BCR-ABL 1 (disorder) | lymphoma | 723889003 |
| B lymphoblastic leukemia lymphoma with hypodiploidy (Hypodiploid ALL) (morphologic abnormality) | lymphoma | 450954006 |
| Hodgkin lymphoma, lymphocyte depletion of lymph nodes of head and neck (disorder) | lymphoma | 1255687003 |
| Primary anaplastic lymphoma kinase fusion oncogene positive non-small cell lung cancer (disorder) | lymphoma | 1259821005 |
| Nodular malignant lymphoma, lymphocytic - intermediate differentiation (disorder) | lymphoma | 302848008 |
| High grade B-cell lymphoma with MYC and BCL2 and/or BCL6 rearrangements (disorder) | lymphoma | 1172704005 |
| High grade B-cell lymphoma with MYC and BCL2 and/or BCL6 rearrangements (morphologic abnormality) | lymphoma | 786909001 |
| Lymphoplasmacytic B-cell lymphoma, nodal/systemic with skin involvement (disorder) | lymphoma | 404149003 |
| Follicular center B-cell lymphoma (nodal/systemic with skin involvement) (disorder) | lymphoma | 404147001 |
| Primary effusion lymphoma due to human immune deficiency virus infection (disorder) | lymphoma | 1153348007 |
| Burkitt lymphoma co-occurrent with human immunodeficiency virus infection (disorder) | lymphoma | 713897006 |
| Precursor T cell lymphoblastic leukemia/lymphoblastic lymphoma (disorder) | lymphoma | 420890002 |
| Classical Hodgkin lymphoma type posttransplant lymphoproliferative disorder (morphologic abnormality) | lymphoma | 782919005 |
| Extranodal marginal zone B-cell lymphoma of mucosa-associated lymphoid tissue (morphologic abnormality) | lymphoma | 397350003 |
| Anaplastic lymphoma kinase positive anaplastic large cell lymphoma (disorder) | lymphoma | 738770003 |
| Extranodal marginal zone B-cell lymphoma of mucosa-associated lymphoid tissue (disorder) | lymphoma | 445269007 |
| B lymphoblastic leukemia lymphoma with t(1;19)(q23;p13.3); E2A-PBX1 (TCF3-PBX1) (morphologic abnormality) | lymphoma | 450956008 |
| B lymphoblastic leukemia lymphoma with t(12;21)(p13;q22); TEL-AML1 (ETV6-RUNX1) (morphologic abnormality) | lymphoma | 450952005 |
| Large B-cell lymphoma arising in human herpesvirus type 8 associated multicentric Castleman disease (morphologic abnormality) | lymphoma | 866098005 |
| Large B-cell lymphoma arising in HHV8-associated multicentric Castleman disease (disorder) | lymphoma | 866098005 |
| B lymphoblastic leukemia lymphoma with t(12;21) (p13;q22); TEL/AML1 (ETV6-RUNX1) (disorder) | lymphoma | 838342003 |
| B lymphoblastic leukemia lymphoma with t(1;19)(Q23;P13.3); E2A-PBX1 (TCF3/PBX1) (disorder) | lymphoma | 838343008 |
| Primary cutaneous CD8 positive aggressive epidermotropic cytotoxic T-cell lymphoma (morphologic abnormality) | lymphoma | 733895005 |
| Primary effusion lymphoma due to human immune deficiency virus infection (disorder) | lymphoma | 714463003 |
| B lymphoblastic leukemia lymphoma with t(5;14)(q31;q32); IL3-IGH (morphologic abnormality) | lymphoma | 450951003 |
| Primary anaplastic lymphoma kinase fusion oncogene negative non-small cell lung cancer (disorder) | lymphoma | 397352006 |
| B lymphoblastic leukemia lymphoma with t(v;11q23); MLL rearranged (morphologic abnormality) | lymphoma | 838342003 |
| Primary anaplastic lymphoma kinase fusion oncogene negative non-small cell lung cancer (disorder) | lymphoma | 1259822003 |
| Primary cutaneous CD8 positive aggressive epidermotropic cytotoxic T-cell lymphoma (disorder) | lymphoma | 765136002 |
| Primary effusion lymphoma co-occurrent with infection caused by Human herpesvirus 8 (disorder) | lymphoma | 714463003 |
| Combined immunodeficiency due to mucosa-associated lymphoid tissue lymphoma translocation gene 1 deficiency (disorder) | lymphoma | 773488000 |
| Diffuse large B-cell lymphoma co-occurrent with chronic inflammation caused by Epstein-Barr virus (disorder) | lymphoma | 724647003 |
| B-cell lymphoma, unclassifiable, with features intermediate between diffuse large B-cell lymphoma and Burkitt lymphoma (morphologic abnormality) | lymphoma | 12341000132100 |
| B-cell lymphoma, unclassifiable, with features intermediate between diffuse large B-cell lymphoma and Hodgkin lymphoma (morphologic abnormality) | lymphoma | 12351000132102 |
| B-cell lymphoma unclassifiable with features intermediate between classical Hodgkin lymphoma and diffuse large B-cell lymphoma (disorder) | lymphoma | 722954005 |
| B-cell lymphoma+862:882, unclassifiable, with features intermediate between diffuse large B-cell lymphoma and Hodgkin lymphoma (morphologic abnormality) | lymphoma | 12351000132102 |
| Malignant neoplasm of breast (disorder) | breast cancer | 254837009 |
| Malignant neoplasm of female breast (disorder) | breast cancer | 372064008 |
| Seen by breast cancer nurse (finding) | breast cancer | 406100007 |
| Triple negative malignant neoplasm of breast (disorder) | breast cancer | 706970001 |
| Locally advanced breast cancer (disorder) | breast cancer | 1082701000112100 |
| Primary malignant neoplasm of breast with axillary lymph node invasion (disorder | breast cancer | 1082901000112103 |
| Lymphoedema following breast cancer (disorder) | breast cancer | 1035841000000108 |
| Hereditary breast and ovarian cancer syndrome (disorder) | breast cancer | 718220008 |
| Re-excision of local recurrence of breast tumour (procedure) | breast cancer | 851181000000109 |
| Wide re-excision of local recurrence of breast tumour (procedure) | breast cancer | 851221000000104 |
| Breast cancer detected by national screening programme (disorder) | breast cancer | 94361000000105 |
| Malignant tumor of lung (disorder) | lung cancer | 363358000 |
| Carcinoma in situ of lung (disorder) | lung cancer | 92649001 |
| Small cell carcinoma of lung (disorder) | lung cancer | 254632001 |
| Metastatic malignant neoplasm to lung (disorder) | lung cancer | 94391008 |
| Non-small cell lung cancer (disorder) | lung cancer | 254637007 |
| Metastatic malignant neoplasm to left lung (disorder) | lung cancer | 353741000119106 |
| Metastatic malignant neoplasm to right lung (disorder) | lung cancer | 353561000119103 |
| Primary non-small cell lung cancer (disorder) | lung cancer | 1259727001 |
| Squamous non-small cell lung cancer (disorder) | lung cancer | 723301009 |
| History of cancer metastatic to lung (situation) | lung cancer | 1098961000119105 |
| Non-small cell lung cancer, negative for epidermal growth factor receptor expression (disorder) | lung cancer | 427038005 |
| Non-small cell lung cancer, positive for epidermal growth factor receptor expression (disorder) | lung cancer | 426964009 |
| Non-small cell lung carcinoma with neuregulin 1 gene fusion (disorder) | lung cancer | 1141627001 |
| Primary squamous non-small cell lung cancer (disorder) | lung cancer | 1259754003 |
| History of non-small cell malignant neoplasm of lung (situation) | lung cancer | 429226001 |
| Primary non-small cell lung cancer (disorder) | lung cancer | 1259727001 |
| Squamous non-small cell lung cancer (disorder) | lung cancer | 723301009 |
| Primary non-small cell lung cancer, positive for epidermal growth factor receptor expression (disorder) | lung cancer | 1259776007 |
| Non-small cell adenocarcinoma of lung (disorder) | lung cancer | 1255725002 |
| Primary non-small cell lung carcinoma with neuregulin 1 gene fusion (disorder) | lung cancer | 1259686004 |
| Primary squamous non-small cell lung cancer (disorder) | lung cancer | 1259754003 |
| Non-small cell adenocarcinoma of lung (disorder) | lung cancer | 1255725002 |
| Primary squamous non-small cell lung cancer (disorder) | lung cancer | 1259754003 |
| Reactive oxygen species 1 positive non-small cell lung cancer (disorder) | lung cancer | 722425009 |
| Reactive oxygen species 1 negative non-small cell lung cancer (disorder) | lung cancer | 830060005 |
| Anaplastic lymphoma kinase fusion oncogene positive non-small cell lung cancer (disorder) | lung cancer | 830151004 |
| Anaplastic lymphoma kinase fusion oncogene negative non-small cell lung cancer (disorder) | lung cancer | 830055006 |
| Non-small cell lung cancer, negative for epidermal growth factor receptor expression (disorder) | lung cancer | 427038005 |
| Non-small cell lung cancer, positive for epidermal growth factor receptor expression (disorder) | lung cancer | 426964009 |
| Primary anaplastic lymphoma kinase fusion oncogene negative non-small cell lung cancer (disorder) | lung cancer | 1259822003 |
| Primary anaplastic lymphoma kinase fusion oncogene positive non-small cell lung cancer (disorder) | lung cancer | 1259821005 |
| Non-small cell lung cancer with mutation in epidermal growth factor receptor (disorder) | lung cancer | 703228009 |
| Primary non-small cell lung cancer, positive for epidermal growth factor receptor expression (disorder) | lung cancer | 1259776007 |
| Primary non-small cell lung cancer negative for epidermal growth factor receptor expression (disorder) | lung cancer | 1259768005 |
| Anaplastic lymphoma kinase fusion oncogene positive non-small cell lung cancer (disorder) | lung cancer | 830151004 |
| Anaplastic lymphoma kinase fusion oncogene negative non-small cell lung cancer (disorder) | lung cancer | 830055006 |
| Non-small cell lung cancer without mutation in epidermal growth factor receptor (disorder) | lung cancer | 703230006 |
| Primary non-small cell lung cancer with mutation in epidermal growth factor receptor (disorder) | lung cancer | 1259760003 |
| Non-small cell lung cancer, positive for epidermal growth factor receptor expression (disorder) | lung cancer | 426964009 |
| Anaplastic lymphoma kinase fusion oncogene negative non-small cell lung cancer (disorder) | lung cancer | 830055006 |
| Non-small cell lung cancer, negative for epidermal growth factor receptor expression (disorder) | lung cancer | 427038005 |
| Primary anaplastic lymphoma kinase fusion oncogene negative non-small cell lung cancer (disorder) | lung cancer | 1259822003 |
| Primary anaplastic lymphoma kinase fusion oncogene positive non-small cell lung cancer (disorder) | lung cancer | 1259821005 |
| Non-small cell lung cancer with mutation in epidermal growth factor receptor (disorder) | lung cancer | 703228009 |
| Primary non-small cell lung cancer without mutation in epidermal growth factor receptor (disorder) | lung cancer | 1259761004 |
| Non-small cell lung cancer without mutation in epidermal growth factor receptor (disorder) | lung cancer | 703230006 |
| Primary non-small cell lung cancer negative for epidermal growth factor receptor expression (disorder) | lung cancer | 1259768005 |
| Primary anaplastic lymphoma kinase fusion oncogene negative non-small cell lung cancer (disorder) | lung cancer | 1259822003 |
| Primary non-small cell lung cancer, positive for epidermal growth factor receptor expression (disorder) | lung cancer | 1259776007 |
| Primary anaplastic lymphoma kinase fusion oncogene positive non-small cell lung cancer (disorder) | lung cancer | 1259821005 |
| Non-small cell lung cancer, negative for epidermal growth factor receptor expression (disorder) | lung cancer | 427038005 |
| Non-small cell lung cancer, positive for epidermal growth factor receptor expression (disorder) | lung cancer | 426964009 |
| Primary non-small cell lung cancer with mutation in epidermal growth factor receptor (disorder) | lung cancer | 1259760003 |
| Primary anaplastic lymphoma kinase fusion oncogene positive non-small cell lung cancer (disorder) | lung cancer | 1259821005 |
| Primary anaplastic lymphoma kinase fusion oncogene negative non-small cell lung cancer (disorder) | lung cancer | 1259822003 |
| Primary non-small cell lung cancer without mutation in epidermal growth factor receptor (disorder) | lung cancer | 1259761004 |
| Primary non-small cell lung cancer negative for epidermal growth factor receptor expression (disorder) | lung cancer | 1259768005 |
| Primary non-small cell lung cancer, positive for epidermal growth factor receptor expression (disorder) | lung cancer | 1259776007 |
| Carcinoma of prostate (disorder) | prostate cancer | 254900004 |
| Malignant tumor of prostate (disorder) | prostate cancer | 399068003 |
| History of malignant neoplasm of prostate (situation) | prostate cancer | 428262008 |
| Primary carcinoma of prostate (disorder) | prostate cancer | 1259388006 |
| Familial malignant neoplasm of prostate (disorder) | prostate cancer | 715412008 |
| Carcinoma in situ of prostate (disorder) | prostate cancer | 92691004 |
| Metastasis from malignant tumor of prostate (disorder) | prostate cancer | 314994000 |
| Prostate cancer care review (procedure) | prostate cancer | 720007002 |
| Metastatic malignant neoplasm to prostate (disorder) | prostate cancer | 94503003 |
| Hormone sensitive prostate cancer (disorder) | prostate cancer | 722103009 |
| Primary malignant neoplasm of prostate metastatic to bone (disorder) | prostate cancer | 712849003 |
| Hormone refractory prostate cancer (disorder) | prostate cancer | 427492003 |
| Active surveillance of prostate cancer (regime/therapy) | prostate cancer | 712837004 |
| Metastasis from malignant tumor of prostate (disorder) | prostate cancer | 314994000 |
| Obstructive nephropathy due to carcinoma of prostate (disorder) | prostate cancer | 722081007 |
| Malignant neoplasm of uterus (disorder) | uterine cancer | 371973000 |
| Malignant tumor of cervix (disorder) | uterine cancer | 363354003 |
| History of malignant neoplasm of uterine body (situation) | uterine cancer | 428941002 |
| Carcinoma of uterine cervix, invasive (disorder) | uterine cancer | 423973006 |
| History of malignant neoplasm of uterine adnexa (situation) | uterine cancer | 428944005 |
| Carcinoma in situ of uterus (disorder) | uterine cancer | 92788005 |
| Metastatic malignant neoplasm to uterus (disorder) | uterine cancer | 94665001 |
| Malignant neoplasm of broad ligament of uterus (disorder) | uterine cancer | 449259009 |
| Metastatic malignant neoplasm to cervix uteri (disorder) | uterine cancer | 188469005 |
| History of malignant neoplasm of cervix (situation) | uterine cancer | 429484003 |
| Malignant neoplasm of colon and/or rectum (disorder) | colorectal cancer | 781382000 |
| Microsatellite instability-high colorectal cancer (disorder) | colorectal cancer | 737058005 |
| Metastasis from malignant neoplasm of colon and/or rectum (disorder) | colorectal cancer | 1217692004 |
| History of malignant neoplasm of colon and/or rectum (situation) | colorectal cancer | 51271000112100 |
| Familial colorectal cancer type X (disorder) | colorectal cancer | 1197359006 |
| Hereditary nonpolyposis colon cancer (disorder) | colorectal cancer | 315058005 |
| Stool DNA-based colorectal cancer screening positive (finding) | colorectal cancer | 708699002 |
| Human epidermal growth factor 2 expressing colon and/or rectum malignant neoplasm (disorder) | colorectal cancer | 1217010007 |
| Malignant tumor of rectum (disorder) | colorectal cancer | 363351006 |
| Malignant neoplasm of colon (disorder) | colorectal cancer | 363406005 |
| Malignant tumor of sigmoid colon (disorder) | colorectal cancer | 363410008 |
| Carcinoma of colon, stage I (finding) | colorectal cancer | 425213009 |
| Carcinoma of colon, stage IV (finding) | colorectal cancer | 422985007 |
| Carcinoma of colon, stage II (finding) | colorectal cancer | 422581008 |
| Carcinoma of colon, stage III (finding) | colorectal cancer | 422375001 |
| Malignant tumor of ascending colon (disorder) | colorectal cancer | 363412000 |
| Carcinoma in situ of colon (disorder) | colorectal cancer | 92568009 |
| Malignant tumor of descending colon (disorder) | colorectal cancer | 363409003 |
| Malignant tumor of transverse colon (disorder) | colorectal cancer | 363408006 |
| Malignant tumor of rectosigmoid junction (disorder) | colorectal cancer | 363414004 |
| Malignant tumor of splenic flexure (disorder) | colorectal cancer | 363413005 |
| Malignant tumor of hepatic flexure (disorder) | colorectal cancer | 363407001 |
| Overlapping malignant neoplasm of colon (disorder) | colorectal cancer | 109838007 |
| Hereditary non-polyposis colon cancer gene mutation positive (finding) | colorectal cancer | 1099611000119109 |
| Malignant tumor of esophagus (disorder) | Upper GI cancer | 363402007 |
| Howel-Evans' syndrome (disorder) | Upper GI cancer | 111030006 |
| Malignant tumor of stomach (disorder) | Upper GI cancer | 363349007 |
| Metastatic malignant neoplasm to stomach (disorder) | Upper GI cancer | 94606003 |
| Carcinoma in situ of fundus of stomach (disorder) | Upper GI cancer | 92598002 |
| Carcinoma in situ of body of stomach (disorder) | Upper GI cancer | 92549006 |
| Adenocarcinoma of stomach (disorder) | Upper GI cancer | 408647009 |
| Carcinoma in situ of cardia of stomach (disorder) | Upper GI cancer | 92560002 |
| Overlapping malignant neoplasm of stomach (disorder) | Upper GI cancer | 109836006 |
| Malignant tumor of duodenum (disorder) | Upper GI cancer | 363403002 |
| Carcinoma in situ of small intestine (disorder) | Upper GI cancer | 92750008 |
| Metastatic malignant neoplasm to small intestine (disorder) | Upper GI cancer | 94580002 |
| Overlapping malignant neoplasm of small intestine (disorder) | Upper GI cancer | 109837002 |
| Carcinoma in situ of ileum (disorder) | Upper GI cancer | 92613002 |

# **Supplementary Table 2: SNOMED CT codes defining prevalent cardiovascular diseases**

# **and major vascular risk factors**

| **Description** | **CVD and VRF type** | **SCTID** |
| --- | --- | --- |
| Malignant hypertension complicating AND/OR reason for care during childbirth (disorder) | Hypertension | 10562009 |
| Benign hypertension (disorder) | Hypertension | 10725009 |
| Eclampsia with pre-existing hypertension in childbirth (disorder) | Hypertension | 10752641000119100 |
| Pre-existing hypertensive heart and chronic kidney disease in mother complicating childbirth (disorder) | Hypertension | 10757401000119100 |
| Multiple drug intolerant hypertension (disorder) | Hypertension | 1078301000112100 |
| Hypertension secondary to renal disease in obstetric context (disorder) | Hypertension | 111438007 |
| Pre-existing hypertensive chronic kidney disease in mother complicating pregnancy (disorder) | Hypertension | 118781000119108 |
| Benign essential hypertension (disorder) | Hypertension | 1201005 |
| Hypertension due to congenital adrenal hyperplasia (disorder) | Hypertension | 1204139007 |
| Secondary hypertension due to renal tubular disorder (disorder) | Hypertension | 1208839002 |
| Secondary hypertension due to congenital heart disorder (disorder) | Hypertension | 1208845005 |
| Renovascular hypertension (disorder) | Hypertension | 123799005 |
| Goldblatt hypertension (disorder) | Hypertension | 123800009 |
| Hypertension concurrent and due to end stage renal disease on dialysis due to type 2 diabetes mellitus (disorder) | Hypertension | 127991000119101 |
| Hypertension concurrent and due to end stage renal disease on dialysis due to type 1 diabetes mellitus (disorder) | Hypertension | 128001000119105 |
| Hypertensive emergency (disorder) | Hypertension | 132721000119104 |
| Hypertension in chronic kidney disease stage 5 due to type 2 diabetes mellitus (disorder) | Hypertension | 140101000119109 |
| Hypertension in chronic kidney disease stage 4 due to type 2 diabetes mellitus (disorder) | Hypertension | 140111000119107 |
| Hypertension in chronic kidney disease stage 3 due to type 2 diabetes mellitus (disorder) | Hypertension | 140121000119100 |
| Hypertension in chronic kidney disease stage 2 due to type 2 diabetes mellitus (disorder) | Hypertension | 140131000119102 |
| Renal sclerosis with hypertension (disorder) | Hypertension | 14973001 |
| Labile systemic arterial hypertension (disorder) | Hypertension | 16229371000119100 |
| Hypertension caused by oral contraceptive pill (disorder) | Hypertension | 169465000 |
| Essential hypertension complicating AND/OR reason for care during childbirth (disorder) | Hypertension | 18416000 |
| Secondary malignant renovascular hypertension (disorder) | Hypertension | 194783001 |
| Secondary benign hypertension (disorder) | Hypertension | 194785008 |
| Hypertension secondary to endocrine disorder (disorder) | Hypertension | 194788005 |
| Hypertension caused by drug (disorder) | Hypertension | 194791005 |
| High-renin essential hypertension (disorder) | Hypertension | 19769006 |
| Benign essential hypertension complicating pregnancy childbirth and the puerperium (disorder) | Hypertension | 198942000 |
| Benign essential hypertension complicating pregnancy childbirth and the puerperium - delivered (disorder) | Hypertension | 198944004 |
| Benign essential hypertension complicating pregnancy childbirth and the puerperium - delivered with postnatal complication (disorder) | Hypertension | 198945003 |
| Benign essential hypertension complicating pregnancy childbirth and the puerperium - not delivered (disorder) | Hypertension | 198946002 |
| Benign essential hypertension complicating pregnancy childbirth and the puerperium with postnatal complication (disorder) | Hypertension | 198947006 |
| Renal hypertension complicating pregnancy childbirth and the puerperium (disorder) | Hypertension | 198949009 |
| Renal hypertension complicating pregnancy childbirth and the puerperium - delivered (disorder) | Hypertension | 198951008 |
| Renal hypertension complicating pregnancy childbirth and the puerperium - delivered with postnatal complication (disorder) | Hypertension | 198952001 |
| Renal hypertension complicating pregnancy childbirth and the puerperium - not delivered (disorder) | Hypertension | 198953006 |
| Renal hypertension complicating pregnancy childbirth and the puerperium with postnatal complication (disorder) | Hypertension | 198954000 |
| Pre-eclampsia or eclampsia with pre-existing hypertension (disorder) | Hypertension | 198997005 |
| Pre-eclampsia or eclampsia with pre-existing hypertension - delivered (disorder) | Hypertension | 198999008 |
| Pre-eclampsia or eclampsia with pre-existing hypertension - delivered with postnatal complication (disorder) | Hypertension | 199000005 |
| Pre-eclampsia or eclampsia with pre-existing hypertension - not delivered (disorder) | Hypertension | 199002002 |
| Pre-eclampsia or eclampsia with pre-existing hypertension with postnatal complication (disorder) | Hypertension | 199003007 |
| Pre-existing hypertension complicating pregnancy childbirth and puerperium (disorder) | Hypertension | 199005000 |
| Pre-existing hypertensive heart and renal disease complicating pregnancy childbirth and the puerperium (disorder) | Hypertension | 199007008 |
| Pre-existing secondary hypertension complicating pregnancy childbirth and puerperium (disorder) | Hypertension | 199008003 |
| Benign essential hypertension complicating AND/OR reason for care during pregnancy (disorder) | Hypertension | 23717007 |
| Malignant hypertension complicating AND/OR reason for care during puerperium (disorder) | Hypertension | 23786008 |
| Chronic hypertension complicating AND/OR reason for care during puerperium (disorder) | Hypertension | 24042004 |
| Hypertension secondary to renal disease complicating AND/OR reason for care during childbirth (disorder) | Hypertension | 26078007 |
| Renal hypertension (disorder) | Hypertension | 28119000 |
| Malignant hypertension complicating AND/OR reason for care during pregnancy (disorder) | Hypertension | 29259002 |
| Pre-existing hypertension complicating AND/OR reason for care during puerperium (disorder) | Hypertension | 31407004 |
| Secondary hypertension (disorder) | Hypertension | 31992008 |
| Pre-existing hypertension complicating AND/OR reason for care during childbirth (disorder) | Hypertension | 34694006 |
| Benign essential hypertension complicating AND/OR reason for care during puerperium (disorder) | Hypertension | 35303009 |
| Hypertension due to compression of renal parenchyma (disorder) | Hypertension | 367821000119106 |
| Labile essential hypertension (disorder) | Hypertension | 371125006 |
| Chronic hypertension complicating AND/OR reason for care during pregnancy (disorder) | Hypertension | 37618003 |
| Hypertensive disorder systemic arterial (disorder) | Hypertension | 38341003 |
| Renal arterial hypertension (disorder) | Hypertension | 39018007 |
| Hypertension secondary to renal disease complicating AND/OR reason for care during puerperium (disorder) | Hypertension | 39727004 |
| Hypertension with albuminuria (disorder) | Hypertension | 397748008 |
| Postpartum pre-existing essential hypertension (disorder) | Hypertension | 40511000119107 |
| Hypertension associated with transplantation (disorder) | Hypertension | 427889009 |
| Hypertension secondary to kidney transplant (disorder) | Hypertension | 428575007 |
| Systolic essential hypertension (disorder) | Hypertension | 429457004 |
| Hypertensive urgency (disorder) | Hypertension | 443482000 |
| Low-renin essential hypertension (disorder) | Hypertension | 46481004 |
| [X]Hypertensive diseases (disorder) | Hypertension | 471521000000108 |
| Diastolic hypertension (disorder) | Hypertension | 48146000 |
| Hypertension secondary to renal disease complicating AND/OR reason for care during pregnancy (disorder) | Hypertension | 48552006 |
| Systolic hypertension (disorder) | Hypertension | 56218007 |
| Parenchymal renal hypertension (disorder) | Hypertension | 57684003 |
| Essential hypertension (disorder) | Hypertension | 59621000 |
| Sustained diastolic hypertension (disorder) | Hypertension | 59720008 |
| Endocrine hypertension (disorder) | Hypertension | 59997006 |
| Benign essential hypertension in obstetric context (disorder) | Hypertension | 63287004 |
| Pre-existing hypertension complicating AND/OR reason for care during pregnancy (disorder) | Hypertension | 65402008 |
| Labile diastolic hypertension (disorder) | Hypertension | 65518004 |
| Pre-eclampsia added to pre-existing hypertension (disorder) | Hypertension | 67359005 |
| Eclampsia added to pre-existing hypertension (disorder) | Hypertension | 69909000 |
| Malignant hypertension (disorder) | Hypertension | 70272006 |
| Hypertension concurrent and due to end stage renal disease on dialysis (disorder) | Hypertension | 704667004 |
| Hypertensive crisis (disorder) | Hypertension | 706882009 |
| Supine hypertension (disorder) | Hypertension | 712832005 |
| Hypertension in chronic kidney disease due to type 2 diabetes mellitus (disorder) | Hypertension | 71421000119105 |
| Hypertension in chronic kidney disease due to type 1 diabetes mellitus (disorder) | Hypertension | 71701000119105 |
| Benign essential hypertension complicating AND/OR reason for care during childbirth (disorder) | Hypertension | 71874008 |
| Essential hypertension in obstetric context (disorder) | Hypertension | 72022006 |
| Brachydactyly and arterial hypertension syndrome (disorder) | Hypertension | 720568003 |
| Benign secondary renovascular hypertension (disorder) | Hypertension | 73410007 |
| Secondary diastolic hypertension (disorder) | Hypertension | 74451002 |
| Diastolic hypertension co-occurrent with systolic hypertension (disorder) | Hypertension | 762463000 |
| Hypertension due to gain-of-function mutation in mineralocorticoid receptor (disorder) | Hypertension | 766937004 |
| Essential hypertension complicating AND/OR reason for care during pregnancy (disorder) | Hypertension | 78808002 |
| Malignant essential hypertension (disorder) | Hypertension | 78975002 |
| Malignant hypertension in obstetric context (disorder) | Hypertension | 81626002 |
| Chronic hypertension complicating AND/OR reason for care during childbirth (disorder) | Hypertension | 8218002 |
| Rebound hypertension (disorder) | Hypertension | 84094009 |
| Stage 1 hypertension (National Institute for Health and Clinical Excellence 2011) (disorder) | Hypertension | 843821000000102 |
| Severe hypertension (National Institute for Health and Clinical Excellence 2011) (disorder) | Hypertension | 843841000000109 |
| Hypertension resistant to drug therapy (disorder) | Hypertension | 845891000000103 |
| Stage 2 hypertension (National Institute for Health and Clinical Excellence 2011) (disorder) | Hypertension | 846371000000103 |
| Pre-existing hypertension in obstetric context (disorder) | Hypertension | 86041002 |
| Nocturnal hypertension (disorder) | Hypertension | 863191000000102 |
| Hypertension due to aortic arch obstruction (disorder) | Hypertension | 871642009 |
| Chronic hypertension in obstetric context (disorder) | Hypertension | 8762007 |
| Malignant secondary hypertension (disorder) | Hypertension | 89242004 |
| Stage 1 hypertension (National Institute for Health and Clinical Excellence 2011) without evidence of end organ damage (disorder) | Hypertension | 908631000000108 |
| Stage 1 hypertension (National Institute for Health and Clinical Excellence 2011) with evidence of end organ damage (disorder) | Hypertension | 908651000000101 |
| Essential hypertension complicating AND/OR reason for care during puerperium (disorder) | Hypertension | 9901000 |
| Chronic kidney disease stage 4 due to hypertension (disorder) | Chronic kidney disease (CKD) stage 3-5 | 129151000119102 |
| Chronic kidney disease stage 5 due to hypertension (disorder) | Chronic kidney disease (CKD) stage 3-5 | 129161000119100 |
| Chronic kidney disease stage 3 due to hypertension (disorder) | Chronic kidney disease (CKD) stage 3-5 | 129171000119106 |
| Hypertension in chronic kidney disease stage 5 due to type 2 diabetes mellitus (disorder) | Chronic kidney disease (CKD) stage 3-5 | 140101000119109 |
| Hypertension in chronic kidney disease stage 4 due to type 2 diabetes mellitus (disorder) | Chronic kidney disease (CKD) stage 3-5 | 140111000119107 |
| Hypertension in chronic kidney disease stage 3 due to type 2 diabetes mellitus (disorder) | Chronic kidney disease (CKD) stage 3-5 | 140121000119100 |
| Malignant hypertensive chronic kidney disease stage 5 (disorder) | Chronic kidney disease (CKD) stage 3-5 | 153851000119106 |
| Chronic kidney disease stage 3 due to benign hypertension (disorder) | Chronic kidney disease (CKD) stage 3-5 | 284991000119104 |
| Chronic kidney disease stage 4 due to benign hypertension (disorder) | Chronic kidney disease (CKD) stage 3-5 | 285001000119105 |
| Chronic kidney disease stage 5 due to benign hypertension (disorder) | Chronic kidney disease (CKD) stage 3-5 | 285011000119108 |
| Malignant hypertensive chronic kidney disease stage 3 (disorder) | Chronic kidney disease (CKD) stage 3-5 | 285871000119106 |
| Malignant hypertensive chronic kidney disease stage 4 (disorder) | Chronic kidney disease (CKD) stage 3-5 | 285881000119109 |
| Chronic kidney disease stage 3 with proteinuria (disorder) | Chronic kidney disease (CKD) stage 3-5 | 324251000000105 |
| Chronic kidney disease stage 3 without proteinuria (disorder) | Chronic kidney disease (CKD) stage 3-5 | 324281000000104 |
| Chronic kidney disease stage 3A with proteinuria (disorder) | Chronic kidney disease (CKD) stage 3-5 | 324311000000101 |
| Chronic kidney disease stage 3A without proteinuria (disorder) | Chronic kidney disease (CKD) stage 3-5 | 324341000000100 |
| Chronic kidney disease stage 3B with proteinuria (disorder) | Chronic kidney disease (CKD) stage 3-5 | 324371000000106 |
| Chronic kidney disease stage 3B without proteinuria (disorder) | Chronic kidney disease (CKD) stage 3-5 | 324411000000105 |
| Chronic kidney disease stage 4 with proteinuria (disorder) | Chronic kidney disease (CKD) stage 3-5 | 324441000000106 |
| Chronic kidney disease stage 4 without proteinuria (disorder) | Chronic kidney disease (CKD) stage 3-5 | 324471000000100 |
| Chronic kidney disease stage 5 with proteinuria (disorder) | Chronic kidney disease (CKD) stage 3-5 | 324501000000107 |
| Chronic kidney disease stage 5 without proteinuria (disorder) | Chronic kidney disease (CKD) stage 3-5 | 324541000000105 |
| Chronic kidney disease stage 3 due to drug induced diabetes mellitus (disorder) | Chronic kidney disease (CKD) stage 3-5 | 368441000119102 |
| Chronic kidney disease stage 4 due to drug induced diabetes mellitus (disorder) | Chronic kidney disease (CKD) stage 3-5 | 368451000119100 |
| Chronic kidney disease stage 5 due to drug induced diabetes mellitus (disorder) | Chronic kidney disease (CKD) stage 3-5 | 368461000119103 |
| Chronic kidney disease stage 4 (disorder) | Chronic kidney disease (CKD) stage 3-5 | 431857002 |
| Chronic kidney disease stage 3 (disorder) | Chronic kidney disease (CKD) stage 3-5 | 433144002 |
| Chronic kidney disease stage 5 (disorder) | Chronic kidney disease (CKD) stage 3-5 | 433146000 |
| Anemia co-occurrent and due to chronic kidney disease stage 3 (disorder) | Chronic kidney disease (CKD) stage 3-5 | 691421000119108 |
| Chronic kidney disease stage 3A (disorder) | Chronic kidney disease (CKD) stage 3-5 | 700378005 |
| Chronic kidney disease stage 3B (disorder) | Chronic kidney disease (CKD) stage 3-5 | 700379002 |
| Chronic kidney disease stage 5 due to type 2 diabetes mellitus (disorder) | Chronic kidney disease (CKD) stage 3-5 | 711000119100 |
| Chronic kidney disease stage 5 on dialysis (disorder) | Chronic kidney disease (CKD) stage 3-5 | 714152005 |
| Chronic kidney disease stage 5 with transplant (disorder) | Chronic kidney disease (CKD) stage 3-5 | 714153000 |
| Chronic kidney disease stage 4 due to type 2 diabetes mellitus (disorder) | Chronic kidney disease (CKD) stage 3-5 | 721000119107 |
| Chronic kidney disease stage 3 due to type 2 diabetes mellitus (disorder) | Chronic kidney disease (CKD) stage 3-5 | 731000119105 |
| Chronic kidney disease stage 3 due to type 1 diabetes mellitus (disorder) | Chronic kidney disease (CKD) stage 3-5 | 90741000119107 |
| Chronic kidney disease stage 4 due to type 1 diabetes mellitus (disorder) | Chronic kidney disease (CKD) stage 3-5 | 90751000119109 |
| Chronic kidney disease stage 5 due to type 1 diabetes mellitus (disorder) | Chronic kidney disease (CKD) stage 3-5 | 90761000119106 |
| Chronic kidney disease with glomerular filtration rate category G3a and albuminuria category A1 (disorder) | Chronic kidney disease (CKD) stage 3-5 | 949881000000106 |
| Chronic kidney disease with glomerular filtration rate category G3a and albuminuria category A2 (disorder) | Chronic kidney disease (CKD) stage 3-5 | 949901000000109 |
| Chronic kidney disease with glomerular filtration rate category G3a and albuminuria category A3 (disorder) | Chronic kidney disease (CKD) stage 3-5 | 949921000000100 |
| Chronic kidney disease with glomerular filtration rate category G3b and albuminuria category A1 (disorder) | Chronic kidney disease (CKD) stage 3-5 | 950061000000103 |
| Chronic kidney disease with glomerular filtration rate category G3b and albuminuria category A2 (disorder) | Chronic kidney disease (CKD) stage 3-5 | 950081000000107 |
| Chronic kidney disease with glomerular filtration rate category G3b and albuminuria category A3 (disorder) | Chronic kidney disease (CKD) stage 3-5 | 950101000000101 |
| Chronic kidney disease with glomerular filtration rate category G4 and albuminuria category A1 (disorder) | Chronic kidney disease (CKD) stage 3-5 | 950181000000106 |
| Chronic kidney disease with glomerular filtration rate category G4 and albuminuria category A2 (disorder) | Chronic kidney disease (CKD) stage 3-5 | 950211000000107 |
| Chronic kidney disease with glomerular filtration rate category G4 and albuminuria category A3 (disorder) | Chronic kidney disease (CKD) stage 3-5 | 950231000000104 |
| Chronic kidney disease with glomerular filtration rate category G5 and albuminuria category A1 (disorder) | Chronic kidney disease (CKD) stage 3-5 | 950251000000106 |
| Chronic kidney disease with glomerular filtration rate category G5 and albuminuria category A2 (disorder) | Chronic kidney disease (CKD) stage 3-5 | 950291000000103 |
| Chronic kidney disease with glomerular filtration rate category G5 and albuminuria category A3 (disorder) | Chronic kidney disease (CKD) stage 3-5 | 950311000000102 |
| Hypertensive heart AND chronic kidney disease stage 5 (disorder) | Chronic kidney disease (CKD) stage 3-5 | 96711000119105 |
| Hypertensive heart AND chronic kidney disease stage 4 (disorder) | Chronic kidney disease (CKD) stage 3-5 | 96721000119103 |
| Hypertensive heart AND chronic kidney disease stage 3 (disorder) | Chronic kidney disease (CKD) stage 3-5 | 96731000119100 |
| Diabetes mellitus without complication (disorder) | Diabetes mellitus | 111552007 |
| Lipoatrophic diabetes mellitus without complication (disorder) | Diabetes mellitus | 112991000000101 |
| Pre-existing malnutrition-related diabetes mellitus in pregnancy (disorder) | Diabetes mellitus | 1142044000 |
| Brittle diabetes mellitus (finding) | Diabetes mellitus | 11530004 |
| Neovascular glaucoma due to diabetes mellitus type 2 (disorder) | Diabetes mellitus | 1196922005 |
| Neovascular glaucoma due to diabetes mellitus type 1 (disorder) | Diabetes mellitus | 1196923000 |
| Ketosis-resistant diabetes mellitus (disorder) | Diabetes mellitus | 1217044000 |
| Fulminant type 1 diabetes mellitus (disorder) | Diabetes mellitus | 1217068008 |
| Neurotrophic keratitis due to diabetes mellitus (disorder) | Diabetes mellitus | 1217674007 |
| Pancreatic agenesis holoprosencephaly syndrome (disorder) | Diabetes mellitus | 1222660008 |
| Lipoatrophic diabetes (disorder) | Diabetes mellitus | 127012008 |
| Disorder of kidney due to diabetes mellitus (disorder) | Diabetes mellitus | 127013003 |
| Peripheral angiopathy due to diabetes mellitus (disorder) | Diabetes mellitus | 127014009 |
| Diabetes mellitus type 2 without retinopathy (disorder) | Diabetes mellitus | 1481000119100 |
| Type I diabetes mellitus with ulcer (disorder) | Diabetes mellitus | 190368000 |
| Type I diabetes mellitus with gangrene (disorder) | Diabetes mellitus | 190369008 |
| Type I diabetes mellitus maturity onset (disorder) | Diabetes mellitus | 190372001 |
| Multiple complications due to type 2 diabetes mellitus (disorder) | Diabetes mellitus | 190388001 |
| Type II diabetes mellitus with ulcer (disorder) | Diabetes mellitus | 190389009 |
| Type II diabetes mellitus with gangrene (disorder) | Diabetes mellitus | 190390000 |
| Neuropathic arthropathy due to diabetes mellitus (disorder) | Diabetes mellitus | 201724008 |
| Mononeuropathy due to diabetes mellitus (disorder) | Diabetes mellitus | 230577008 |
| Insulin treated type 2 diabetes mellitus (disorder) | Diabetes mellitus | 237599002 |
| Secondary endocrine diabetes mellitus (disorder) | Diabetes mellitus | 237601000 |
| Maturity onset diabetes of the young type 2 (disorder) | Diabetes mellitus | 237604008 |
| Photomyoclonus diabetes mellitus deafness nephropathy and cerebral dysfunction (disorder) | Diabetes mellitus | 237612000 |
| Insulin-dependent diabetes mellitus secretory diarrhea syndrome (disorder) | Diabetes mellitus | 237618001 |
| Diabetes-deafness syndrome maternally transmitted (disorder) | Diabetes mellitus | 237619009 |
| Diabetic - poor control (finding) | Diabetes mellitus | 268519009 |
| Brittle type I diabetes mellitus (finding) | Diabetes mellitus | 290002008 |
| Type I diabetes mellitus without complication (disorder) | Diabetes mellitus | 313435000 |
| Type II diabetes mellitus without complication (disorder) | Diabetes mellitus | 313436004 |
| Type I diabetes mellitus with hypoglycemic coma (disorder) | Diabetes mellitus | 314771006 |
| Arthropathy due to type 1 diabetes mellitus (disorder) | Diabetes mellitus | 314893005 |
| Peripheral angiopathy due to type 2 diabetes mellitus (disorder) | Diabetes mellitus | 314902007 |
| Arthropathy due to type 2 diabetes mellitus (disorder) | Diabetes mellitus | 314903002 |
| Type II diabetes mellitus with neuropathic arthropathy (disorder) | Diabetes mellitus | 314904008 |
| Maternally inherited diabetes mellitus (disorder) | Diabetes mellitus | 335621000000101 |
| Mild nonproliferative retinopathy due to secondary diabetes mellitus (disorder) | Diabetes mellitus | 368711000119106 |
| Diabetes mellitus with multiple complications (disorder) | Diabetes mellitus | 385041000000108 |
| Hyperosmolar non-ketotic state due to type 2 diabetes mellitus (disorder) | Diabetes mellitus | 395204000 |
| Type 1 diabetes mellitus with persistent microalbuminuria (disorder) | Diabetes mellitus | 401110002 |
| Diabetes mellitus caused by non-steroid drugs (disorder) | Diabetes mellitus | 408540003 |
| Diabetes mellitus caused by non-steroid drugs without complication (disorder) | Diabetes mellitus | 413183008 |
| Ketoacidosis due to type 1 diabetes mellitus (disorder) | Diabetes mellitus | 420270002 |
| Gangrene due to type 1 diabetes mellitus (disorder) | Diabetes mellitus | 420825003 |
| Disorder due to type 1 diabetes mellitus (disorder) | Diabetes mellitus | 420868002 |
| Ketoacidotic coma due to type 1 diabetes mellitus (disorder) | Diabetes mellitus | 421075007 |
| Persistent microalbuminuria due to type 1 diabetes mellitus (disorder) | Diabetes mellitus | 421305000 |
| Hypoglycemic coma due to type 1 diabetes mellitus | Diabetes mellitus | 421437000 |
| Gangrene due to type 2 diabetes mellitus (disorder) | Diabetes mellitus | 421631007 |
| Ketoacidosis due to type 2 diabetes mellitus (disorder) | Diabetes mellitus | 421750000 |
| Ketoacidotic coma due to type 2 diabetes mellitus (disorder) | Diabetes mellitus | 421847006 |
| Disorder due to type 2 diabetes mellitus (disorder) | Diabetes mellitus | 422014003 |
| Multiple complications due to type 1 diabetes mellitus (disorder) | Diabetes mellitus | 422228004 |
| Diabetes mellitus co-occurrent and due to cystic fibrosis (disorder) | Diabetes mellitus | 426705001 |
| Latent autoimmune diabetes mellitus in adult (disorder) | Diabetes mellitus | 426875007 |
| Diabetes mellitus due to cystic fibrosis (disorder) | Diabetes mellitus | 427089005 |
| Diabetes mellitus type 2 (disorder) | Diabetes mellitus | 44054006 |
| Type II diabetes mellitus uncontrolled (finding) | Diabetes mellitus | 443694000 |
| Type I diabetes mellitus uncontrolled (finding) | Diabetes mellitus | 444073006 |
| Brittle type II diabetes mellitus (finding) | Diabetes mellitus | 445353002 |
| Diabetes mellitus type 1 (disorder) | Diabetes mellitus | 46635009 |
| Retinopathy due to diabetes mellitus (disorder) | Diabetes mellitus | 4855003 |
| Polyneuropathy due to diabetes mellitus (disorder) | Diabetes mellitus | 49455004 |
| Diabetes mellitus associated with pancreatic disease (disorder) | Diabetes mellitus | 51002006 |
| Drug-induced diabetes mellitus (disorder) | Diabetes mellitus | 5368009 |
| Diabetes mellitus associated with genetic syndrome (disorder) | Diabetes mellitus | 5969009 |
| MODY - maturity onset diabetes of young | Diabetes mellitus | 609561005 |
| Maturity onset diabetes of the young type 1 (disorder) | Diabetes mellitus | 609562003 |
| Maturity-onset diabetes of the young type 5 (disorder) | Diabetes mellitus | 609572000 |
| Diabetes mellitus in remission (disorder) | Diabetes mellitus | 703136005 |
| Type I diabetes mellitus in remission (disorder) | Diabetes mellitus | 703137001 |
| Type II diabetes mellitus in remission (disorder) | Diabetes mellitus | 703138006 |
| Diabetes mellitus AND insipidus with optic atrophy AND deafness (disorder) | Diabetes mellitus | 70694009 |
| Diabetes mellitus (disorder) | Diabetes mellitus | 73211009 |
| Diabetes mellitus caused by chemical (disorder) | Diabetes mellitus | 737212004 |
| Disorder of eye due to type 1 diabetes mellitus (disorder) | Diabetes mellitus | 739681000 |
| Diabetes mellitus caused by insulin receptor antibodies (disorder) | Diabetes mellitus | 75682002 |
| Cataract of right eye due to diabetes mellitus (disorder) | Diabetes mellitus | 768792007 |
| Cataract of left eye due to diabetes mellitus (disorder) | Diabetes mellitus | 768793002 |
| Cataract of bilateral eyes due to diabetes mellitus (disorder) | Diabetes mellitus | 768794008 |
| Diabetes hypogonadism deafness intellectual disability syndrome (disorder) | Diabetes mellitus | 816067005 |
| Rubeosis iridis due to type 1 diabetes mellitus (disorder) | Diabetes mellitus | 82581000119105 |
| Secondary diabetes mellitus (disorder) | Diabetes mellitus | 8801005 |
| Acute infarction of papillary muscle (disorder) | Coronary heart disease (CHD) | 10273003 |
| Coronary arteriosclerosis in patient with history of previous myocardial infarction (situation) | Coronary heart disease (CHD) | 103011000119106 |
| Septal infarction by electrocardiogram (finding) | Coronary heart disease (CHD) | 1077002 |
| Postoperative nontransmural myocardial infarction (disorder) | Coronary heart disease (CHD) | 1089431000000100 |
| Postoperative transmural myocardial infarction (disorder) | Coronary heart disease (CHD) | 1089441000000100 |
| Acute nontransmural myocardial infarction (disorder) | Coronary heart disease (CHD) | 1089451000000100 |
| Acute transmural myocardial infarction (disorder) | Coronary heart disease (CHD) | 1089471000000100 |
| Myocardial ischemia during surgery (disorder) | Coronary heart disease (CHD) | 10971000087107 |
| Coronary arteriosclerosis after percutaneous coronary angioplasty (disorder) | Coronary heart disease (CHD) | 11018701000119100 |
| Pericardial effusion following myocardial infarction (disorder) | Coronary heart disease (CHD) | 1163420005 |
| Postoperative acute myocardial infarction (disorder) | Coronary heart disease (CHD) | 1163440003 |
| Chronic total occlusion of coronary artery (disorder) | Coronary heart disease (CHD) | 117051000119103 |
| Acute inferior non-ST segment elevation myocardial infarction of right ventricle (disorder) | Coronary heart disease (CHD) | 1204151009 |
| Acute inferior non-ST segment elevation myocardial infarction (disorder) | Coronary heart disease (CHD) | 1204152002 |
| Acute anterior non-ST segment elevation myocardial infarction with right ventricular involvement (disorder) | Coronary heart disease (CHD) | 1204154001 |
| Acute anterior non-ST segment elevation myocardial infarction (disorder) | Coronary heart disease (CHD) | 1204155000 |
| Acute non-ST segment elevation myocardial infarction of right ventricle (disorder) | Coronary heart disease (CHD) | 1204222000 |
| Ventricular thrombus following acute myocardial infarction (disorder) | Coronary heart disease (CHD) | 1208867006 |
| Subsequent anterior non-ST segment elevation myocardial infarction (disorder) | Coronary heart disease (CHD) | 1208872002 |
| Subsequent inferior non-ST segment elevation myocardial infarction (disorder) | Coronary heart disease (CHD) | 1208873007 |
| Hypercyanotic spell due to congenital heart disease (finding) | Coronary heart disease (CHD) | 1222625002 |
| Acute ST segment elevation myocardial infarction of inferolateral wall (disorder) | Coronary heart disease (CHD) | 12238111000119100 |
| Acute ST segment elevation myocardial infarction of inferoposterior wall (disorder) | Coronary heart disease (CHD) | 12238151000119100 |
| Left coronary artery occlusion (disorder) | Coronary heart disease (CHD) | 123641001 |
| Right coronary artery occlusion (disorder) | Coronary heart disease (CHD) | 123642008 |
| Postoperative myocardial infarction (disorder) | Coronary heart disease (CHD) | 129574000 |
| History of placement of stent in coronary artery bypass graft (situation) | Coronary heart disease (CHD) | 130541000119100 |
| Coronary arteriosclerosis following coronary artery bypass graft (disorder) | Coronary heart disease (CHD) | 139011000119104 |
| Acute ST segment elevation myocardial infarction of posterolateral wall (disorder) | Coronary heart disease (CHD) | 15712841000119100 |
| Acute ST segment elevation myocardial infarction of anterolateral wall (disorder) | Coronary heart disease (CHD) | 15712881000119100 |
| Acute ST segment elevation myocardial infarction of lateral wall (disorder) | Coronary heart disease (CHD) | 15712921000119100 |
| Acute ST segment elevation myocardial infarction of anteroseptal wall (disorder) | Coronary heart disease (CHD) | 15712961000119100 |
| Acute ST segment elevation myocardial infarction of posterior wall (disorder) | Coronary heart disease (CHD) | 15713041000119100 |
| Acute ST segment elevation myocardial infarction due to left coronary artery occlusion (disorder) | Coronary heart disease (CHD) | 15713081000119100 |
| Acute ST segment elevation myocardial infarction due to right coronary artery occlusion (disorder) | Coronary heart disease (CHD) | 15713121000119100 |
| Acute ST segment elevation myocardial infarction of septum (disorder) | Coronary heart disease (CHD) | 15713161000119100 |
| Acute ST segment elevation myocardial infarction of posterobasal wall (disorder) | Coronary heart disease (CHD) | 15713201000119100 |
| Unstable angina co-occurrent and due to coronary arteriosclerosis (disorder) | Coronary heart disease (CHD) | 15960061000119100 |
| Angina co-occurrent and due to coronary arteriosclerosis (disorder) | Coronary heart disease (CHD) | 15960141000119100 |
| Unstable angina due to arteriosclerosis of coronary artery bypass graft of transplanted heart (disorder) | Coronary heart disease (CHD) | 15960341000119100 |
| Angina co-occurrent and due to arteriosclerosis of coronary artery bypass graft (disorder) | Coronary heart disease (CHD) | 15960381000119100 |
| Unstable angina due to arteriosclerosis of autologous arterial coronary artery bypass graft (disorder) | Coronary heart disease (CHD) | 15960461000119100 |
| Unstable angina due to arteriosclerosis of autologous vein coronary artery bypass graft (disorder) | Coronary heart disease (CHD) | 15960541000119100 |
| Angina co-occurrent and due to arteriosclerosis of autologous vein coronary artery bypass graft (disorder) | Coronary heart disease (CHD) | 15960581000119100 |
| Unstable angina co-occurrent and due to arteriosclerosis of coronary artery bypass graft (disorder) | Coronary heart disease (CHD) | 15960661000119100 |
| Mural thrombus of left ventricle following acute myocardial infarction (disorder) | Coronary heart disease (CHD) | 15960981000119100 |
| Acute myocardial infarction of posterolateral wall (disorder) | Coronary heart disease (CHD) | 15990001 |
| History of myocardial infarct at age less than sixty (situation) | Coronary heart disease (CHD) | 161502000 |
| History of myocardial infarct at age greater than sixty (situation) | Coronary heart disease (CHD) | 161503005 |
| Coronary arteriosclerosis in native artery (disorder) | Coronary heart disease (CHD) | 1641000119107 |
| Supraventricular tachycardia following acute myocardial infarction (disorder) | Coronary heart disease (CHD) | 16415081000119100 |
| Myocardial infarction due to demand ischemia | Coronary heart disease (CHD) | 16837681000119100 |
| Coronary artery disease due to type 2 diabetes mellitus (disorder) | Coronary heart disease (CHD) | 16891151000119100 |
| Coronary artery disease due to type 1 diabetes mellitus (disorder) | Coronary heart disease (CHD) | 16891191000119100 |
| Acute myocardial infarction due to left coronary artery occlusion (disorder) | Coronary heart disease (CHD) | 17531000119105 |
| Old myocardial infarction (disorder) | Coronary heart disease (CHD) | 1755008 |
| Status anginosus (disorder) | Coronary heart disease (CHD) | 19057007 |
| True posterior myocardial infarction (disorder) | Coronary heart disease (CHD) | 194802003 |
| Acute atrial infarction (disorder) | Coronary heart disease (CHD) | 194809007 |
| Coronary thrombosis not resulting in myocardial infarction (disorder) | Coronary heart disease (CHD) | 194821006 |
| Acute coronary insufficiency (disorder) | Coronary heart disease (CHD) | 194823009 |
| Angina (disorder) | Coronary heart disease (CHD) | 194828000 |
| Single coronary vessel disease (disorder) | Coronary heart disease (CHD) | 194842008 |
| Double coronary vessel disease (disorder) | Coronary heart disease (CHD) | 194843003 |
| Generalized ischemic myocardial dysfunction (disorder) | Coronary heart disease (CHD) | 194849004 |
| Subsequent myocardial infarction (disorder) | Coronary heart disease (CHD) | 194856005 |
| Subsequent myocardial infarction of anterior wall (disorder) | Coronary heart disease (CHD) | 194857001 |
| Subsequent myocardial infarction of inferior wall (disorder) | Coronary heart disease (CHD) | 194858006 |
| Certain current complications following acute myocardial infarction (disorder) | Coronary heart disease (CHD) | 194861007 |
| Hemopericardium due to and following acute myocardial infarction (disorder) | Coronary heart disease (CHD) | 194862000 |
| Atrial septal defect due to and following acute myocardial infarction (disorder) | Coronary heart disease (CHD) | 194863005 |
| Rupture of cardiac wall without hemopericardium as current complication following acute myocardial infarction (disorder) | Coronary heart disease (CHD) | 194865003 |
| Rupture of chordae tendinae due to and following acute myocardial infarction (disorder) | Coronary heart disease (CHD) | 194866002 |
| Rupture of papillary muscle as current complication following acute myocardial infarction (disorder) | Coronary heart disease (CHD) | 194867006 |
| Thrombosis of atrium auricular appendage and ventricle due to and following acute myocardial infarction (disorder) | Coronary heart disease (CHD) | 194868001 |
| Syncope anginosa (disorder) | Coronary heart disease (CHD) | 21470009 |
| MI - myocardial infarction | Coronary heart disease (CHD) | 22298006 |
| Ischemic chest pain (finding) | Coronary heart disease (CHD) | 225566008 |
| Acute myocardial infarction due to right coronary artery occlusion (disorder) | Coronary heart disease (CHD) | 23311000119105 |
| Triple vessel disease of the heart (disorder) | Coronary heart disease (CHD) | 233817007 |
| Stable angina (disorder) | Coronary heart disease (CHD) | 233819005 |
| New onset angina (disorder) | Coronary heart disease (CHD) | 233821000 |
| Silent myocardial ischemia (disorder) | Coronary heart disease (CHD) | 233823002 |
| Acute Q wave infarction - anteroseptal (disorder) | Coronary heart disease (CHD) | 233825009 |
| Acute non-Q wave infarction - anteroseptal (disorder) | Coronary heart disease (CHD) | 233826005 |
| Acute Q wave infarction - anterolateral (disorder) | Coronary heart disease (CHD) | 233827001 |
| Acute non-Q wave infarction - anterolateral (disorder) | Coronary heart disease (CHD) | 233828006 |
| Acute Q wave infarction - inferior (disorder) | Coronary heart disease (CHD) | 233829003 |
| Acute non-Q wave infarction - inferior (disorder) | Coronary heart disease (CHD) | 233830008 |
| Acute Q wave infarction - inferolateral (disorder) | Coronary heart disease (CHD) | 233831007 |
| Acute non-Q wave infarction - inferolateral (disorder) | Coronary heart disease (CHD) | 233832000 |
| Acute Q wave infarction - lateral (disorder) | Coronary heart disease (CHD) | 233833005 |
| Acute non-Q wave infarction - lateral (disorder) | Coronary heart disease (CHD) | 233834004 |
| Acute widespread myocardial infarction (disorder) | Coronary heart disease (CHD) | 233835003 |
| Acute Q wave infarction - widespread (disorder) | Coronary heart disease (CHD) | 233836002 |
| Acute non-Q wave infarction - widespread (disorder) | Coronary heart disease (CHD) | 233837006 |
| Acute posterior myocardial infarction (disorder) | Coronary heart disease (CHD) | 233838001 |
| Old anterior myocardial infarction (disorder) | Coronary heart disease (CHD) | 233839009 |
| Old inferior myocardial infarction (disorder) | Coronary heart disease (CHD) | 233840006 |
| Old lateral myocardial infarction (disorder) | Coronary heart disease (CHD) | 233841005 |
| Old posterior myocardial infarction (disorder) | Coronary heart disease (CHD) | 233842003 |
| Silent myocardial infarction (disorder) | Coronary heart disease (CHD) | 233843008 |
| Accelerated coronary artery disease in transplanted heart (disorder) | Coronary heart disease (CHD) | 233844002 |
| Post-infarction ventricular septal defect (disorder) | Coronary heart disease (CHD) | 233846000 |
| Cardiac rupture due to and following acute myocardial infarction (disorder) | Coronary heart disease (CHD) | 233847009 |
| Post-infarction pericarditis (disorder) | Coronary heart disease (CHD) | 233885007 |
| Coronary artery stenosis (disorder) | Coronary heart disease (CHD) | 233970002 |
| Coronary graft stenosis (disorder) | Coronary heart disease (CHD) | 251024009 |
| Impending infarction (disorder) | Coronary heart disease (CHD) | 25106000 |
| Ischemic myocardial dysfunction (disorder) | Coronary heart disease (CHD) | 281091000 |
| Stunned myocardium (disorder) | Coronary heart disease (CHD) | 281092007 |
| Hibernating myocardium (disorder) | Coronary heart disease (CHD) | 281093002 |
| Acute myocardial infarction of basal-lateral wall (disorder) | Coronary heart disease (CHD) | 282006 |
| Arteriosclerosis of autologous arterial coronary artery bypass graft (disorder) | Coronary heart disease (CHD) | 285141000119106 |
| Arteriosclerosis of autologous coronary artery bypass graft (disorder) | Coronary heart disease (CHD) | 285151000119108 |
| History of acute ST segment elevation myocardial infarction (situation) | Coronary heart disease (CHD) | 285721000119104 |
| Mechanical breakdown of coronary artery bypass graft (disorder) | Coronary heart disease (CHD) | 285951000119105 |
| Acute ST segment elevation myocardial infarction involving left anterior descending coronary artery (disorder) | Coronary heart disease (CHD) | 285981000119103 |
| Acute ST segment elevation myocardial infarction involving left main coronary artery (disorder) | Coronary heart disease (CHD) | 285991000119100 |
| Exercise-induced angina (disorder) | Coronary heart disease (CHD) | 300995000 |
| Rupture of ventricle due to acute myocardial infarction (disorder) | Coronary heart disease (CHD) | 30277009 |
| Acute Q wave myocardial infarction (disorder) | Coronary heart disease (CHD) | 304914007 |
| Acute non-Q wave infarction (disorder) | Coronary heart disease (CHD) | 307140009 |
| History of myocardial infarction in last year (situation) | Coronary heart disease (CHD) | 308065005 |
| Postoperative transmural myocardial infarction of anterior wall (disorder) | Coronary heart disease (CHD) | 311792005 |
| Postoperative transmural myocardial infarction of inferior wall (disorder) | Coronary heart disease (CHD) | 311793000 |
| Postoperative subendocardial myocardial infarction (disorder) | Coronary heart disease (CHD) | 311796008 |
| Post infarct angina (disorder) | Coronary heart disease (CHD) | 314116003 |
| Non-Q wave myocardial infarction (disorder) | Coronary heart disease (CHD) | 314207007 |
| Refractory angina (disorder) | Coronary heart disease (CHD) | 315025001 |
| Transient myocardial ischemia (disorder) | Coronary heart disease (CHD) | 315026000 |
| Diabetes mellitus insulin-glucose infusion in acute myocardial infarction (procedure) | Coronary heart disease (CHD) | 315287002 |
| Asymptomatic coronary heart disease (disorder) | Coronary heart disease (CHD) | 315348000 |
| Past myocardial infarction diagnosed on electrocardiogram AND/OR other special investigation but currently presenting no symptoms (disorder) | Coronary heart disease (CHD) | 32574007 |
| Sequela of cardioembolic stroke (disorder) | Coronary heart disease (CHD) | 33301000119105 |
| Nocturnal angina (disorder) | Coronary heart disease (CHD) | 35928006 |
| Myocardial infarction with complication (disorder) | Coronary heart disease (CHD) | 371068009 |
| Multi vessel coronary artery disease (disorder) | Coronary heart disease (CHD) | 371803003 |
| Left main coronary artery disease (disorder) | Coronary heart disease (CHD) | 371804009 |
| Significant coronary bypass graft disease (disorder) | Coronary heart disease (CHD) | 371805005 |
| Progressive angina (disorder) | Coronary heart disease (CHD) | 371806006 |
| Atypical angina (disorder) | Coronary heart disease (CHD) | 371807002 |
| Recurrent angina status post percutaneous transluminal coronary angioplasty (disorder) | Coronary heart disease (CHD) | 371808007 |
| Recurrent angina following placement of coronary artery stent (disorder) | Coronary heart disease (CHD) | 371809004 |
| Recurrent angina status post coronary artery bypass graft (disorder) | Coronary heart disease (CHD) | 371810009 |
| Recurrent angina status post rotational atherectomy (disorder) | Coronary heart disease (CHD) | 371811008 |
| Recurrent angina status post directional coronary atherectomy (disorder) | Coronary heart disease (CHD) | 371812001 |
| History of myocardial infarction due to atherothrombotic coronary artery disease (situation) | Coronary heart disease (CHD) | 387785661000119000 |
| Acute coronary syndrome (disorder) | Coronary heart disease (CHD) | 394659003 |
| First myocardial infarction (disorder) | Coronary heart disease (CHD) | 394710008 |
| Coronary artery thrombosis (disorder) | Coronary heart disease (CHD) | 398274000 |
| History of myocardial infarction (situation) | Coronary heart disease (CHD) | 399211009 |
| History of coronary artery bypass grafting (situation) | Coronary heart disease (CHD) | 399261000 |
| Acute ST segment elevation myocardial infarction (disorder) | Coronary heart disease (CHD) | 401303003 |
| Acute non-ST segment elevation myocardial infarction (disorder) | Coronary heart disease (CHD) | 401314000 |
| Coronary artery bypass graft occlusion (disorder) | Coronary heart disease (CHD) | 408546009 |
| Angina class II (disorder) | Coronary heart disease (CHD) | 41334000 |
| Acute ischemic heart disease (disorder) | Coronary heart disease (CHD) | 413439005 |
| Acute myocardial ischemia (disorder) | Coronary heart disease (CHD) | 413444003 |
| Chronic ischemic heart disease (disorder) | Coronary heart disease (CHD) | 413838009 |
| Chronic myocardial ischemia (disorder) | Coronary heart disease (CHD) | 413844008 |
| Disorder of coronary artery (disorder) | Coronary heart disease (CHD) | 414024009 |
| Ischemic heart disease (disorder) | Coronary heart disease (CHD) | 414545008 |
| Myocardial ischemia (disorder) | Coronary heart disease (CHD) | 414795007 |
| Myocardial infarction in recovery phase (disorder) | Coronary heart disease (CHD) | 418044006 |
| Obliterative coronary artery disease (disorder) | Coronary heart disease (CHD) | 420006002 |
| Microinfarct of heart (disorder) | Coronary heart disease (CHD) | 42531007 |
| Dilated cardiomyopathy of ischemic origin | Coronary heart disease (CHD) | 426856002 |
| Coronary arteriosclerosis caused by radiation (disorder) | Coronary heart disease (CHD) | 427919004 |
| Mixed myocardial ischemia and infarction (disorder) | Coronary heart disease (CHD) | 428196007 |
| Congenital coronary artery sclerosis (disorder) | Coronary heart disease (CHD) | 42866003 |
| Recent myocardial infarction (situation) | Coronary heart disease (CHD) | 428752002 |
| Recurrent coronary arteriosclerosis after percutaneous transluminal coronary angioplasty (disorder) | Coronary heart disease (CHD) | 429245005 |
| New myocardial infarction compared to prior study (finding) | Coronary heart disease (CHD) | 429391004 |
| Typical angina (disorder) | Coronary heart disease (CHD) | 429559004 |
| Arteriosclerosis of coronary artery bypass graft (disorder) | Coronary heart disease (CHD) | 429673002 |
| Arteriosclerosis of autologous vein coronary artery bypass graft (disorder) | Coronary heart disease (CHD) | 442224005 |
| Arteriosclerosis of nonautologous coronary artery bypass graft (disorder) | Coronary heart disease (CHD) | 442240008 |
| Arteriosclerosis of arterial coronary artery bypass graft (disorder) | Coronary heart disease (CHD) | 442421004 |
| Atherosclerosis of coronary artery (disorder) | Coronary heart disease (CHD) | 443502000 |
| Arteriosclerosis of coronary artery bypass graft of transplanted heart (disorder) | Coronary heart disease (CHD) | 444855007 |
| Arteriosclerosis of internal mammary artery coronary artery bypass graft (disorder) | Coronary heart disease (CHD) | 444856008 |
| Preinfarction syndrome (disorder) | Coronary heart disease (CHD) | 4557003 |
| History of myocardial infarction in last eight weeks (situation) | Coronary heart disease (CHD) | 461000119108 |
| Subendocardial ischemia (disorder) | Coronary heart disease (CHD) | 46109009 |
| Ischemic dilated cardiomyopathy due to coronary artery disease (disorder) | Coronary heart disease (CHD) | 472100003 |
| Acute anteroapical myocardial infarction (disorder) | Coronary heart disease (CHD) | 52035003 |
| Coronary arteriosclerosis (disorder) | Coronary heart disease (CHD) | 53741008 |
| Acute myocardial infarction of anterior wall (disorder) | Coronary heart disease (CHD) | 54329005 |
| Acute myocardial infarction (disorder) | Coronary heart disease (CHD) | 57054005 |
| Acute myocardial infarction of lateral wall (disorder) | Coronary heart disease (CHD) | 58612006 |
| Angina decubitus (disorder) | Coronary heart disease (CHD) | 59021001 |
| Acute myocardial infarction of apical-lateral wall (disorder) | Coronary heart disease (CHD) | 59063002 |
| Aortocoronary artery bypass graft repeated (situation) | Coronary heart disease (CHD) | 61236006 |
| Angina class I (disorder) | Coronary heart disease (CHD) | 61490001 |
| Acute anteroseptal myocardial infarction (disorder) | Coronary heart disease (CHD) | 62695002 |
| Coronary occlusion (disorder) | Coronary heart disease (CHD) | 63739005 |
| Acute myocardial infarction of high lateral wall (disorder) | Coronary heart disease (CHD) | 64627002 |
| Acute myocardial infarction of inferolateral wall (disorder) | Coronary heart disease (CHD) | 65547006 |
| Postmyocardial infarction syndrome (disorder) | Coronary heart disease (CHD) | 66189004 |
| Coronary artery atheroma (disorder) | Coronary heart disease (CHD) | 67682002 |
| Coronary microvascular dysfunction | Coronary heart disease (CHD) | 697976003 |
| Coronary angioplasty planned (situation) | Coronary heart disease (CHD) | 698377004 |
| Coronary artery bypass graft operation planned (situation) | Coronary heart disease (CHD) | 698378009 |
| History of non-ST segment elevation myocardial infarction (situation) | Coronary heart disease (CHD) | 698593009 |
| Acute myocardial infarction of anterolateral wall (disorder) | Coronary heart disease (CHD) | 70211005 |
| Acute ST segment elevation myocardial infarction of anterior wall (disorder) | Coronary heart disease (CHD) | 703164000 |
| Acute ST segment elevation myocardial infarction of anterior wall involving right ventricle (disorder) | Coronary heart disease (CHD) | 703165004 |
| Subsequent ST segment elevation myocardial infarction of inferior wall (disorder) | Coronary heart disease (CHD) | 703209002 |
| Subsequent ST segment elevation myocardial infarction of anterior wall (disorder) | Coronary heart disease (CHD) | 703210007 |
| Subsequent ST segment elevation myocardial infarction (disorder) | Coronary heart disease (CHD) | 703211006 |
| Acute myocardial infarction during procedure (disorder) | Coronary heart disease (CHD) | 703212004 |
| Acute ST segment elevation myocardial infarction of inferior wall (disorder) | Coronary heart disease (CHD) | 703213009 |
| Silent coronary vasospastic disease (disorder) | Coronary heart disease (CHD) | 703214003 |
| Acute myocardial infarction of inferior wall involving right ventricle (disorder) | Coronary heart disease (CHD) | 703251009 |
| Acute myocardial infarction of anterior wall involving right ventricle (disorder) | Coronary heart disease (CHD) | 703252002 |
| Acute ST segment elevation myocardial infarction of inferior wall involving right ventricle (disorder) | Coronary heart disease (CHD) | 703253007 |
| Mitral valve regurgitation due to and following acute myocardial infarction (disorder) | Coronary heart disease (CHD) | 703326006 |
| Mitral valve regurgitation due to acute myocardial infarction without papillary muscle and chordal rupture (disorder) | Coronary heart disease (CHD) | 703328007 |
| Mitral valve regurgitation due to acute myocardial infarction with papillary muscle and chordal rupture (disorder) | Coronary heart disease (CHD) | 703330009 |
| Subsequent non-ST segment elevation myocardial infarction (disorder) | Coronary heart disease (CHD) | 703360004 |
| Acute subendocardial infarction (disorder) | Coronary heart disease (CHD) | 70422006 |
| Acute myocardial infarction of basal inferior segment of left ventricle (disorder) | Coronary heart disease (CHD) | 70998009 |
| Pericarditis due to acute myocardial infarction (disorder) | Coronary heart disease (CHD) | 71023004 |
| Resting ischemia co-occurrent and due to ischemic heart disease (disorder) | Coronary heart disease (CHD) | 712866001 |
| Subacute ischemic heart disease (disorder) | Coronary heart disease (CHD) | 713405002 |
| Non-obstructive atherosclerosis of coronary artery (disorder) | Coronary heart disease (CHD) | 719678003 |
| Ventricular aneurysm due to and following acute myocardial infarction (disorder) | Coronary heart disease (CHD) | 723858002 |
| Pulmonary embolism due to and following acute myocardial infarction (disorder) | Coronary heart disease (CHD) | 723859005 |
| Arrhythmia due to and following acute myocardial infarction (disorder) | Coronary heart disease (CHD) | 723860000 |
| Cardiogenic shock unrelated to mechanical complications as current complication following acute myocardial infarction (disorder) | Coronary heart disease (CHD) | 723861001 |
| Atherosclerosis of non-autologous coronary artery bypass graft (disorder) | Coronary heart disease (CHD) | 723862008 |
| Atherosclerosis of autologous coronary artery bypass graft (disorder) | Coronary heart disease (CHD) | 724431008 |
| Myocardial infarction due to atherothrombotic coronary artery disease (disorder) | Coronary heart disease (CHD) | 726499301000119000 |
| Mural thrombus of right ventricle following acute myocardial infarction (disorder) | Coronary heart disease (CHD) | 736978009 |
| Acute myocardial infarction of inferior wall (disorder) | Coronary heart disease (CHD) | 73795002 |
| Acute myocardial infarction of inferoposterior wall (disorder) | Coronary heart disease (CHD) | 76593002 |
| History of myocardial infarction due to demand ischemia | Coronary heart disease (CHD) | 776219771000119000 |
| Acute coronary artery occlusion not resulting in myocardial infarction (disorder) | Coronary heart disease (CHD) | 78741000119103 |
| Acute myocardial infarction of septum (disorder) | Coronary heart disease (CHD) | 79009004 |
| Angina due to type 2 diabetes mellitus (disorder) | Coronary heart disease (CHD) | 791000119109 |
| Coronary arteriosclerosis in artery of transplanted heart (disorder) | Coronary heart disease (CHD) | 792842004 |
| Coronary microvascular disease (disorder) | Coronary heart disease (CHD) | 810681000000101 |
| Ischemic contracture of left ventricle syndrome (disorder) | Coronary heart disease (CHD) | 82522008 |
| Early postmyocardial infarction pericarditis (disorder) | Coronary heart disease (CHD) | 827163002 |
| Delayed postmyocardial infarction pericarditis (disorder) | Coronary heart disease (CHD) | 827164008 |
| Acute myocardial infarction of right ventricle (disorder) | Coronary heart disease (CHD) | 836293000 |
| Acute myocardial infarction of apex of heart (disorder) | Coronary heart disease (CHD) | 836294006 |
| Acute myocardial infarction of inferolateral wall with posterior extension (disorder) | Coronary heart disease (CHD) | 836295007 |
| Acute ST segment elevation myocardial infarction due to occlusion of proximal portion of anterior descending branch of left coronary artery (disorder) | Coronary heart disease (CHD) | 840309000 |
| Occlusion of proximal portion of anterior descending branch of left coronary artery (disorder) | Coronary heart disease (CHD) | 840310005 |
| Acute ST segment elevation myocardial infarction due to occlusion of mid portion of anterior descending branch of left coronary artery (disorder) | Coronary heart disease (CHD) | 840312002 |
| Occlusion of mid portion of anterior descending branch of left coronary artery (disorder) | Coronary heart disease (CHD) | 840313007 |
| Occlusion of distal portion of anterior descending branch of left coronary artery (disorder) | Coronary heart disease (CHD) | 840315000 |
| Acute ST segment elevation myocardial infarction due to occlusion of distal portion of anterior descending branch of left coronary artery (disorder) | Coronary heart disease (CHD) | 840316004 |
| Occlusion of anterior descending branch of left coronary artery (disorder) | Coronary heart disease (CHD) | 840608004 |
| Acute ST segment elevation myocardial infarction due to occlusion of anterior descending branch of left coronary artery (disorder) | Coronary heart disease (CHD) | 840609007 |
| Occlusion of septal branch of anterior descending branch of left coronary artery (disorder) | Coronary heart disease (CHD) | 840679006 |
| Acute ST segment elevation myocardial infarction due to occlusion of septal branch of anterior descending branch of left coronary artery (disorder) | Coronary heart disease (CHD) | 840680009 |
| Occlusion of diagonal branch of anterior descending branch of left coronary artery (disorder) | Coronary heart disease (CHD) | 846667001 |
| Acute ST segment elevation myocardial infarction due to occlusion of diagonal branch of anterior descending branch of left coronary artery (disorder) | Coronary heart disease (CHD) | 846668006 |
| Acute ST segment elevation myocardial infarction due to occlusion of intermediate artery (disorder) | Coronary heart disease (CHD) | 846683001 |
| Occlusion of intermediate artery (disorder) | Coronary heart disease (CHD) | 846684007 |
| Angina class III (disorder) | Coronary heart disease (CHD) | 85284003 |
| Acute ST segment elevation myocardial infarction due to occlusion of proximal portion of right coronary artery (disorder) | Coronary heart disease (CHD) | 868214006 |
| Occlusion of proximal portion of right coronary artery (disorder) | Coronary heart disease (CHD) | 868215007 |
| Occlusion of distal portion of right coronary artery (disorder) | Coronary heart disease (CHD) | 868216008 |
| Acute ST segment elevation myocardial infarction due to occlusion of distal portion of right coronary artery (disorder) | Coronary heart disease (CHD) | 868217004 |
| Occlusion of mid portion of right coronary artery (disorder) | Coronary heart disease (CHD) | 868219001 |
| Acute ST segment elevation myocardial infarction due to occlusion of mid portion of right coronary artery (disorder) | Coronary heart disease (CHD) | 868220007 |
| Occlusion of marginal branch of right coronary artery (disorder) | Coronary heart disease (CHD) | 868221006 |
| Occlusion of posterior descending branch of right coronary artery (disorder) | Coronary heart disease (CHD) | 868222004 |
| Occlusion of posterior lateral branch of right coronary artery (disorder) | Coronary heart disease (CHD) | 868223009 |
| Acute ST segment elevation myocardial infarction due to occlusion of marginal branch of right coronary artery (disorder) | Coronary heart disease (CHD) | 868224003 |
| Acute ST segment elevation myocardial infarction due to occlusion of posterior descending branch of right coronary artery (disorder) | Coronary heart disease (CHD) | 868225002 |
| Acute ST segment elevation myocardial infarction due to occlusion of posterior lateral branch of right coronary artery (disorder) | Coronary heart disease (CHD) | 868226001 |
| Stenosis of right coronary artery (disorder) | Coronary heart disease (CHD) | 876856005 |
| Stenosis of left coronary artery main stem (disorder) | Coronary heart disease (CHD) | 876857001 |
| Stenosis of circumflex branch of left coronary artery (disorder) | Coronary heart disease (CHD) | 876858006 |
| Stenosis of anterior descending branch of left coronary artery (disorder) | Coronary heart disease (CHD) | 876859003 |
| Myocardial infarction with non-obstructive coronary artery (disorder) | Coronary heart disease (CHD) | 879955009 |
| Angina class IV (disorder) | Coronary heart disease (CHD) | 89323001 |
| Acute myocardial infarction due to occlusion of circumflex branch of left coronary artery (disorder) | Coronary heart disease (CHD) | 896689003 |
| Occlusion of circumflex branch of left coronary artery (disorder) | Coronary heart disease (CHD) | 896690007 |
| Acute ST segment elevation myocardial infarction due to occlusion of circumflex branch of left coronary artery (disorder) | Coronary heart disease (CHD) | 896691006 |
| Acute ST segment elevation myocardial infarction of apex of heart (disorder) | Coronary heart disease (CHD) | 896696001 |
| Acute ST segment elevation myocardial infarction of right ventricle (disorder) | Coronary heart disease (CHD) | 896697005 |
| Calcific coronary arteriosclerosis (disorder) | Coronary heart disease (CHD) | 92517006 |
| Cerebrovascular accident due to embolism of bilateral anterior cerebral arteries (disorder) | Stroke diagnosis | 107557061000119000 |
| Haemorrhagic stroke (disorder) | Stroke diagnosis | 1078001000000100 |
| Cerebral infarction due to occlusion of cerebral artery (disorder) | Stroke diagnosis | 1089411000000100 |
| Cerebral infarction due to stenosis of cerebral artery (disorder) | Stroke diagnosis | 1089421000000100 |
| Nonparalytic stroke (disorder) | Stroke diagnosis | 111297002 |
| Occlusion of anterior cerebral artery (disorder) | Stroke diagnosis | 1153543002 |
| Occlusion of right anterior cerebral artery (disorder) | Stroke diagnosis | 1153544008 |
| Occlusion of left anterior cerebral artery (disorder) | Stroke diagnosis | 1153545009 |
| Occlusion of bilateral posterior cerebral arteries (disorder) | Stroke diagnosis | 1153546005 |
| Occlusion of right posterior communicating artery (disorder) | Stroke diagnosis | 1153607003 |
| Occlusion of left posterior communicating artery (disorder) | Stroke diagnosis | 1153608008 |
| Embolism of left anterior cerebral artery (disorder) | Stroke diagnosis | 1153611009 |
| Embolism of right anterior cerebral artery (disorder) | Stroke diagnosis | 1153612002 |
| Embolism of left carotid artery (disorder) | Stroke diagnosis | 1153630009 |
| Embolism of right carotid artery (disorder) | Stroke diagnosis | 1153631008 |
| Embolism of bilateral middle cerebral arteries (disorder) | Stroke diagnosis | 1153632001 |
| Embolism of bilateral posterior cerebral arteries (disorder) | Stroke diagnosis | 1153633006 |
| Embolism of bilateral anterior cerebral arteries (disorder) | Stroke diagnosis | 1153634000 |
| Occlusion of bilateral cerebellar arteries (disorder) | Stroke diagnosis | 1153638002 |
| Embolism of bilateral carotid arteries (disorder) | Stroke diagnosis | 1155688007 |
| Embolism of bilateral cerebellar arteries (disorder) | Stroke diagnosis | 1155689004 |
| Embolism of left vertebral artery (disorder) | Stroke diagnosis | 1155697006 |
| Embolism of right vertebral artery (disorder) | Stroke diagnosis | 1155698001 |
| Embolism of bilateral vertebral arteries (disorder) | Stroke diagnosis | 1155699009 |
| Thrombosis of left posterior cerebral artery (disorder) | Stroke diagnosis | 1156016006 |
| Thrombosis of right posterior cerebral artery (disorder) | Stroke diagnosis | 1156017002 |
| Thrombosis of left cerebellar artery (disorder) | Stroke diagnosis | 1156018007 |
| Thrombosis of right cerebellar artery (disorder) | Stroke diagnosis | 1156019004 |
| Thrombus of dural sinus in pregnancy (disorder) | Stroke diagnosis | 1156027008 |
| Thrombus of dural sinus in puerperium (disorder) | Stroke diagnosis | 1156029006 |
| Paralytic stroke (disorder) | Stroke diagnosis | 116288000 |
| Hemorrhagic cerebral infarction caused by Aspergillus | Stroke diagnosis | 1163482004 |
| Cerebrovascular accident due to occlusion of left posterior communicating artery (disorder) | Stroke diagnosis | 117776611000119000 |
| Fetal epilepsy due to perinatal stroke (disorder) | Stroke diagnosis | 1179360000 |
| Childhood arterial ischemic stroke | Stroke diagnosis | 1197363004 |
| Asymptomatic occlusion of intracranial vertebral artery (disorder) | Stroke diagnosis | 1204189004 |
| Occlusion of cerebral artery due to infection (disorder) | Stroke diagnosis | 1204202004 |
| Malignant middle cerebral artery syndrome (disorder) | Stroke diagnosis | 1231168008 |
| Cerebral infarction due to occlusion of precerebral artery (disorder) | Stroke diagnosis | 125081000119106 |
| Acute cerebrovascular accident due to occlusion of right posterior cerebral artery (disorder) | Stroke diagnosis | 137592291000119000 |
| Ischemic stroke without coma (disorder) | Stroke diagnosis | 140921000119102 |
| Anterior choroidal artery syndrome (disorder) | Stroke diagnosis | 14309005 |
| Cerebrovascular accident due to embolism of bilateral carotid arteries (disorder) | Stroke diagnosis | 152148641000119000 |
| Thrombosis of right vertebral artery (disorder) | Stroke diagnosis | 15978431000119100 |
| Cerebrovascular accident due to occlusion of left posterior cerebral artery (disorder) | Stroke diagnosis | 16000351000119100 |
| Cerebrovascular accident due to occlusion of right posterior cerebral artery (disorder) | Stroke diagnosis | 16000391000119100 |
| Cerebrovascular accident due to occlusion of right middle cerebral artery (disorder) | Stroke diagnosis | 16000431000119100 |
| Cerebrovascular accident due to occlusion of left middle cerebral artery (disorder) | Stroke diagnosis | 16000511000119100 |
| Cerebrovascular accident due to thrombus of right middle cerebral artery (disorder) | Stroke diagnosis | 16002031000119100 |
| Cerebrovascular accident due to thrombus of left middle cerebral artery (disorder) | Stroke diagnosis | 16002111000119100 |
| Cerebrovascular accident due to occlusion of right carotid artery (disorder) | Stroke diagnosis | 16023911000119100 |
| Cerebrovascular accident due to occlusion of left carotid artery (disorder) | Stroke diagnosis | 16024111000119100 |
| Cerebrovascular accident due to occlusion of left cerebellar artery (disorder) | Stroke diagnosis | 16024151000119100 |
| Cerebrovascular accident due to occlusion of right cerebellar artery (disorder) | Stroke diagnosis | 16024271000119100 |
| Cerebrovascular accident due to stenosis of right carotid artery (disorder) | Stroke diagnosis | 16026951000119100 |
| Cerebrovascular accident due to stenosis of left carotid artery (disorder) | Stroke diagnosis | 16026991000119100 |
| Acute cerebral ischemia (disorder) | Stroke diagnosis | 16218291000119100 |
| Cerebellar stroke (disorder) | Stroke diagnosis | 16371781000119100 |
| Cerebrovascular accident due to occlusion of bilateral pontine arteries (disorder) | Stroke diagnosis | 16644681000119100 |
| Cerebrovascular accident due to stenosis of bilateral vertebral arteries (disorder) | Stroke diagnosis | 16661931000119100 |
| Cerebrovascular accident due to stenosis of bilateral carotid arteries (disorder) | Stroke diagnosis | 16661971000119100 |
| Cerebrovascular accident due to embolism of bilateral posterior cerebral arteries (disorder) | Stroke diagnosis | 168747591000119000 |
| Idiopathic ischemic stroke | Stroke diagnosis | 16891111000119100 |
| History of embolic cerebrovascular accident (situation) | Stroke diagnosis | 16896851000119100 |
| History of cerebrovascular accident due to ischemia | Stroke diagnosis | 16896891000119100 |
| Cerebrovascular accident due to occlusion of left anterior cerebral artery (disorder) | Stroke diagnosis | 182960891000119000 |
| Cerebrovascular accident due to occlusion of bilateral middle cerebral arteries (disorder) | Stroke diagnosis | 188174841000119000 |
| Basal ganglia hemorrhage (disorder) | Stroke diagnosis | 195165005 |
| External capsule hemorrhage (disorder) | Stroke diagnosis | 195167002 |
| Intracerebral hemorrhage with intraventricular hemorrhage (disorder) | Stroke diagnosis | 195168007 |
| Intracerebral hemorrhage multiple localized (disorder) | Stroke diagnosis | 195169004 |
| Cerebral infarct due to thrombosis of precerebral arteries (disorder) | Stroke diagnosis | 195185009 |
| Cerebral infarction due to embolism of precerebral arteries (disorder) | Stroke diagnosis | 195186005 |
| Cerebral infarction due to thrombosis of cerebral arteries (disorder) | Stroke diagnosis | 195189003 |
| Cerebral infarction due to embolism of cerebral arteries (disorder) | Stroke diagnosis | 195190007 |
| Middle cerebral artery syndrome (disorder) | Stroke diagnosis | 195209007 |
| Anterior cerebral artery syndrome (disorder) | Stroke diagnosis | 195210002 |
| Posterior cerebral artery syndrome (disorder) | Stroke diagnosis | 195211003 |
| Brainstem stroke syndrome (disorder) | Stroke diagnosis | 195212005 |
| Cerebellar stroke syndrome (disorder) | Stroke diagnosis | 195213000 |
| Left sided cerebral hemisphere cerebrovascular accident (disorder) | Stroke diagnosis | 195216008 |
| Right sided cerebral hemisphere cerebrovascular accident (disorder) | Stroke diagnosis | 195217004 |
| Cerebral infarction due to cerebral venous thrombosis non-pyogenic (disorder) | Stroke diagnosis | 195230003 |
| Occlusion of cerebral artery (disorder) | Stroke diagnosis | 20059004 |
| Subcortical cerebral hemorrhage (disorder) | Stroke diagnosis | 20908003 |
| Infarction of optic tract (disorder) | Stroke diagnosis | 230518009 |
| Infarction of optic radiation (disorder) | Stroke diagnosis | 230523009 |
| Cerebrovascular accident (disorder) | Stroke diagnosis | 230690007 |
| Cerebrovascular accident due to occlusion of cerebral artery (disorder) | Stroke diagnosis | 230691006 |
| Infarction - precerebral (disorder) | Stroke diagnosis | 230692004 |
| Anterior cerebral circulation infarction (disorder) | Stroke diagnosis | 230693009 |
| Total anterior cerebral circulation infarction (disorder) | Stroke diagnosis | 230694003 |
| Partial anterior cerebral circulation infarction (disorder) | Stroke diagnosis | 230695002 |
| Posterior cerebral circulation infarction (disorder) | Stroke diagnosis | 230696001 |
| Lacunar infarction (disorder) | Stroke diagnosis | 230698000 |
| Pure motor lacunar infarction (disorder) | Stroke diagnosis | 230699008 |
| Pure sensory lacunar infarction (disorder) | Stroke diagnosis | 230700009 |
| Pure sensorimotor lacunar infarction (disorder) | Stroke diagnosis | 230701008 |
| Lacunar ataxic hemiparesis (disorder) | Stroke diagnosis | 230702001 |
| Dysarthria-clumsy hand syndrome (disorder) | Stroke diagnosis | 230703006 |
| Multi-infarct state (disorder) | Stroke diagnosis | 230704000 |
| Hemorrhagic cerebral infarction (disorder) | Stroke diagnosis | 230706003 |
| Anterior cerebral circulation hemorrhagic infarction (disorder) | Stroke diagnosis | 230707007 |
| Posterior cerebral circulation hemorrhagic infarction (disorder) | Stroke diagnosis | 230708002 |
| Massive supratentorial cerebral hemorrhage (disorder) | Stroke diagnosis | 230709005 |
| Lobar cerebral hemorrhage (disorder) | Stroke diagnosis | 230710000 |
| Thalamic hemorrhage (disorder) | Stroke diagnosis | 230711001 |
| Lacunar hemorrhage (disorder) | Stroke diagnosis | 230712008 |
| Stroke of uncertain pathology (disorder) | Stroke diagnosis | 230713003 |
| Anterior circulation stroke of uncertain pathology (disorder) | Stroke diagnosis | 230714009 |
| Posterior circulation stroke of uncertain pathology (disorder) | Stroke diagnosis | 230715005 |
| Cerebrovascular accident due to occlusion of basilar artery (disorder) | Stroke diagnosis | 239965291000119000 |
| Weber-Gubler syndrome (disorder) | Stroke diagnosis | 24654003 |
| Completed stroke (disorder) | Stroke diagnosis | 25133001 |
| Cerebrovascular accident due to embolism of left anterior cerebral artery (disorder) | Stroke diagnosis | 251770561000119000 |
| Acute cerebrovascular accident due to occlusion of right middle cerebral artery (disorder) | Stroke diagnosis | 272202251000119000 |
| Cerebral hemorrhage (disorder) | Stroke diagnosis | 274100004 |
| Stroke in the puerperium (disorder) | Stroke diagnosis | 275434003 |
| Occipital cerebral infarction (disorder) | Stroke diagnosis | 276219001 |
| Foville syndrome (disorder) | Stroke diagnosis | 276220007 |
| Millard-Gubler syndrome (disorder) | Stroke diagnosis | 276221006 |
| Top of basilar syndrome (disorder) | Stroke diagnosis | 276222004 |
| Intracerebellar and posterior fossa hemorrhage (disorder) | Stroke diagnosis | 276722003 |
| Extension of cerebrovascular accident (disorder) | Stroke diagnosis | 281240008 |
| Dysphagia due to and following non-traumatic intracerebral hemorrhage (disorder) | Stroke diagnosis | 290641000119107 |
| Spontaneous hemorrhage of deep cerebral hemisphere (disorder) | Stroke diagnosis | 291511000119103 |
| Spontaneous hemorrhage of cortical intracerebral hemisphere (disorder) | Stroke diagnosis | 291521000119105 |
| Spontaneous hemorrhage of cerebral hemisphere (disorder) | Stroke diagnosis | 291531000119108 |
| Spontaneous hemorrhage of brain stem (disorder) | Stroke diagnosis | 291541000119104 |
| Lacunar ataxic hemiparesis of right dominant side (disorder) | Stroke diagnosis | 292851000119109 |
| Lacunar ataxic hemiparesis of left dominant side (disorder) | Stroke diagnosis | 292861000119106 |
| Embolus of circle of Willis (disorder) | Stroke diagnosis | 297138001 |
| Infarction of optic chiasm (disorder) | Stroke diagnosis | 302902003 |
| Infarction of visual cortex (disorder) | Stroke diagnosis | 302904002 |
| Multiple lacunar infarcts (disorder) | Stroke diagnosis | 307363008 |
| Left sided cerebral infarction (disorder) | Stroke diagnosis | 307766002 |
| Right sided cerebral infarction (disorder) | Stroke diagnosis | 307767006 |
| Right sided intracerebral hemorrhage unspecified (disorder) | Stroke diagnosis | 308128006 |
| Cerebrovascular accident due to occlusion of right middle cerebral artery by embolus (disorder) | Stroke diagnosis | 329361000119107 |
| Cerebrovascular accident due to occlusion of left middle cerebral artery by embolus (disorder) | Stroke diagnosis | 329371000119101 |
| Cerebrovascular accident due to occlusion of right posterior cerebral artery by embolus (disorder) | Stroke diagnosis | 329421000119107 |
| Cerebrovascular accident due to occlusion of left posterior cerebral artery by embolus (disorder) | Stroke diagnosis | 329431000119105 |
| Cerebrovascular accident due to occlusion of right cerebellar artery by embolus (disorder) | Stroke diagnosis | 329451000119104 |
| Cerebrovascular accident due to occlusion of left cerebellar artery by embolus (disorder) | Stroke diagnosis | 329461000119102 |
| Occlusion of right middle cerebral artery (disorder) | Stroke diagnosis | 329481000119106 |
| Occlusion of left middle cerebral artery (disorder) | Stroke diagnosis | 329491000119109 |
| Occlusion of bilateral middle cerebral arteries (disorder) | Stroke diagnosis | 329501000119102 |
| Occlusion of bilateral anterior cerebral arteries (disorder) | Stroke diagnosis | 329541000119100 |
| Occlusion of right posterior cerebral artery (disorder) | Stroke diagnosis | 329561000119101 |
| Occlusion of left posterior cerebral artery (disorder) | Stroke diagnosis | 329571000119107 |
| Cerebrovascular accident due to thrombus of basilar artery (disorder) | Stroke diagnosis | 329641000119104 |
| Cerebrovascular accident due to thrombus of right carotid artery (disorder) | Stroke diagnosis | 329651000119102 |
| Lacunar ataxic hemiparesis of left nondominant side (disorder) | Stroke diagnosis | 330411000119109 |
| Lacunar ataxic hemiparesis of right nondominant side (disorder) | Stroke diagnosis | 330421000119102 |
| Cerebrovascular accident due to thrombus of left carotid artery (disorder) | Stroke diagnosis | 330791000119108 |
| Acute cerebrovascular accident due to thrombosis of left middle cerebral artery (disorder) | Stroke diagnosis | 346410121000119000 |
| Cerebrovascular accident due to occlusion of bilateral cerebellar arteries (disorder) | Stroke diagnosis | 346674811000119000 |
| Thrombotic stroke (disorder) | Stroke diagnosis | 371040005 |
| Embolic stroke (disorder) | Stroke diagnosis | 371041009 |
| Occlusive stroke (disorder) | Stroke diagnosis | 373606000 |
| Cerebrovascular accident due to embolism of right carotid artery (disorder) | Stroke diagnosis | 384430101000119000 |
| Cerebrovascular accident due to thrombosis of right posterior cerebral artery (disorder) | Stroke diagnosis | 38595071000119100 |
| Acute cerebrovascular accident due to occlusion of left posterior cerebral artery (disorder) | Stroke diagnosis | 40076141000119100 |
| Acute cerebrovascular accident due to occlusion of right carotid artery (disorder) | Stroke diagnosis | 407573811000119000 |
| Infarction of basal ganglia (disorder) | Stroke diagnosis | 413102000 |
| Cardioembolic stroke (disorder) | Stroke diagnosis | 413758000 |
| Ischemic stroke (disorder) | Stroke diagnosis | 422504002 |
| Acute lacunar infarction (disorder) | Stroke diagnosis | 426107000 |
| Infarction of medulla oblongata (disorder) | Stroke diagnosis | 426983002 |
| Cerebral infarction (disorder) | Stroke diagnosis | 432504007 |
| Superior cerebellar artery syndrome (disorder) | Stroke diagnosis | 444657001 |
| Acute cerebrovascular accident due to stenosis of left carotid artery (disorder) | Stroke diagnosis | 46583221000119100 |
| Cortical hemorrhage (disorder) | Stroke diagnosis | 49422009 |
| Cerebrovascular accident due to thrombosis of right cerebellar artery (disorder) | Stroke diagnosis | 496369931000119000 |
| Acute cerebrovascular accident due to occlusion of left carotid artery (disorder) | Stroke diagnosis | 502836591000119000 |
| Cerebrovascular accident due to thrombosis of right vertebral artery (disorder) | Stroke diagnosis | 511452481000119000 |
| Cerebrovascular accident due to embolism of left vertebral artery (disorder) | Stroke diagnosis | 517253051000119000 |
| Internal capsule hemorrhage (disorder) | Stroke diagnosis | 52201006 |
| Cerebrovascular accident with intracranial hemorrhage | Stroke diagnosis | 5571000124103 |
| Multi-infarct dementia (disorder) | Stroke diagnosis | 56267009 |
| Progressing stroke (disorder) | Stroke diagnosis | 57981008 |
| Cerebrovascular accident due to embolism of bilateral cerebellar arteries (disorder) | Stroke diagnosis | 58173271000119100 |
| Cerebrovascular accident of basal ganglia (disorder) | Stroke diagnosis | 595899961000119000 |
| Acute cerebrovascular accident due to occlusion of left middle cerebral artery (disorder) | Stroke diagnosis | 63230211000119100 |
| Cerebrovascular accident of brainstem (disorder) | Stroke diagnosis | 652287331000119000 |
| Cerebrovascular accident due to occlusion of right anterior cerebral artery (disorder) | Stroke diagnosis | 655081461000119000 |
| Acute cerebrovascular accident due to stenosis of right carotid artery (disorder) | Stroke diagnosis | 693158221000119000 |
| Cerebral arterial thrombosis (disorder) | Stroke diagnosis | 71444005 |
| Cerebrovascular accident due to occlusion of bilateral anterior cerebral arteries (disorder) | Stroke diagnosis | 720261501000119000 |
| Silent cerebral infarct (disorder) | Stroke diagnosis | 723082006 |
| Cerebral ischemic stroke due to small artery occlusion (disorder) | Stroke diagnosis | 724424009 |
| Stroke co-occurrent with migraine (disorder) | Stroke diagnosis | 724429004 |
| Cerebrovascular accident due to embolism of bilateral vertebral arteries (disorder) | Stroke diagnosis | 732330391000119000 |
| Hemorrhage of medulla oblongata (disorder) | Stroke diagnosis | 732923001 |
| Thrombosis of left middle cerebral artery (disorder) | Stroke diagnosis | 734383005 |
| Thrombosis of right middle cerebral artery (disorder) | Stroke diagnosis | 734384004 |
| Embolus of left posterior cerebral artery (disorder) | Stroke diagnosis | 734961002 |
| Embolus of right posterior cerebral artery (disorder) | Stroke diagnosis | 734963004 |
| Embolus of left middle cerebral artery (disorder) | Stroke diagnosis | 734964005 |
| Embolus of right middle cerebral artery (disorder) | Stroke diagnosis | 734965006 |
| Acute cerebrovascular accident due to thrombosis of right middle cerebral artery (disorder) | Stroke diagnosis | 738211801000119000 |
| Acute cerebrovascular accident due to occlusion of right cerebellar artery (disorder) | Stroke diagnosis | 738478141000119000 |
| Cerebellar hemorrhage (disorder) | Stroke diagnosis | 75038005 |
| Cerebral embolism (disorder) | Stroke diagnosis | 75543006 |
| Cerebrovascular accident due to thrombosis of left vertebral artery (disorder) | Stroke diagnosis | 759950981000119000 |
| Acute ischemic stroke | Stroke diagnosis | 762005171000119000 |
| Occlusion of right middle cerebral artery by embolus (disorder) | Stroke diagnosis | 762629007 |
| Occlusion of left middle cerebral artery by embolus (disorder) | Stroke diagnosis | 762630002 |
| Occlusion of right posterior cerebral artery by embolus (disorder) | Stroke diagnosis | 762651004 |
| Occlusion of left posterior cerebral artery by embolus (disorder) | Stroke diagnosis | 762652006 |
| Cerebrovascular accident of thalamus (disorder) | Stroke diagnosis | 769023031000119000 |
| Intrapontine hemorrhage (disorder) | Stroke diagnosis | 7713009 |
| Posterior inferior cerebellar artery syndrome (disorder) | Stroke diagnosis | 78569004 |
| Acute cerebrovascular accident due to occlusion of left cerebellar artery (disorder) | Stroke diagnosis | 788310011000119000 |
| Occlusion of bilateral pontine arteries (disorder) | Stroke diagnosis | 788455001 |
| Cerebral ischemic stroke due to dissection of artery | Stroke diagnosis | 788880006 |
| Cerebral ischemic stroke due to aortic arch embolism | Stroke diagnosis | 788881005 |
| Cerebral ischemic stroke due to global hypoperfusion with watershed infarct | Stroke diagnosis | 788882003 |
| Cerebral ischemic stroke due to hypercoagulable state | Stroke diagnosis | 788883008 |
| Cerebral ischemic stroke due to subarachnoid hemorrhage | Stroke diagnosis | 788884002 |
| Cerebrovascular accident due to embolism of right vertebral artery (disorder) | Stroke diagnosis | 806161651000119000 |
| Cerebrovascular accident due to embolism of right anterior cerebral artery (disorder) | Stroke diagnosis | 849488701000119000 |
| Cerebrovascular accident due to occlusion of right posterior communicating artery (disorder) | Stroke diagnosis | 849579281000119000 |
| Cerebrovascular accident due to thrombosis of left posterior cerebral artery (disorder) | Stroke diagnosis | 851365731000119000 |
| Cerebrovascular accident due to embolism of left carotid artery (disorder) | Stroke diagnosis | 859422751000119000 |
| Cerebrovascular accident due to occlusion of bilateral posterior cerebral arteries (disorder) | Stroke diagnosis | 86553761000119100 |
| Occlusion of distal basilar artery (disorder) | Stroke diagnosis | 870544005 |
| Occlusion of branch of basilar artery (disorder) | Stroke diagnosis | 870579007 |
| Claude's syndrome (disorder) | Stroke diagnosis | 87555007 |
| Cerebrovascular accident of medulla oblongata (disorder) | Stroke diagnosis | 881694631000119000 |
| Cerebrovascular accident due to occlusion of bilateral vertebral arteries (disorder) | Stroke diagnosis | 898941951000119000 |
| Cerebrovascular accident due to embolism of basilar artery (disorder) | Stroke diagnosis | 915141931000119000 |
| Acute cerebrovascular accident due to embolism of right middle cerebral artery (disorder) | Stroke diagnosis | 931164671000119000 |
| Acute cerebrovascular accident due to embolism of left middle cerebral artery (disorder) | Stroke diagnosis | 936648941000119000 |
| Cerebrovascular accident due to embolism of bilateral middle cerebral arteries (disorder) | Stroke diagnosis | 939885431000119000 |
| Brain stem hemorrhage (disorder) | Stroke diagnosis | 95454007 |
| Brain stem infarction (disorder) | Stroke diagnosis | 95457000 |
| Cerebellar infarction (disorder) | Stroke diagnosis | 95460007 |
| Cerebrovascular accident due to thrombosis of left cerebellar artery (disorder) | Stroke diagnosis | 957319791000119000 |
| Cerebral infarction due to stenosis of carotid artery (disorder) | Stroke diagnosis | 99451000119105 |
| Transient ischemic attack co-occurrent with subarachnoid hemorrhage | Transient ischaemic attack (TIA) | 1208871009 |
| Subclavian steal syndrome (disorder) | Transient ischaemic attack (TIA) | 15258001 |
| History of transient ischemic attack | Transient ischaemic attack (TIA) | 161511000 |
| Acute cerebral ischemia (disorder) | Transient ischaemic attack (TIA) | 16218291000119100 |
| Vertebrobasilar artery syndrome (disorder) | Transient ischaemic attack (TIA) | 195199008 |
| Carotid artery syndrome hemispheric (disorder) | Transient ischaemic attack (TIA) | 195200006 |
| Multiple and bilateral precerebral artery syndromes (disorder) | Transient ischaemic attack (TIA) | 195201005 |
| Impending cerebral ischemia (disorder) | Transient ischaemic attack (TIA) | 195205001 |
| Intermittent cerebral ischemia (disorder) | Transient ischaemic attack (TIA) | 195206000 |
| Carotid territory transient ischemic attack (disorder) | Transient ischaemic attack (TIA) | 230716006 |
| Vertebrobasilar territory transient ischemic attack (disorder) | Transient ischaemic attack (TIA) | 230717002 |
| Transient ischemic attack (disorder) | Transient ischaemic attack (TIA) | 266257000 |
| Vertebral artery syndrome (disorder) | Transient ischaemic attack (TIA) | 34781003 |
| Transient cerebral ischemia due to atrial fibrillation (disorder) | Transient ischaemic attack (TIA) | 426814001 |
| Recurrent transient cerebral ischemic attack (disorder) | Transient ischaemic attack (TIA) | 444172003 |
| Basilar artery syndrome (disorder) | Transient ischaemic attack (TIA) | 64009001 |
| Personal history of transient ischaemic attack (situation) | Transient ischaemic attack (TIA) | 751371000000107 |
| Pain at rest of bilateral lower limbs co-occurrent and due to atherosclerosis (disorder) | Peripheral arterial disease (PAD) diagnostic | 12236991000119100 |
| Pain at rest of left lower limb co-occurrent and due to atherosclerosis (disorder) | Peripheral arterial disease (PAD) diagnostic | 12237111000119100 |
| Pain at rest of right lower limb co-occurrent and due to atherosclerosis (disorder) | Peripheral arterial disease (PAD) diagnostic | 12237231000119100 |
| Peripheral angiopathy due to diabetes mellitus (disorder) | Peripheral arterial disease (PAD) diagnostic | 127014009 |
| Gangrene of right lower limb due to atherosclerosis (disorder) | Peripheral arterial disease (PAD) diagnostic | 15649901000119100 |
| Gangrene of bilateral lower limbs co-occurrent and due to atherosclerosis (disorder) | Peripheral arterial disease (PAD) diagnostic | 15649941000119100 |
| Gangrene of left lower limb due to atherosclerosis (disorder) | Peripheral arterial disease (PAD) diagnostic | 15649991000119100 |
| Intermittent claudication of bilateral lower limbs due to atherosclerosis of nonbiological bypass graft (finding) | Peripheral arterial disease (PAD) diagnostic | 16013431000119100 |
| Peripheral ischemia (disorder) | Peripheral arterial disease (PAD) diagnostic | 233958001 |
| Lower limb ischemia (disorder) | Peripheral arterial disease (PAD) diagnostic | 233961000 |
| Critical lower limb ischemia (disorder) | Peripheral arterial disease (PAD) diagnostic | 233962007 |
| Claudication (finding) | Peripheral arterial disease (PAD) diagnostic | 275520000 |
| Ischemia of feet (disorder) | Peripheral arterial disease (PAD) diagnostic | 300917007 |
| Ischemic foot (disorder) | Peripheral arterial disease (PAD) diagnostic | 301755001 |
| Trash foot (disorder) | Peripheral arterial disease (PAD) diagnostic | 307406004 |
| Ischemic toe (disorder) | Peripheral arterial disease (PAD) diagnostic | 307408003 |
| Peripheral angiopathy due to type 1 diabetes mellitus (disorder) | Peripheral arterial disease (PAD) diagnostic | 31211000119101 |
| Critical ischemia of foot (disorder) | Peripheral arterial disease (PAD) diagnostic | 312822006 |
| Peripheral angiopathy due to type 2 diabetes mellitus (disorder) | Peripheral arterial disease (PAD) diagnostic | 314902007 |
| Peripheral vascular disease associated with another disorder (disorder) | Peripheral arterial disease (PAD) diagnostic | 34881000119105 |
| Peripheral arterial occlusive disease (disorder) | Peripheral arterial disease (PAD) diagnostic | 399957001 |
| Peripheral vascular disease (disorder) | Peripheral arterial disease (PAD) diagnostic | 400047006 |
| Intermittent claudication (finding) | Peripheral arterial disease (PAD) diagnostic | 63491006 |
| Ischemic foot with rest pain | Peripheral arterial disease (PAD) diagnostic | 713412006 |
| Acute occlusion of artery of lower limb co-occurrent and due to thromboembolus (disorder) | Peripheral arterial disease (PAD) diagnostic | 723870003 |
| Limb pain at rest due to atherosclerosis of artery of lower limb (disorder) | Peripheral arterial disease (PAD) diagnostic | 792844003 |
| Ulcer of heel due to atherosclerosis of artery of lower limb (disorder) | Peripheral arterial disease (PAD) diagnostic | 792846001 |
| Ulcer of ankle due to atherosclerosis of artery of lower limb (disorder) | Peripheral arterial disease (PAD) diagnostic | 792850008 |
| Ulcer of calf due to atherosclerosis of artery of lower limb (disorder) | Peripheral arterial disease (PAD) diagnostic | 792855003 |
| Ischaemic lower limb pain at rest (finding) | Peripheral arterial disease (PAD) diagnostic | 836711000000108 |
| Peripheral arterial disease (disorder) | Peripheral arterial disease (PAD) diagnostic | 840580004 |
| Paroxysmal atrial fibrillation with rapid ventricular response (disorder) | Atrial fibrillation | 1010405004 |
| Atrial fibrillation with rapid ventricular response (disorder) | Atrial fibrillation | 120041000119109 |
| Atypical atrial flutter (disorder) | Atrial fibrillation | 15964901000119100 |
| Atrial fibrillation and flutter (disorder) | Atrial fibrillation | 195080001 |
| Lone atrial fibrillation (disorder) | Atrial fibrillation | 233910005 |
| Non-rheumatic atrial fibrillation (disorder) | Atrial fibrillation | 233911009 |
| Paroxysmal atrial fibrillation (disorder) | Atrial fibrillation | 282825002 |
| Controlled atrial fibrillation (disorder) | Atrial fibrillation | 300996004 |
| Rapid atrial fibrillation (disorder) | Atrial fibrillation | 314208002 |
| Chronic atrial flutter (disorder) | Atrial fibrillation | 425615007 |
| Chronic atrial fibrillation (disorder) | Atrial fibrillation | 426749004 |
| Paroxysmal atrial flutter (disorder) | Atrial fibrillation | 427665004 |
| Permanent atrial fibrillation (disorder) | Atrial fibrillation | 440028005 |
| Persistent atrial fibrillation (disorder) | Atrial fibrillation | 440059007 |
| Atrial fibrillation (disorder) | Atrial fibrillation | 49436004 |
| Atrial flutter (disorder) | Atrial fibrillation | 5370000 |
| Longstanding persistent atrial fibrillation (disorder) | Atrial fibrillation | 706923002 |
| Familial atrial fibrillation (disorder) | Atrial fibrillation | 715395008 |
| Typical atrial flutter (disorder) | Atrial fibrillation | 720448006 |
| Preexcited atrial fibrillation (disorder) | Atrial fibrillation | 762247006 |
| Chronic right-sided heart failure (disorder) | Heart failure | 10335000 |
| Acute congestive heart failure (disorder) | Heart failure | 10633002 |
| Chronic left-sided heart failure (disorder) | Heart failure | 111283005 |
| Left ventricular failure with normal ejection fraction due to valvular heart disease (disorder) | Heart failure | 1204200007 |
| Left ventricular failure with normal ejection fraction due to coronary arteriosclerosis (disorder) | Heart failure | 1204203009 |
| Left ventricular failure with normal ejection fraction due to myocarditis (disorder) | Heart failure | 1204204003 |
| Left ventricular failure with normal ejection fraction due to cardiomyopathy (disorder) | Heart failure | 1204206001 |
| Left ventricular failure with sepsis (disorder) | Heart failure | 1204462004 |
| Right ventricular failure with sepsis (disorder) | Heart failure | 1204468000 |
| Right ventricular failure due to heart valve disorder (disorder) | Heart failure | 1208843003 |
| Right ventricular failure due to disorder of lung (disorder) | Heart failure | 1208846006 |
| Right ventricular failure due to disorder of pulmonary circulation (disorder) | Heart failure | 1208848007 |
| Right ventricular failure due to right ventricular infarction (disorder) | Heart failure | 1208850004 |
| Right heart failure (disorder) | Heart failure | 128404006 |
| Chronic combined systolic and diastolic heart failure (disorder) | Heart failure | 153941000119100 |
| Acute on chronic right-sided congestive heart failure (disorder) | Heart failure | 16838951000119100 |
| Decompensated cardiac failure (disorder) | Heart failure | 195111005 |
| Compensated cardiac failure (disorder) | Heart failure | 195112003 |
| Acute left ventricular failure (disorder) | Heart failure | 195114002 |
| Heart failure as a complication of care (disorder) | Heart failure | 233924009 |
| Refractory heart failure (disorder) | Heart failure | 314206003 |
| Right ventricular failure (disorder) | Heart failure | 367363000 |
| New York Heart Association Classification - Class I (finding) | Heart failure | 420300004 |
| New York Heart Association Classification - Class III (finding) | Heart failure | 420913000 |
| New York Heart Association Classification - Class II (finding) | Heart failure | 421704003 |
| New York Heart Association Classification - Class IV (finding) | Heart failure | 422293003 |
| Congestive heart failure (disorder) | Heart failure | 42343007 |
| Congestive heart failure due to valvular disease (disorder) | Heart failure | 426611007 |
| Rheumatic left ventricular failure (disorder) | Heart failure | 43736008 |
| Chronic systolic heart failure (disorder) | Heart failure | 441481004 |
| Chronic diastolic heart failure (disorder) | Heart failure | 441530006 |
| Heart failure with normal ejection fraction (disorder) | Heart failure | 446221000 |
| Chronic heart failure (disorder) | Heart failure | 48447003 |
| Chronic left-sided congestive heart failure (disorder) | Heart failure | 5375005 |
| Acute heart failure (disorder) | Heart failure | 56675007 |
| Chronic right-sided congestive heart failure (disorder) | Heart failure | 66989003 |
| Heart failure with reduced ejection fraction (disorder) | Heart failure | 703272007 |
| Heart failure with mid range ejection fraction (disorder) | Heart failure | 788950000 |
| Congestive rheumatic heart failure (disorder) | Heart failure | 82523003 |
| Heart failure (disorder) | Heart failure | 84114007 |
| Left heart failure (disorder) | Heart failure | 85232009 |
| Low output heart failure due to and following Fontan operation (disorder) | Heart failure | 871617000 |
| Chronic congestive heart failure (disorder) | Heart failure | 88805009 |
| Heart failure due to thyrotoxicosis (disorder) | Heart failure | 898208007 |
| Biventricular congestive heart failure (disorder) | Heart failure | 92506005 |

# **Supplementary Table 3A. Granular baseline characteristics**

|  | **Oral cavity** | **Oral cavity** | **Oesophageal** | **Oesophageal** | **Stomach** | **Stomach** | **Colorectal** | **Colorectal** |
| --- | --- | --- | --- | --- | --- | --- | --- | --- |
|  | **Control** | **Case** | **Control** | **Case** | **Control** | **Case** | **Control** | **Case** |
| **N** | **N=1764** | **N=441** | **N=492** | **N=123** | **N=656** | **N=164** | **N=6720** | **N=1680** |
| **Age, years** | 61.9(13.9) | 61.9(13.9) | 68.5(11.8) | 68.5(11.9) | 67.4(15) | 67.5(15.2) | 67.7(14) | 67.8(14.1) |
| **Sex, male** | 1056(59.9%) | 264(59.9%) | 292(59.3%) | 73(59.3%) | 408(62.2%) | 102(62.2%) | 3700(55.1%) | 925(55.1%) |
| **Average time from cancer diagnosis, years** | NA | 8.3(7.3) | NA | 7.1 (6.7) | NA | 7.8 (7.0) | NA | 7.5 (6.9) |
| **White** | 638(42.6%) | 170(46.7%) | 195(46.9%) | 51(54.3%) | 231(41.8%) | 53(35.3%) | 2328(41%) | 723(52.1%) |
| **Mixed** | 32(2.1%) | 6(1.6%) | 12(2.9%) | 3(3.2%) | 10(1.8%) | 3(2%) | 145(2.6%) | 31(2.2%) |
| **Asian** | 470(31.4%) | 120(33%) | 129(31%) | 26(27.7%) | 165(29.9%) | 39(26%) | 1757(30.9%) | 320(23.1%) |
| **Black** | 301(20.1%) | 57(15.7%) | 69(16.6%) | 9(9.6%) | 127(23%) | 48(32%) | 1236(21.8%) | 266(19.2%) |
| **Other** | 55(3.7%) | 11(3%) | 11(2.6%) | 5(5.3%) | 19(3.4%) | 7(4.7%) | 212(3.7%) | 47(3.4%) |
| **IMD 1st quintile** | 758(43%) | 198(44.9%) | 219(44.5%) | 59(48%) | 312(47.6%) | 80(48.8%) | 2932(43.7%) | 690(41.1%) |
| **IMD 2nd quintile** | 647(36.7%) | 162(36.7%) | 175(35.6%) | 38(30.9%) | 225(34.4%) | 49(29.9%) | 2417(36%) | 634(37.8%) |
| **IMD 3rd quintile** | 215(12.2%) | 54(12.2%) | 63(12.8%) | 17(13.8%) | 81(12.4%) | 20(12.2%) | 889(13.2%) | 250(14.9%) |
| **IMD 4th quintile** | 110(6.2%) | 20(4.5%) | 31(6.3%) | 8(6.5%) | 27(4.1%) | 12(7.3%) | 372(5.5%) | 77(4.6%) |
| **IMD 5th quintile** | 33(1.9%) | 7(1.6%) | 4(0.8%) | 1(0.8%) | 10(1.5%) | 3(1.8%) | 107(1.6%) | 28(1.7%) |
| **Non-smoker** | 1008(59.4%) | 197(45.8%) | 288(60.8%) | 58(47.2%) | 372(58.5%) | 82(50.31%) | 3875(59.3%) | 943(56.5%) |
| **Ex-smoker** | 390(23%) | 160(37.2%) | 125(26.4%) | 42(34.1%) | 157(24.7%) | 55(33.74%) | 1683(25.8%) | 537(32.2%) |
| **Current smoker** | 299(17.6%) | 73(17%) | 61(12.9%) | 23(18.7%) | 107(16.8%) | 26(15.95%) | 974(14.9%) | 189(11.3%) |
| **Alcohol units/wk** | 3.7(9) | 3.5(8.2) | 2.8(6.5) | 1.8(4.1) | 3.3(8.9) | 1.2(2.8) | 2.8(6.2) | 3.5(18.9) |
| **Alcohol units/wk** | 0[0-2] | 0[0-3] | 0[0-2] | 0[0-1] | 0[0-2] | 0[0-0.1] | 0[0-2] | 0[0-2] |
| **Alcohol misuse, yes** | 33(1.9%) | 17(3.9%) | 3(0.6%) | 3(2.4%) | 14(2.1%) | 4(2.4%) | 129(1.9%) | 34(2%) |
| **BMI, kg/m^2^** | 27.8(5.9) | 25.7(5.6) | 27.5(5.1) | 24.4(5.1) | 27.5(5.4) | 24.7(5.5) | 27.4(5.7) | 27.5(5.8) |
| **SBP, mmHg** | 130.6(13) | 127.8(13.3) | 133.3(13.7) | 126.5(13.1) | 130.2(13.6) | 127.9(13.2) | 131.5(13.1) | 130.6(12.9) |
| **DBP, mmHg** | 77.8(8.4) | 77(8.7) | 77.1(8.8) | 75.6(8.2) | 76.1(8.2) | 75(8.5) | 76.7(8.5) | 76.6(8.5) |

**Supplementary Table 3A Footnote:** This table summarizes the characteristics of participants in each cancer survivor group and their respective control group. N denotes the number of participants; age is presented as mean (standard deviation); sex (male) and racial/ethnic categories (White, Mixed, Asian, Black, Other) are shown as count (percentage of total); IMD (Index of Multiple Deprivation) is represented by the number of participants in each quintile distributions; smoking status is categorized into non-smokers, ex-smokers, and current smokers; alcohol consumption is reported both as mean (SD) units per week and median [interquartile range]; alcohol misuse (yes) indicates individuals reporting consumption levels above recommended limits; BMI is given in kg/m^2 as mean (SD); systolic and diastolic blood pressures (SBP and DBP) are reported in mmHg as mean (SD).

# **Supplementary Table 3B. Granular baseline characteristics**

|  | **Liver** | **Liver** | **Pancreas** | **Pancreas** | **Lung** | **Lung** | **Malignant melanoma** | **Malignant melanoma** |
| --- | --- | --- | --- | --- | --- | --- | --- | --- |
|  | **Control** | **Case** | **Control** | **Case** | **Control** | **Case** | **Control** | **Case** |
| **N** | **N=532** | **N=133** | **N=312** | **N=78** | **N=2480** | **N=620** | **N=4312** | **N=1078** |
| **Age, years** | 64.8(11.8) | 64.8(11.9) | 64(14.2) | 64.1(14.4) | 68.3(12) | 68.4(12.2) | 56.7(16.5) | 56.7(16.6) |
| **Sex, male** | 396(74.4%) | 99(74.4%) | 152(48.7%) | 38(48.7%) | 1348(54.4%) | 337(54.4%) | 1792(41.6%) | 448(41.6%) |
| **Average time from cancer diagnosis, years** | NA | 6.5 (7.5) | NA | 3.3 ( 5.0) | NA | 4.8 ( 5.1) | NA | 9.7 ( 7.9) |
| **White** | 171(39.1%) | 40(35.1%) | 120(44%) | 33(49.3%) | 887(42.2%) | 297(59.9%) | 1792(50.6%) | 725(90.1%) |
| **Mixed** | 14(3.2%) | 2(1.8%) | 8(2.9%) | 5(7.5%) | 66(3.1%) | 11(2.2%) | 107(3%) | 8(1%) |
| **Asian** | 135(30.9%) | 45(39.5%) | 80(29.3%) | 14(20.9%) | 609(29%) | 110(22.2%) | 880(24.8%) | 22(2.7%) |
| **Black** | 95(21.7%) | 25(21.9%) | 59(21.6%) | 12(17.9%) | 462(22%) | 71(14.3%) | 651(18.4%) | 25(3.1%) |
| **Other** | 22(5%) | 2(1.8%) | 6(2.2%) | 3(4.5%) | 76(3.6%) | 7(1.4%) | 113(3.2%) | 25(3.1%) |
| **IMD 1st quintile** | 249(46.8%) | 64(48.5%) | 141(45.2%) | 30(38.5%) | 1159(46.8%) | 284(45.9%) | 1774(41.2%) | 373(34.6%) |
| **IMD 2nd quintile** | 199(37.4%) | 45(34.1%) | 118(37.8%) | 32(41%) | 861(34.7%) | 227(36.7%) | 1489(34.6%) | 355(33%) |
| **IMD 3rd quintile** | 53(10%) | 18(13.6%) | 34(10.9%) | 13(16.7%) | 305(12.3%) | 74(12%) | 645(15%) | 221(20.5%) |
| **IMD 4th quintile** | 27(5.1%) | 4(3%) | 15(4.8%) | 3(3.8%) | 117(4.7%) | 29(4.7%) | 284(6.6%) | 97(9%) |
| **IMD 5th quintile** | 4(0.8%) | 1(0.8%) | 4(1.3%) | 0(0%) | 36(1.5%) | 5(0.8%) | 116(2.7%) | 31(2.9%) |
| **Non-smoker** | 279(55.5%) | 64(48.9%) | 186(60.8%) | 39(50.6%) | 1439(59.3%) | 142(23.2%) | 2483(60.2%) | 608(57.1%) |
| **Ex-smoker** | 142(28.2%) | 46(35.1%) | 72(23.5%) | 24(31.2%) | 631(26%) | 318(51.9%) | 977(23.7%) | 312(29.3%) |
| **Current smoker** | 82(16.3%) | 21(16%) | 48(15.7%) | 14(18.2%) | 355(14.6%) | 153(25%) | 668(16.2%) | 144(13.5%) |
| **Alcohol units/wk** | 2.8(6.2) | 3.5(18.9) | 2.3(5.2) | 2.4(6.4) | 2.4(6.6) | 3.1(9.1) | 3.3(7.9) | 5.4(8) |
| **Alcohol units/wk** | 0[0-2] | 0[0-0] | 0[0-2] | 0[0-1] | 0[0-2] | 0[0-1] | 0[0-4] | 2[0-8] |
| **Alcohol misuse, yes** | 10(1.9%) | 1(0.8%) | 8(2.6%) | 1(1.3%) | 59(2.4%) | 22(3.5%) | 88(2%) | 36(3.3%) |
| **BMI, kg/m^2^** | 27.7(5.6) | 26.5(4.5) | 28.1(6.4) | 24.8(4.8) | 27.9(6) | 25.9(6.1) | 27(5.8) | 26.3(5.4) |
| **SBP, mmHg** | 133(12.6) | 132.7(14.8) | 131.9(13.6) | 129.5(12.5) | 131.7(13) | 128.6(14.1) | 128.6(14.2) | 127.1(13.6) |
| **DBP, mmHg** | 78.8(8.7) | 77.3(9.3) | 78.2(9) | 76.1(9.1) | 76.9(8.6) | 76.1(9) | 77.9(8.5) | 77.1(8.1) |

**Supplementary Table 3B Footnote:** This table summarizes the characteristics of participants in each cancer survivor group and their respective control group. N denotes the number of participants; age is presented as mean (standard deviation); sex (male) and racial/ethnic categories (White, Mixed, Asian, Black, Other) are shown as count (percentage of total); IMD (Index of Multiple Deprivation) is represented by the number of participants in each quintile distributions; smoking status is categorized into non-smokers, ex-smokers, and current smokers; alcohol consumption is reported both as mean (SD) units per week and median [interquartile range]; alcohol misuse (yes) indicates individuals reporting consumption levels above recommended limits; BMI is given in kg/m^2 as mean (SD); systolic and diastolic blood pressures (SBP and DBP) are reported in mmHg as mean (SD).

# **Supplementary Table 3C. Granular baseline characteristics**

|  | **Breast** | **Breast** | **Cervix** | **Cervix** | **Uterus** | **Uterus** | **Ovarian** | **Ovarian** |
| --- | --- | --- | --- | --- | --- | --- | --- | --- |
|  | **Control** | **Case** | **Control** | **Case** | **Control** | **Case** | **Control** | **Case** |
| **N** | **N=22200** | **N=5550** | **N=1700** | **N=425** | **N=2300** | **N=575** | **N=1888** | **N=472** |
| **Age, years** | 16.6(63.8) | 63.8(13.5) | 57.2(15.6) | 57.2(15.7) | 67.9(11.8) | 67.9(11.8) | 58.3(15.5) | 58.3(15.6) |
| **Sex, male** | 132(0.6%) | 33(0.6%) | 0(0%) | 0(0%) | 0(0%) | 0(0%) | 0(0%) | 0(0%) |
| **Average time from cancer diagnosis, years** | NA | 9.2 ( 7.4) | NA | 11.8 ( 11.3) | NA | 8.9 ( 6.7) | NA | 9.2 ( 8.1) |
| **White** | 7614(40.3%) | 2138(46.9%) | 618(42.4%) | 217(62.9%) | 769(39.3%) | 237(48.7%) | 658(40.8%) | 183(46.8%) |
| **Mixed** | 604(3.2%) | 144(3.2%) | 44(3%) | 13(3.8%) | 58(3%) | 15(3.1%) | 48(3%) | 16(4.1%) |
| **Asian** | 5485(29%) | 1120(24.6%) | 407(28%) | 53(15.4%) | 605(30.9%) | 131(26.9%) | 477(29.6%) | 114(29.2%) |
| **Black** | 4469(23.7%) | 989(21.7%) | 324(22.3%) | 50(14.5%) | 466(23.8%) | 96(19.7%) | 361(22.4%) | 58(14.8%) |
| **Other** | 715(3.8%) | 167(3.7%) | 63(4.3%) | 12(3.5%) | 59(3%) | 8(1.6%) | 69(4.3%) | 20(5.1%) |
| **IMD 1st quintile** | 9298(41.9%) | 2173(39.2%) | 750(44.1%) | 184(43.3%) | 1050(45.7%) | 241(41.9%) | 803(42.6%) | 190(40.3%) |
| **IMD 2nd quintile** | 8165(36.8%) | 2132(38.4%) | 627(36.9%) | 158(37.2%) | 833(36.2%) | 196(34.1%) | 672(35.6%) | 176(37.3%) |
| **IMD 3rd quintile** | 3185(14.4%) | 817(14.7%) | 220(12.9%) | 64(15.1%) | 295(12.8%) | 106(18.4%) | 287(15.2%) | 77(16.3%) |
| **IMD 4th quintile** | 1176(5.3%) | 332(6%) | 83(4.9%) | 15(3.5%) | 102(4.4%) | 26(4.5%) | 94(5%) | 23(4.9%) |
| **IMD 5th quintile** | 367(1.7%) | 94(1.7%) | 20(1.2%) | 4(0.9%) | 20(0.9%) | 6(1%) | 30(1.6%) | 6(1.3%) |
| **Non-smoker** | 15669(72.2%) | 3719(67.6%) | 1169(71%) | 232(55.6%) | 1617(71.7%) | 428(75.1%) | 1305(71.8%) | 312(67.2%) |
| **Ex-smoker** | 3749(17.3%) | 1262(22.9%) | 280(17%) | 108(25.9%) | 414(18.4%) | 110(19.3%) | 302(16.6%) | 108(23.3%) |
| **Current smoker** | 2291(10.6%) | 521(9.5%) | 197(12%) | 77(18.5%) | 223(9.9%) | 32(5.6%) | 210(11.6%) | 44(9.5%) |
| **Alcohol units/wk** | 8(1.7) | 2(5.4) | 1.7(4.6) | 2(4.6) | 1.3(4.1) | 0.9(2.6) | 1.7(5.3) | 2(5.4) |
| **Alcohol units/wk** | 0[0-0.6] | 0[0-1] | 0[0-1] | 0[0-2] | 0[0-0] | 0[0-0] | 0[0-1] | 0[0-1] |
| **Alcohol misuse, yes** | 204(0.9%) | 69(1.2%) | 17(1%) | 8(1.9%) | 30(1.3%) | 4(0.7%) | 18(0.9%) | 2(0.4%) |
| **BMI, kg/m^2^** | 5.4(28.3) | 27.9(6.5) | 27.7(6.4) | 27.3(6.1) | 28.5(6.3) | 31.4(8.2) | 27.9(6.5) | 27.6(6.7) |
| **SBP, mmHg** | 13.6(129.8) | 129.1(13.7) | 126.8(14.3) | 127.6(14.9) | 131.6(13.9) | 132.6(13.1) | 128.6(14.4) | 126.7(13.7) |
| **DBP, mmHg** | 8.1(77.2) | 77.5(8.4) | 76.7(8.5) | 77.5(8.8) | 76.9(8.4) | 77.6(8.2) | 77.7(8.2) | 77(8.3) |

**Supplementary Table 3C Footnote:** This table summarizes the characteristics of participants in each cancer survivor group and their respective control group. N denotes the number of participants; age is presented as mean (standard deviation); sex (male) and racial/ethnic categories (White, Mixed, Asian, Black, Other) are shown as count (percentage of total); IMD (Index of Multiple Deprivation) is represented by the number of participants in each quintile distributions; smoking status is categorized into non-smokers, ex-smokers, and current smokers; alcohol consumption is reported both as mean (SD) units per week and median [interquartile range]; alcohol misuse (yes) indicates individuals reporting consumption levels above recommended limits; BMI is given in kg/m^2 as mean (SD); systolic and diastolic blood pressures (SBP and DBP) are reported in mmHg as mean (SD).

# **Supplementary Table 3D. Granular baseline characteristics**

|  | **Prostate** | **Prostate** | **Kidney** | **Kidney** | **Bladder** | **Bladder** | **Brain/CNS** | **Brain/CNS** |
| --- | --- | --- | --- | --- | --- | --- | --- | --- |
|  | **Control** | **Case** | **Control** | **Case** | **Control** | **Case** | **Control** | **Case** |
| **N** | **N=13808** | **N=3452** | **N=2104** | **N=526** | **N=3068** | **N=767** | **N=548** | **N=137** |
| **Age, years** | 72.8(9.9) | 73.1(10.2) | 63.6(14.2) | 63.7(14.3) | 69.7(13) | 69.9(13.2) | 48.6(16.2) | 48.6(16.3) |
| **Sex, male** | 13808(100%) | 3452(100%) | 1296(61.6%) | 324(61.6%) | 2320(75.6%) | 580(75.6%) | 288(52.6%) | 72(52.6%) |
| **Average time from cancer diagnosis, years** | NA | 7.2 ( 5.3) | NA | 6.6 ( 6.5) | NA | 7.2 ( 6.0) | NA | 9.3 ( 7.9) |
| **White** | 4857(42.7%) | 1093(37%) | 704(39.3%) | 204(45.7%) | 1099(43.3%) | 375(59.2%) | 194(42.3%) | 61(50.4%) |
| **Mixed** | 262(2.3%) | 96(3.2%) | 54(3%) | 8(1.8%) | 63(2.5%) | 16(2.5%) | 15(3.3%) | 3(2.5%) |
| **Asian** | 3142(27.6%) | 293(9.9%) | 552(30.8%) | 132(29.6%) | 734(28.9%) | 130(20.5%) | 146(31.8%) | 36(29.8%) |
| **Black** | 2643(23.2%) | 1403(47.5%) | 407(22.7%) | 86(19.3%) | 543(21.4%) | 86(13.6%) | 78(17%) | 17(14%) |
| **Other** | 472(4.1%) | 69(2.3%) | 73(4.1%) | 16(3.6%) | 99(3.9%) | 26(4.1%) | 26(5.7%) | 4(3.3%) |
| **IMD 1st quintile** | 5909(42.8%) | 1486(43.1%) | 931(44.3%) | 248(47.1%) | 1322(43.1%) | 333(43.4%) | 240(43.8%) | 51(37.2%) |
| **IMD 2nd quintile** | 5014(36.3%) | 1245(36.1%) | 730(34.7%) | 182(34.6%) | 1105(36%) | 289(37.7%) | 196(35.8%) | 61(44.5%) |
| **IMD 3rd quintile** | 1894(13.7%) | 471(13.6%) | 298(14.2%) | 62(11.8%) | 415(13.5%) | 100(13%) | 74(13.5%) | 14(10.2%) |
| **IMD 4th quintile** | 756(5.5%) | 183(5.3%) | 117(5.6%) | 29(5.5%) | 180(5.9%) | 32(4.2%) | 28(5.1%) | 10(7.3%) |
| **IMD 5th quintile** | 227(1.6%) | 66(1.9%) | 27(1.3%) | 5(1%) | 46(1.5%) | 13(1.7%) | 10(1.8%) | 1(0.7%) |
| **Non-smoker** | 6808(50.6%) | 1844(53.7%) | 1201(58.8%) | 266(51.1%) | 1614(54.2%) | 279(37.2%) | 327(63.7%) | 86(63.2%) |
| **Ex-smoker** | 4610(34.2%) | 1199(34.9%) | 491(24%) | 159(30.5%) | 887(29.8%) | 282(37.5%) | 92(17.9%) | 28(20.6%) |
| **Current smoker** | 2043(0.2%) | 391(0.1%) | 351(0.2%) | 96(0.2%) | 479(0.2%) | 190(0.3%) | 94(0.2%) | 22(0.2%) |
| **Alcohol units/wk** | 3.7(9) | 3.5(8.2) | 2.9(7.5) | 2.6(8.7) | 3.2(8.1) | 3.6(8.8) | 3.5(9.6) | 2.9(9) |
| **Alcohol units/wk** | 0[0-3] | 0[0-4] | 0[0-2] | 0[0-1] | 0[0-2] | 0[0-3] | 0[0-3] | 0[0-2] |
| **Alcohol misuse, yes** | 313(2.3%) | 55(1.6%) | 46(2.2%) | 10(1.9%) | 64(2.1%) | 15(2%) | 9(1.6%) | 2(1.5%) |
| **BMI, kg/m^2^** | 27.1(5.1) | 27.7(4.8) | 27.7(5.7) | 28.6(6.5) | 27.3(5.4) | 27.3(5.6) | 26.6(5.6) | 27.7(6.8) |
| **SBP, mmHg** | 132.7(12.7) | 132.5(12) | 131.4(12.9) | 131(13.1) | 131.9(12.9) | 130.9(13) | 126.5(13.7) | 124(12.3) |
| **DBP, mmHg** | 76.4(8.5) | 76.6(8.5) | 77.4(8.5) | 78.4(8.9) | 76.7(8.5) | 76.1(8.5) | 78.5(8.7) | 76.6(8.5) |

**Supplementary Table 3D Footnote:** This table summarizes the characteristics of participants in each cancer survivor group and their respective control group. N denotes the number of participants; age is presented as mean (standard deviation); sex (male) and racial/ethnic categories (White, Mixed, Asian, Black, Other) are shown as count (percentage of total); IMD (Index of Multiple Deprivation) is represented by the number of participants in each quintile distributions; smoking status is categorized into non-smokers, ex-smokers, and current smokers; alcohol consumption is reported both as mean (SD) units per week and median [interquartile range]; alcohol misuse (yes) indicates individuals reporting consumption levels above recommended limits; BMI is given in kg/m^2 as mean (SD); systolic and diastolic blood pressures (SBP and DBP) are reported in mmHg as mean (SD).

# **Supplementary Table 3E. Granular baseline characteristics**

|  | **Thyroid** | **Thyroid** | **NHL** | **NHL** | **Multiple myeloma** | **Multiple myeloma** | **Leukaemia** | **Leukaemia** |
| --- | --- | --- | --- | --- | --- | --- | --- | --- |
|  | **Control** | **Case** | **Control** | **Case** | **Control** | **Case** | **Control** | **Case** |
| **N** | **N=2404** | **N=601** | **N=3672** | **N=918** | **N=1300** | **N=325** | **N=3096** | **N=774** |
| **Age, years** | 49.3(14.7) | 49.3(14.7) | 57.6(16.7) | 57.6(16.8) | 68(12.5) | 68.1(12.6) | 52.8(19.2) | 52.9(19.3) |
| **Sex, male** | 632(26.3%) | 158(26.3%) | 2020(55%) | 505(55%) | 692(53.23%) | 173(53.23%) | 1772(57.2%) | 443(57.2%) |
| **Average time from cancer diagnosis, years** | NA | 9.1 ( 7.5) | NA | 8.4( 7.8) | NA | 4.9( 4.9) | NA | 10.4 ( 8.8) |
| **White** | 827(40.6%) | 217(40.1%) | 1280(41.7%) | 341(44.4%) | 447(41.2%) | 99(34.4%) | 1126(42.6%) | 295(46.2%) |
| **Mixed** | 72(3.5%) | 22(4.1%) | 77(2.5%) | 25(3.3%) | 28(2.6%) | 10(3.5%) | 73(2.8%) | 18(2.8%) |
| **Asian** | 680(33.4%) | 213(39.4%) | 929(30.3%) | 225(29.3%) | 321(29.6%) | 51(17.7%) | 825(31.2%) | 195(30.6%) |
| **Black** | 351(17.2%) | 64(11.8%) | 631(20.6%) | 129(16.8%) | 249(22.9%) | 115(39.9%) | 494(18.7%) | 107(16.8%) |
| **Other** | 107(5.3%) | 25(4.6%) | 150(4.9%) | 48(6.3%) | 40(3.7%) | 13(4.5%) | 123(4.7%) | 23(3.6%) |
| **IMD 1st quintile** | 1046(43.5%) | 279(46.4%) | 1590(43.3%) | 384(41.8%) | 548(42.2%) | 129(39.8%) | 1294(41.8%) | 305(39.4%) |
| **IMD 2nd quintile** | 861(35.8%) | 213(35.4%) | 1313(35.8%) | 351(38.2%) | 487(37.5%) | 128(39.5%) | 1153(37.3%) | 311(40.2%) |
| **IMD 3rd quintile** | 313(13%) | 71(11.8%) | 539(14.7%) | 115(12.5%) | 185(14.2%) | 45(13.9%) | 413(13.4%) | 102(13.2%) |
| **IMD 4th quintile** | 132(5.5%) | 28(4.7%) | 180(4.9%) | 57(6.2%) | 62(4.8%) | 17(5.2%) | 180(5.8%) | 43(5.6%) |
| **IMD 5th quintile** | 51(2.1%) | 10(1.7%) | 48(1.3%) | 11(1.2%) | 18(1.4%) | 5(1.5%) | 53(1.7%) | 13(1.7%) |
| **Non-smoker** | 1475(65.6%) | 416(71.1%) | 2140(61.1%) | 554(61.4%) | 787(62.1%) | 212(65.6%) | 1783(62.1%) | 476(64.4%) |
| **Ex-smoker** | 424(18.8%) | 108(18.5%) | 790(22.5%) | 219(24.3%) | 316(24.9%) | 83(25.7%) | 583(20.3%) | 175(23.7%) |
| **Current smoker** | 351(15.6%) | 61(10.4%) | 574(16.4%) | 130(14.4%) | 164(12.9%) | 28(8.7%) | 504(17.6%) | 88(11.9%) |
| **Alcohol units/wk** | 2.3(6.2) | 1.6(3.9) | 3(7.4) | 2.3(6) | 2.6(6.5) | 1.8(4.7) | 2.7(6.6) | 2.8(7.8) |
| **Alcohol units/wk** | 0[0-2] | 0[0-1] | 0[0-2] | 0[0-2] | 0[0-2] | 0[0-0] | 0[0-2] | 0[0-2] |
| **Alcohol misuse, yes** | 37(1.5%) | 6(1%) | 53(1.4%) | 13(1.4%) | 19(1.5%) | 2(0.6%) | 45(1.5%) | 13(1.7%) |
| **BMI, kg/m^2^** | 27.3(6.2) | 27.5(6.8) | 27.3(5.8) | 27.3(5.9) | 28(5.8) | 27.7(5.9) | 26.9(5.8) | 26.7(5.9) |
| **SBP, mmHg** | 125.8(14.1) | 125.2(13.5) | 129.3(13.6) | 127.2(13.8) | 132(13.1) | 130.6(12.4) | 128.6(13.1) | 126.6(13.7) |
| **DBP, mmHg** | 77.7(8.7) | 78.5(8.5) | 77.7(8.6) | 76.9(8.2) | 76.8(8) | 76.5(7.7) | 76.9(8.4) | 75.9(8.7) |

**Supplementary Table 3E Footnote:** This table summarizes the characteristics of participants in each cancer survivor group and their respective control group. N denotes the number of participants; age is presented as mean (standard deviation); sex (male) and racial/ethnic categories (White, Mixed, Asian, Black, Other) are shown as count (percentage of total); IMD (Index of Multiple Deprivation) is represented by the number of participants in each quintile distributions; smoking status is categorized into non-smokers, ex-smokers, and current smokers; alcohol consumption is reported both as mean (SD) units per week and median [interquartile range]; alcohol misuse (yes) indicates individuals reporting consumption levels above recommended limits; BMI is given in kg/m^2 as mean (SD); systolic and diastolic blood pressures (SBP and DBP) are reported in mmHg as mean (SD).

# **Supplementary Table 4. Association between cancer survivorship and cardiovascular disease burden**

|  |  | **Main analysis (imputation)** | | **Sensitivity analysis (complete cases)** | |
| --- | --- | --- | --- | --- | --- |
| **Cancer site** | **Outcome** | **OR (95% CI)** | **p-value** | **OR (95% CI)** | **p-value** |
| **Any cancer** | IHD | 1.13 (1.06, 1.20) | <0.001 | 1.10 (1.03, 1.17) | 0.006 |
|  | Stroke/TIA | 1.13 (1.03, 1.24) | 0.013 | 1.10 (0.99, 1.22) | 0.100 |
|  | Heart Failure | 1.32 (1.21, 1.43) | <0.001 | 1.26 (1.14, 1.38) | <0.001 |
|  | AF | 1.27 (1.18, 1.36) | <0.001 | 1.23 (1.13, 1.34) | <0.001 |
|  | VTE | 2.01 (1.86, 2.17) | <0.001 | 1.96 (1.79, 2.14) | <0.001 |
|  | Diabetes | 1.12 (1.07, 1.17) | <0.001 | 1.10 (1.05, 1.16) | <0.001 |
|  | HT | 1.23 (1.18, 1.28) | <0.001 | 1.20 (1.14, 1.25) | <0.001 |
|  | CKD | 1.37 (1.31, 1.44) | <0.001 | 1.32 (1.25, 1.40) | <0.001 |
| **Oral cavity** | IHD | 1.07 (0.72, 1.58) | 0.848 | 0.97 (0.61, 1.50) | 0.960 |
|  | Stroke/TIA | 1.35 (0.72, 2.44) | 0.523 | 2.04 (1.01, 3.96) | 0.159 |
|  | Heart Failure | 1.01 (0.43, 2.09) | 0.990 | 0.86 (0.30, 2.09) | 0.865 |
|  | AF | 1.57 (0.92, 2.60) | 0.207 | 1.70 (0.90, 3.10) | 0.263 |
|  | VTE | 1.82 (1.01, 3.17) | 0.124 | 1.34 (0.60, 2.75) | 0.684 |
|  | Diabetes | 1.04 (0.77, 1.38) | 0.904 | 1.06 (0.77, 1.46) | 0.824 |
|  | HT | 1.79 (1.39, 2.31) | <0.001 | 1.96 (1.46, 2.66) | <0.001 |
|  | CKD | 1.38 (0.97, 1.95) | 0.163 | 1.28 (0.85, 1.89) | 0.448 |
| **Oesophageal** | IHD | 1.64 (0.87, 3.00) | 0.238 | 1.29 (0.60, 2.61) | 0.717 |
|  | Stroke/TIA | 1.26 (0.46, 3.06) | 0.811 | 0.62 (0.15, 2.00) | 0.684 |
|  | Heart Failure | 0.93 (0.28, 2.52) | 0.949 | 0.59 (0.11, 2.02) | 0.677 |
|  | AF | 1.87 (0.82, 4.03) | 0.243 | 1.72 (0.61, 4.48) | 0.514 |
|  | VTE | 2.94 (1.40, 6.04) | 0.021 | 4.17 (1.78, 9.80) | 0.009 |
|  | Diabetes | 1.48 (0.89, 2.43) | 0.243 | 1.44 (0.81, 2.54) | 0.422 |
|  | HT | 0.81 (0.51, 1.28) | 0.543 | 0.61 (0.35, 1.05) | 0.221 |
|  | CKD | 0.87 (0.47, 1.57) | 0.822 | 0.96 (0.48, 1.85) | 0.960 |
| **Stomach** | IHD | 1.07 (0.60, 1.85) | 0.904 | 1.19 (0.63, 2.18) | 0.773 |
|  | Stroke/TIA | 2.20 (0.94, 4.96) | 0.161 | 1.56 (0.58, 3.88) | 0.598 |
|  | Heart Failure | 0.73 (0.27, 1.69) | 0.660 | 0.71 (0.24, 1.78) | 0.714 |
|  | AF | 1.22 (0.59, 2.41) | 0.766 | 1.27 (0.56, 2.67) | 0.748 |
|  | VTE | 2.42 (0.99, 5.61) | 0.136 | 2.46 (0.85, 6.63) | 0.263 |
|  | Diabetes | 1.51 (0.98, 2.32) | 0.152 | 1.47 (0.93, 2.33) | 0.263 |
|  | HT | 1.61 (1.05, 2.49) | 0.095 | 1.50 (0.93, 2.44) | 0.263 |
|  | CKD | 1.14 (0.67, 1.92) | 0.799 | 1.16 (0.64, 2.06) | 0.783 |
| **Colorectal** | IHD | 1.15 (0.96, 1.38) | 0.238 | 1.14 (0.92, 1.39) | 0.444 |
|  | Stroke/TIA | 1.31 (0.97, 1.73) | 0.166 | 1.15 (0.82, 1.60) | 0.649 |
|  | Heart Failure | 1.38 (1.07, 1.77) | 0.053 | 1.48 (1.10, 1.97) | 0.047 |
|  | AF | 1.30 (1.05, 1.61) | 0.058 | 1.13 (0.87, 1.45) | 0.598 |
|  | VTE | 2.68 (2.15, 3.32) | <0.001 | 2.63 (2.04, 3.38) | <0.001 |
|  | Diabetes | 1.33 (1.16, 1.52) | <0.001 | 1.32 (1.14, 1.52) | 0.003 |
|  | HT | 1.24 (1.09, 1.40) | 0.005 | 1.16 (1.00, 1.33) | 0.154 |
|  | CKD | 1.31 (1.13, 1.52) | 0.003 | 1.21 (1.02, 1.43) | 0.109 |
| **Liver** | IHD | 1.28 (0.67, 2.37) | 0.628 | 0.85 (0.39, 1.75) | 0.818 |
|  | Stroke/TIA | 1.20 (0.50, 2.63) | 0.824 | 1.31 (0.53, 2.99) | 0.744 |
|  | Heart Failure | 2.38 (1.04, 5.28) | 0.115 | 1.82 (0.62, 4.97) | 0.480 |
|  | AF | 1.86 (0.86, 3.84) | 0.225 | 2.04 (0.83, 4.84) | 0.286 |
|  | VTE | 1.76 (0.32, 7.22) | 0.660 | 4.06 (0.63, 29.79) | 0.311 |
|  | Diabetes | 1.87 (1.20, 2.90) | 0.025 | 2.25 (1.37, 3.71) | 0.008 |
|  | HT | 2.28 (1.47, 3.57) | 0.002 | 2.32 (1.41, 3.86) | 0.008 |
|  | CKD | 1.50 (0.87, 2.55) | 0.255 | 1.63 (0.90, 2.92) | 0.267 |
| **Pancreas** | IHD | 2.34 (0.97, 5.46) | 0.152 | 2.46 (0.91, 6.46) | 0.222 |
|  | Stroke/TIA | 1.13 (0.20, 4.49) | 0.939 | 0.82 (0.08, 4.17) | 0.929 |
|  | Heart Failure | NC (0.00, 0.00) | <0.001 | NC (0.00, 0.00) | <0.001 |
|  | AF | 5.05 (1.57, 17.27) | 0.028 | 1.85 (0.40, 7.76) | 0.649 |
|  | VTE | 3.23 (1.05, 9.56) | 0.116 | 1.87 (0.31, 8.81) | 0.684 |
|  | Diabetes | 4.01 (2.21, 7.39) | <0.001 | 2.81 (1.45, 5.50) | 0.013 |
|  | HT | 1.93 (1.09, 3.44) | 0.078 | 1.64 (0.86, 3.13) | 0.311 |
|  | CKD | 1.06 (0.44, 2.37) | 0.949 | 0.57 (0.19, 1.50) | 0.480 |
| **Lung** | IHD | 1.44 (1.09, 1.87) | 0.035 | 1.34 (0.99, 1.81) | 0.191 |
|  | Stroke/TIA | 1.56 (1.00, 2.39) | 0.136 | 1.52 (0.90, 2.51) | 0.280 |
|  | Heart Failure | 1.49 (1.01, 2.18) | 0.124 | 1.31 (0.82, 2.04) | 0.474 |
|  | AF | 2.44 (1.71, 3.48) | <0.001 | 1.86 (1.19, 2.86) | 0.032 |
|  | VTE | 4.00 (2.66, 6.03) | <0.001 | 3.19 (1.92, 5.26) | <0.001 |
|  | Diabetes | 1.25 (0.99, 1.57) | 0.153 | 1.23 (0.95, 1.59) | 0.280 |
|  | HT | 1.40 (1.13, 1.73) | 0.010 | 1.60 (1.25, 2.05) | 0.002 |
|  | CKD | 1.23 (0.95, 1.60) | 0.231 | 1.23 (0.91, 1.66) | 0.371 |
| **Melanoma** | IHD | 1.07 (0.75, 1.50) | 0.848 | 1.29 (0.85, 1.92) | 0.443 |
|  | Stroke/TIA | 0.95 (0.52, 1.63) | 0.931 | 1.14 (0.58, 2.11) | 0.818 |
|  | Heart Failure | 1.15 (0.69, 1.87) | 0.766 | 1.06 (0.55, 1.93) | 0.952 |
|  | AF | 1.04 (0.71, 1.49) | 0.931 | 0.99 (0.62, 1.53) | 0.983 |
|  | VTE | 1.26 (0.80, 1.94) | 0.485 | 1.24 (0.70, 2.11) | 0.684 |
|  | Diabetes | 0.95 (0.72, 1.23) | 0.848 | 1.01 (0.74, 1.38) | 0.983 |
|  | HT | 1.10 (0.90, 1.35) | 0.527 | 1.08 (0.84, 1.39) | 0.744 |
|  | CKD | 1.08 (0.81, 1.43) | 0.793 | 0.99 (0.69, 1.41) | 0.983 |
| **Breast** | IHD | 1.03 (0.90, 1.18) | 0.822 | 1.03 (0.89, 1.20) | 0.818 |
|  | Stroke/TIA | 1.10 (0.90, 1.33) | 0.543 | 1.12 (0.90, 1.39) | 0.542 |
|  | Heart Failure | 1.38 (1.16, 1.64) | 0.002 | 1.43 (1.18, 1.73) | 0.003 |
|  | AF | 1.18 (1.01, 1.38) | 0.100 | 1.26 (1.05, 1.50) | 0.053 |
|  | VTE | 1.86 (1.61, 2.15) | <0.001 | 1.90 (1.60, 2.25) | <0.001 |
|  | Diabetes | 1.07 (0.98, 1.16) | 0.225 | 1.08 (0.99, 1.18) | 0.263 |
|  | HT | 1.09 (1.01, 1.17) | 0.076 | 1.06 (0.98, 1.15) | 0.337 |
|  | CKD | 1.09 (0.99, 1.20) | 0.171 | 1.08 (0.97, 1.21) | 0.328 |
| **Cervix** | IHD | 1.19 (0.66, 2.04) | 0.751 | 1.23 (0.65, 2.24) | 0.729 |
|  | Stroke/TIA | 1.69 (0.79, 3.41) | 0.309 | 1.27 (0.50, 2.89) | 0.773 |
|  | Heart Failure | 1.84 (0.93, 3.51) | 0.171 | 1.46 (0.62, 3.19) | 0.598 |
|  | AF | 1.13 (0.59, 2.07) | 0.848 | 0.92 (0.42, 1.87) | 0.925 |
|  | VTE | 2.75 (1.49, 4.98) | 0.010 | 3.18 (1.55, 6.43) | 0.013 |
|  | Diabetes | 1.06 (0.74, 1.49) | 0.860 | 0.94 (0.63, 1.37) | 0.851 |
|  | HT | 1.10 (0.82, 1.48) | 0.721 | 1.00 (0.71, 1.41) | 0.998 |
|  | CKD | 1.77 (1.21, 2.55) | 0.013 | 1.56 (1.00, 2.40) | 0.165 |
| **Uterine** | IHD | 0.97 (0.66, 1.39) | 0.932 | 1.04 (0.70, 1.52) | 0.939 |
|  | Stroke/TIA | 1.03 (0.60, 1.69) | 0.960 | 1.12 (0.64, 1.87) | 0.818 |
|  | Heart Failure | 1.23 (0.72, 2.03) | 0.628 | 1.46 (0.82, 2.49) | 0.400 |
|  | AF | 1.40 (0.91, 2.12) | 0.241 | 1.57 (0.96, 2.51) | 0.221 |
|  | VTE | 1.76 (1.15, 2.65) | 0.037 | 2.13 (1.33, 3.38) | 0.013 |
|  | Diabetes | 1.36 (1.09, 1.69) | 0.028 | 1.28 (1.01, 1.63) | 0.151 |
|  | HT | 1.12 (0.91, 1.39) | 0.478 | 1.19 (0.93, 1.51) | 0.351 |
|  | CKD | 1.28 (0.99, 1.64) | 0.153 | 1.22 (0.92, 1.62) | 0.359 |
| **Ovarian** | IHD | 1.10 (0.64, 1.82) | 0.848 | 0.98 (0.52, 1.76) | 0.983 |
|  | Stroke/TIA | 1.01 (0.41, 2.19) | 0.990 | 0.73 (0.23, 1.86) | 0.744 |
|  | Heart Failure | 1.02 (0.43, 2.19) | 0.990 | 0.70 (0.21, 1.88) | 0.717 |
|  | AF | 0.89 (0.45, 1.65) | 0.848 | 0.79 (0.34, 1.66) | 0.744 |
|  | VTE | 5.72 (3.54, 9.32) | <0.001 | 5.54 (3.17, 9.79) | <0.001 |
|  | Diabetes | 1.00 (0.73, 1.36) | 0.990 | 0.97 (0.69, 1.35) | 0.939 |
|  | HT | 1.19 (0.91, 1.54) | 0.341 | 1.15 (0.85, 1.55) | 0.598 |
|  | CKD | 1.59 (1.12, 2.23) | 0.037 | 1.69 (1.13, 2.50) | 0.054 |
| **Prostate** | IHD | 1.09 (0.98, 1.22) | 0.228 | 1.07 (0.95, 1.22) | 0.480 |
|  | Stroke/TIA | 1.01 (0.83, 1.22) | 0.955 | 0.99 (0.80, 1.23) | 0.983 |
|  | Heart Failure | 1.07 (0.91, 1.26) | 0.579 | 0.95 (0.78, 1.15) | 0.773 |
|  | AF | 1.12 (0.97, 1.28) | 0.225 | 1.09 (0.93, 1.27) | 0.530 |
|  | VTE | 1.28 (1.06, 1.54) | 0.040 | 1.22 (0.98, 1.50) | 0.222 |
|  | Diabetes | 0.93 (0.85, 1.02) | 0.227 | 0.92 (0.84, 1.02) | 0.280 |
|  | HT | 1.27 (1.17, 1.38) | <0.001 | 1.21 (1.10, 1.33) | 0.002 |
|  | CKD | 1.22 (1.11, 1.35) | <0.001 | 1.13 (1.01, 1.26) | 0.109 |
| **Kidney** | IHD | 1.16 (0.83, 1.59) | 0.553 | 1.08 (0.74, 1.54) | 0.818 |
|  | Stroke/TIA | 0.93 (0.51, 1.60) | 0.902 | 1.02 (0.52, 1.87) | 0.983 |
|  | Heart Failure | 1.27 (0.76, 2.05) | 0.527 | 1.19 (0.68, 2.01) | 0.744 |
|  | AF | 1.60 (1.01, 2.47) | 0.122 | 1.45 (0.86, 2.38) | 0.344 |
|  | VTE | 3.15 (2.01, 4.91) | <0.001 | 2.59 (1.51, 4.38) | 0.008 |
|  | Diabetes | 1.40 (1.10, 1.76) | 0.021 | 1.39 (1.08, 1.78) | 0.053 |
|  | HT | 2.10 (1.68, 2.64) | <0.001 | 2.28 (1.76, 2.95) | <0.001 |
|  | CKD | 6.20 (4.83, 7.99) | <0.001 | 6.00 (4.55, 7.95) | <0.001 |
| **Bladder** | IHD | 1.50 (1.20, 1.87) | 0.003 | 1.37 (1.06, 1.77) | 0.067 |
|  | Stroke/TIA | 1.00 (0.64, 1.52) | 0.990 | 0.89 (0.52, 1.46) | 0.816 |
|  | Heart Failure | 1.45 (1.02, 2.03) | 0.112 | 1.45 (0.97, 2.12) | 0.221 |
|  | AF | 1.56 (1.17, 2.08) | 0.013 | 1.58 (1.12, 2.21) | 0.051 |
|  | VTE | 1.79 (1.25, 2.54) | 0.010 | 1.91 (1.24, 2.91) | 0.025 |
|  | Diabetes | 1.20 (0.99, 1.46) | 0.153 | 1.13 (0.91, 1.40) | 0.495 |
|  | HT | 1.31 (1.10, 1.57) | 0.013 | 1.25 (1.02, 1.54) | 0.127 |
|  | CKD | 1.85 (1.50, 2.28) | <0.001 | 1.76 (1.38, 2.24) | <0.001 |
| **Brain** | IHD | 1.55 (0.51, 4.28) | 0.605 | 1.31 (0.36, 4.11) | 0.818 |
|  | Stroke/TIA | 4.28 (1.34, 14.22) | 0.053 | 3.99 (1.13, 15.22) | 0.122 |
|  | Heart Failure | NC (0.00, 0.00) | <0.001 | NC (0.00, 0.00) | <0.001 |
|  | AF | 1.80 (0.41, 6.52) | 0.589 | NC (0.00, 0.00) | <0.001 |
|  | VTE | 1.80 (0.58, 5.02) | 0.478 | 1.87 (0.51, 6.22) | 0.553 |
|  | Diabetes | 1.11 (0.62, 1.94) | 0.848 | 1.05 (0.56, 1.91) | 0.956 |
|  | HT | 1.15 (0.65, 2.01) | 0.799 | 1.00 (0.52, 1.90) | 0.998 |
|  | CKD | 1.22 (0.40, 3.39) | 0.848 | 1.66 (0.53, 4.89) | 0.598 |
|  | IHD | 1.55 (0.92, 2.52) | 0.209 | 1.43 (0.81, 2.43) | 0.422 |
| **Thyroid** | Stroke/TIA | 2.00 (0.86, 4.33) | 0.217 | 1.31 (0.48, 3.15) | 0.769 |
|  | Heart Failure | 2.13 (0.95, 4.52) | 0.156 | 2.30 (1.02, 4.95) | 0.158 |
|  | AF | 3.54 (1.79, 6.93) | 0.003 | 4.01 (1.81, 8.91) | 0.008 |
|  | VTE | 1.06 (0.52, 1.99) | 0.939 | 1.13 (0.53, 2.21) | 0.851 |
|  | Diabetes | 1.06 (0.78, 1.42) | 0.848 | 1.00 (0.72, 1.37) | 0.998 |
|  | HT | 1.50 (1.15, 1.94) | 0.013 | 1.41 (1.06, 1.87) | 0.078 |
|  | CKD | 2.04 (1.40, 2.95) | 0.002 | 1.79 (1.18, 2.68) | 0.032 |
| **NHL** | IHD | 1.32 (0.99, 1.75) | 0.153 | 1.33 (0.95, 1.82) | 0.263 |
|  | Stroke/TIA | 1.33 (0.83, 2.06) | 0.396 | 1.37 (0.81, 2.23) | 0.451 |
|  | Heart Failure | 2.12 (1.44, 3.09) | 0.002 | 1.76 (1.12, 2.71) | 0.062 |
|  | AF | 1.77 (1.23, 2.51) | 0.010 | 1.71 (1.11, 2.58) | 0.067 |
|  | VTE | 3.36 (2.36, 4.77) | <0.001 | 3.50 (2.31, 5.30) | <0.001 |
|  | Diabetes | 1.16 (0.94, 1.42) | 0.318 | 1.16 (0.92, 1.45) | 0.418 |
|  | HT | 1.06 (0.88, 1.28) | 0.738 | 1.05 (0.85, 1.30) | 0.816 |
|  | CKD | 1.61 (1.26, 2.06) | 0.002 | 1.74 (1.32, 2.29) | 0.002 |
| **Multiple myeloma** | IHD | 1.12 (0.72, 1.69) | 0.791 | 1.10 (0.68, 1.74) | 0.818 |
|  | Stroke/TIA | 0.91 (0.43, 1.79) | 0.902 | 0.54 (0.19, 1.28) | 0.363 |
|  | Heart Failure | 2.38 (1.40, 3.96) | 0.010 | 2.32 (1.30, 4.06) | 0.030 |
|  | AF | 1.57 (0.96, 2.51) | 0.166 | 1.51 (0.85, 2.61) | 0.344 |
|  | VTE | 3.17 (1.91, 5.23) | <0.001 | 2.78 (1.57, 4.89) | 0.008 |
|  | Diabetes | 0.94 (0.68, 1.27) | 0.843 | 0.82 (0.59, 1.15) | 0.475 |
|  | HT | 1.40 (1.06, 1.85) | 0.061 | 1.40 (1.02, 1.92) | 0.132 |
|  | CKD | 2.40 (1.76, 3.28) | <0.001 | 2.32 (1.63, 3.29) | <0.001 |
| **Leukaemia** | IHD | 1.22 (0.87, 1.69) | 0.416 | 1.08 (0.73, 1.57) | 0.818 |
|  | Stroke/TIA | 1.44 (0.82, 2.43) | 0.341 | 1.38 (0.72, 2.51) | 0.553 |
|  | Heart Failure | 2.22 (1.43, 3.40) | 0.003 | 2.07 (1.24, 3.39) | 0.032 |
|  | AF | 1.23 (0.83, 1.81) | 0.478 | 0.96 (0.59, 1.53) | 0.952 |
|  | VTE | 2.49 (1.62, 3.79) | <0.001 | 2.17 (1.31, 3.51) | 0.019 |
|  | Diabetes | 1.55 (1.22, 1.96) | 0.002 | 1.54 (1.19, 1.99) | 0.008 |
|  | HT | 1.26 (1.01, 1.57) | 0.115 | 1.09 (0.85, 1.41) | 0.717 |
|  | CKD | 2.21 (1.66, 2.92) | <0.001 | 1.73 (1.26, 2.37) | 0.008 |

**Supplementary Table 4 Footnote:** Cardiovascular disease (CVD) outcomes among survivors of the 20 most common cancers. The data were derived from fully adjusted Firth logistic regression models along with a complete-case sensitivity analysis. ORs along with 95% confidence intervals were calculated to evaluate the association between cancer survivorship and burden of CVD. We report FDR values to account for multiple testing. Abbreviations: IHD, ischaemic heart disease; TIA, transient ischaemic attack; HF, heart failure; AF, atrial fibrillation; VTE, venous thromboembolism; OR, odds ratio; FDR, false discovery rate.

# **Supplementary Table 5. Association of cancer with prevalent venous thromboembolism stratified by ethnicity**

| **Ethnicity** | **OR (95% CI)** | **p-value** |
| --- | --- | --- |
| **White** | 1.89 (1.70-2.09) | p<0.0001 |
| **Asian** | 2.82 (2.31-3.43) | p<0.0001 |
| **Black** | 2.02 (1.73-2.34) | p<0.0001 |
| **Other** | 1.61 (1.11-2.27) | p=0.012 |

**Supplementary Table 5 Footnote:** Results are associations of cancer with VTE in patients with any cancer compared to controls stratified by ethnicity OR: Odds ratios (OR) are reported along with 95% confidence intervals (CIs) and p-values

# **Supplementary Table 6. Mean serum serum HDL-C levels in survivors of oral cancer history compared to matched controls stratified by ethnicity**

| **Ethnicity** | **Controls**  **Mean(SD)** | **Cancer survivors**  **Mean(SD)** | **FDR adjusted p value** |
| --- | --- | --- | --- |
| **White** | 1.33 (0.33) | 1.55 (0.49) | 0.005 |
| **Asian** | 1.22 (0.22) | 1.25 (0.24) | 0.64 |
| **Black** | 1.44 (0.37) | 1.20 (0.18) | 0.22 |
| **Other** | 1.20 (0.16) | 2.55 (2.19) | 0.03 |

**Supplementary Table 6 footnote.** The table presents the mean serum HDL-C levels (mmol/L) with SDs among controls and cancer survivors, stratified by ethnicity; and a multiple testing corrected test of heterogeneity. FDR: false discovery rate. HDL-C: high density lipoprotein cholesterol; SD: standard deviation.

# **Supplementary Table 7. Mean serum serum LDL-C levels in survivors of bladder cancer history compared to matched controls stratified by ethnicity**

| **Ethnicity** | **Controls**  **Mean (SD)** | **Cancer survivors**  **Mean (SD)** | **FDR adjusted p value** |
| --- | --- | --- | --- |
| **White** | 2.03 (0.86) | 2.04 (0.80) | 0.942 |
| **Asian** | 1.97 (0.85) | 1.94 (0.77) | 0.825 |
| **Black** | 2.07 (0.90) | 3.25 (1.10) | 0.005 |
| **Other** | 2.19 (0.85) | 2.19 (0.81) | 0.99 |

**Supplementary Table 7 footnote.** The table presents the mean serum LDL-C levels (mmol/L) with standard deviations among controls and cancer survivors, stratified by ethnicity; and a multiple testing corrected test of heterogeneity. FDR: false discovery rate. LDL-C: low density lipoprotein cholesterol; SD: standard deviation.
