# Supplementary material for: Cardiovascular disease burden and risk factor management in cancer survivors: insights into a multiethnic, socioeconomically deprived urban population
Source: Heart. 2025 Mar 13;112(1):e325309. doi: 10.1136/heartjnl-2024-325309 (PMC12703254; doi:10.1136/heartjnl-2024-325309)
Supplement: online supplemental file 2 [file heartjnl-112-1-s002.pdf]

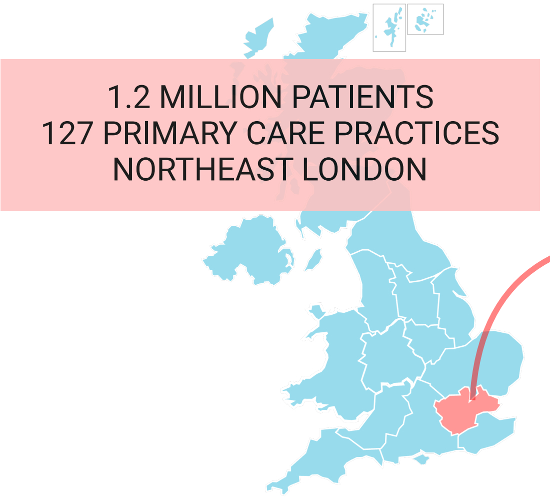

18,839
CANCER SURVIVORS

75,356
MATCHED CONTROLS

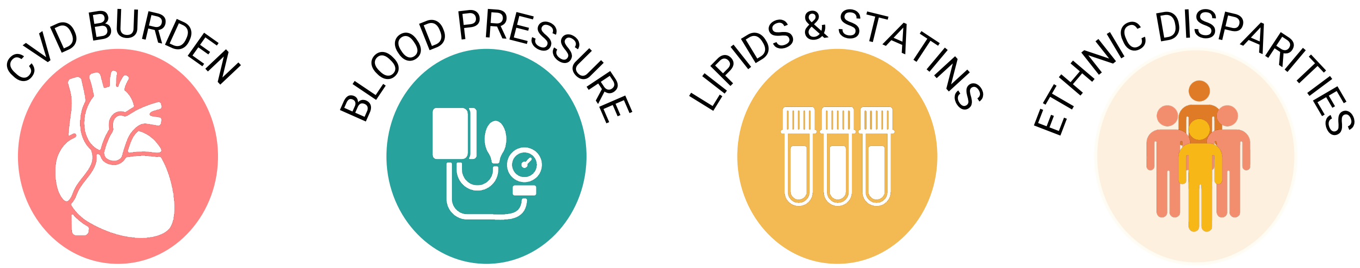

EXCESS BURDEN OF ALL CVDs IN SURVIVORS OF ANY CANCER
HIGHEST RISK CANCERS: **BLOOD, LUNG, BLADDER**

1 IN 3 CANCER SURVIVORS DID NOT MEET BP TARGETS
AS PER GUIDELINES

BLACK AND ASIAN SURVIVORS FACE DISPARITIES
IN CARDIOVASCULAR RISK AND MANAGEMENT
